# microRNA-17 family promotes polycystic kidney disease progression through modulation of mitochondrial metabolism

**DOI:** 10.1038/ncomms14395

**Published:** 2017-02-16

**Authors:** Sachin Hajarnis, Ronak Lakhia, Matanel Yheskel, Darren Williams, Mehran Sorourian, Xueqing Liu, Karam Aboudehen, Shanrong Zhang, Kara Kersjes, Ryan Galasso, Jian Li, Vivek Kaimal, Steven Lockton, Scott Davis, Andrea Flaten, Joshua A. Johnson, William L. Holland, Christine M. Kusminski, Philipp E. Scherer, Peter C. Harris, Marie Trudel, Darren P. Wallace, Peter Igarashi, Edmund C. Lee, John R. Androsavich, Vishal Patel

**Affiliations:** 1 Department of Internal Medicine and Division of Nephrology, University of Texas Southwestern Medical Center, Dallas, Texas 75390, USA; 2 Regulus Therapeutics Inc., San Diego, California 92121, USA; 3 Department of Medicine and Division of Nephrology, University of Minnesota Medical School, Minneapolis, Minnesota 55455, USA; 4 Advanced Imaging Research Center, University of Texas Southwestern Medical Center, Dallas, Texas 75390, USA; 5 Department of Internal Medicine and Touchstone Diabetes Center, University of Texas Southwestern Medical Center, Dallas, Texas 75390, USA; 6 Department of Nephrology and Hypertension, Mayo College of Medicine, Rochester, Minnesota 55905, USA; 7 Molecular Genetics and Development, Institut de Recherches Cliniques de Montreal, Universite de Montreal, Faculte de Medecine, Montréal, Québec H2W 1R7, Canada; 8 Department of Medicine and the Kidney Institute, University of Kansas Medical Center, Kansas City, Kansas 66160, USA

## Abstract

Autosomal dominant polycystic kidney disease (ADPKD) is the most frequent genetic cause of renal failure. Here we identify miR-17 as a target for the treatment of ADPKD. We report that miR-17 is induced in kidney cysts of mouse and human ADPKD. Genetic deletion of the miR-17∼92 cluster inhibits cyst proliferation and PKD progression in four orthologous, including two long-lived, mouse models of ADPKD. Anti-miR-17 treatment attenuates cyst growth in short-term and long-term PKD mouse models. miR-17 inhibition also suppresses proliferation and cyst growth of primary ADPKD cysts cultures derived from multiple human donors. Mechanistically, c-Myc upregulates miR-17∼92 in cystic kidneys, which in turn aggravates cyst growth by inhibiting oxidative phosphorylation and stimulating proliferation through direct repression of *Pparα*. Thus, miR-17 family is a promising drug target for ADPKD, and miR-17-mediated inhibition of mitochondrial metabolism represents a potential new mechanism for ADPKD progression.

## Abstract (web summary)

Autosomal dominant polycystic kidney disease (ADPKD) is a life-threatening genetic disease that leads to renal failure. Here Hajarnis *et al*. show that miR-17 modulates cyst progression in ADPKD through metabolic reprogramming of mitochondria and its inhibition slows cyst development and improves renal functions.

ADPKD, caused by mutations of *PKD1* or *PKD2*, is among the most common monogenetic disorders and a leading genetic cause of end-stage renal disease^1^,^2^,^3^. The clinical hallmark of this disease is the presence of numerous renal tubule-derived cysts. Excessive proliferation, a central pathological feature, fuels the relentless expansion of cysts ultimately causing end-stage renal disease.

MicroRNAs (miRNAs) are non-coding RNAs that bind to complementary sequences located in target mRNAs and inhibit their expression^4^. Aberrant activation of miRNAs promotes the progression of many common diseases^5^,^6^,^7^,^8^. Accordingly, synthetic inhibitors of miRNAs (anti-miRs) have emerged as novel therapeutic agents. Anti-miRs appear to be well tolerated in human clinical trials^9^, have a long duration of action and are efficiently delivered to the liver and kidney^10^,^11^. These attributes make them an extremely attractive drug class for diseases that primarily affect the kidney and liver and require long-term therapy, such as ADPKD. However, whether a miRNA-based therapeutics approach can be applied to ADPKD is not known.

We have previously shown that miR-17∼92 inhibits *PKD1* and *PKD2* and aggravates disease progression in a non-orthologous ciliopathy (*Kif3a-*KO) model of cystic kidney disease^12^. However, the relevance of this finding to ADPKD is unclear because whether miR-17 can modulate cyst growth when *Pkd1* or *Pkd2* are already mutated has not been studied. In fact, with the exception of miR-21 (ref. 13), the role of miRNAs in ADPKD still remains unexplored. Our primary objective was to identify miRNAs that play a pathogenic role in ADPKD. We began by systematically examining miRNA expression profiles in *Pkd1-*KO and *Pkd2*-KO mice. Intriguingly, we found that among upregulated miRNAs, the miR-17 miRNA family contributed most substantially to the total dysregulated miRNA pool in both ADPKD models. These unbiased microarray results and the previous observations in the *Kif3a*-KO mice prompted us to study the role of miR-17 in ADPKD. Using complementary genetic and pharmaceutical approaches, we now show that miR-17∼92 promotes cyst proliferation and ADPKD progression. miR-17 mediates these effects by reprogramming mitochondrial metabolism through direct repression of *Pparα.*

## Results

### miR-17 miRNA family is upregulated in ADPKD

**Figure 1:**
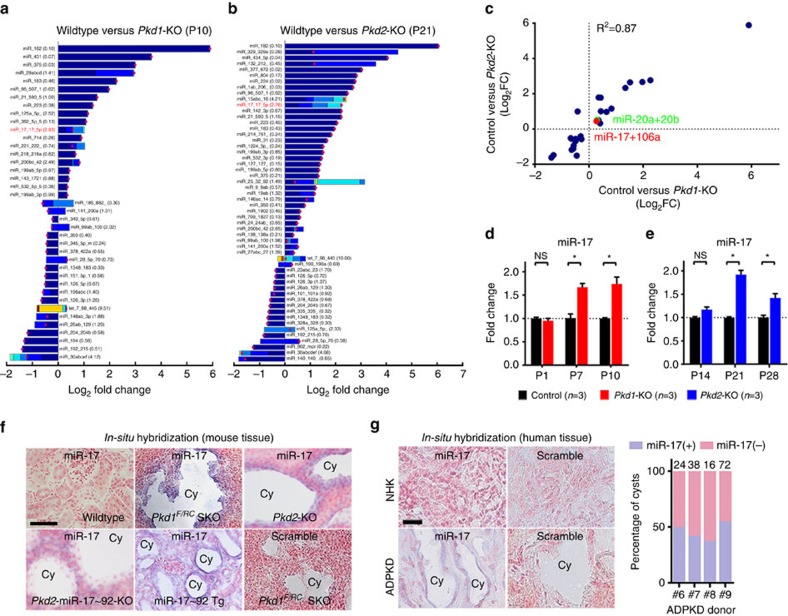
miR-17 is upregulated in kidney cysts of mouse and human ADPKD. Nanostring nCounter microarray analysis was performed to compare miRNA expression patterns between 10-day-old wild type (*n*=3) and *Pkd1*-KO kidneys (*n*=3), and 21-day-old wild type (*n*=3) and *Pkd2*-KO kidneys (*n*=3). (**a**,**b**) The differentially expressed miRNA families in *Pkd1*-KO and *Pkd2*-KO are shown. The red circles mark Log_2_ fold change when only differentially expressed family members are included. The stacked bars mark Log_2_ fold change when all expressed family members irrespective of statistical significance are included. The values in parenthesis indicate the percentage contribution of each miRNA family to the total miRNA pool in cystic kidneys. The miR-17 family is highlighted in red. (**c**) Dysregulated miRNAs exhibited correlated expression pattern (*R*^2^=0.87) between *Pkd1*-KO and *Pkd2*-KO kidneys suggesting that a common set of aberrantly expressed miRNAs may underlie ADPKD pathogenesis. (**d**,**e**) Q-PCR analysis demonstrated that miR-17 is upregulated at cystic stages (P7 & P10 for *Pkd1*-KO and P21 & P28 for *Pkd2*-KO) but not at pre-cystic stages (P1 for *Pkd1*-KO and P14 for *Pkd2*-KO). *In situ* hybridization (ISH) was performed using an LNA-modified anti-miR-17 probe. The kidney sections were counterstained with nuclear fast red to mark nuclei. (**f**) Representative ISH images of kidney sections from wild type, *Pkd1*^F/RC^SKO, *Pkd2*-KO, *Pkd2*-miR-17∼92KO mice (negative control) and miR-17∼92Tg (positive control) are shown. Expression of miR-17 (blue) was increased in cysts (cy) of *Pkd1*^F/RC^SKO and *Pkd2*-KO compared with renal tubules of wild-type mice. miR-17 expression was abolished in cysts of *Pkd2*-miR-17∼92KO mice, whereas its expression was induced in renal tubules of miR-17∼92Tg mice, indicating that the *in situ* probe specifically detects miR-17. (**g**) ISH was performed on kidney sections from four NHK and ADPKD samples. Representative ISH images from one NHK and ADPKD sample are shown. miR-17 expression was not detected by ISH in NHK kidney sections. The percentage of miR-17-positive cysts per kidney section from four human ADPKD patients (#6–9) is shown in the graph. The total number of cysts per section is shown above each bar. P indicates postnatal day. Error bars indicate s.e.m. * indicates *P*<0.05, ns indicates *P*>0.05. Student's unpaired *t-*test (**d**,**e**). Scale bars, 50 μm (**f**) and 20 μm (**g**).

We used Nanostring nCounter microarrays to compare miRNA expression patterns between 10-day-old wild type and Ksp/Cre;*Pkd1*^F/F^ (*Pkd1*-KO) kidneys, and 21-day-old wild type and Pkhd1/Cre;*Pkd2*^F/F^ (*Pkd2*-KO) kidneys (*n*=3 biological replicates for all groups). Forty-five miRNAs were differentially expressed in *Pkd1*-KO kidney, whereas 70 miRNAs were differentially expressed in *Pkd2*-KO kidneys (Supplementary Table 1). Twenty-nine miRNAs were dysregulated in both ADPKD models (Supplementary Table 2). These dysregulated miRNAs exhibited a correlated expression pattern (*R*^2^=0.87) between *Pkd1*-KO and *Pkd2*-KO kidneys suggesting that a common set of aberrantly expressed miRNAs may underlie ADPKD pathogenesis (Fig. 1a–c).

To identify groups of upregulated miRNAs with the highest pathogenic potential, individual differentially expressed miRNAs belonging to the same seed family were categorized together, and a cumulative fold change for each family was calculated. Among the upregulated miRNAs, we focused on the miR-17 family because it contributed most substantially (>2.7%) to the total miRNA pool in both ADPKD models (Fig. 1a,b). Quantitative real-time PCR (Q-PCR) validated the microarray data and additionally demonstrated that miR-17 upregulation correlates with disease progression in both models (Fig. 1d,e). To identify the precise location of dysregulated miR-17 expression, we performed *in situ* hybridization using a locked nucleic acid (LNA)-modified probe against the mature miR-17 transcript. miR-17 expression was increased in cyst epithelia of both *Pkd1*-mutant and *Pkd2*-KO kidneys (Fig. 1f). miR-17 expression was significantly reduced in cyst epithelia of *Pkd2*-miR-17∼92KO mice (negative control), whereas its expression was induced in renal tubules of kidney-specific miR-17∼92-overexpressing transgenic mice (positive control), indicating that the *in situ* probe specifically detects miR-17. The human mature miR-17 sequence is identical to the mouse miR-17. Therefore, we used this *in situ* probe to examine miR-17 expression in kidney samples from normal humans (NHK) and patients with ADPKD. Compared with renal tubules in NHK, miR-17 expression was increased in kidney cysts from patients with ADPKD (Fig. 1g). No signal was observed when *in situ* hybridization was performed using a probe with a scrambled sequence.

### miR-17∼92 promotes cyst growth in early-onset ADPKD models

**Figure 2:**
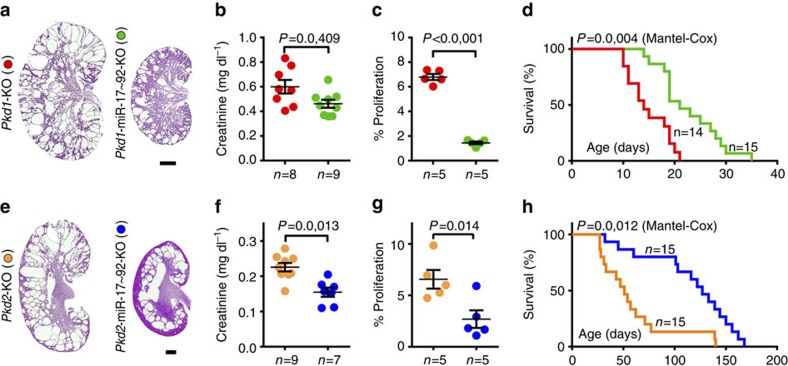
miR-17∼92 deletion attenuates cyst growth in early-onset ADPKD models. To understand the biological relevance of miR-17 upregulation, the miR-17∼92 cluster was deleted in orthologous ADPKD models. (**a**) H&E staining, (**b**) serum creatinine levels and (**c**) quantification of proliferating cyst epithelial cells in kidneys of 10-day-old *Pkd1*-KO and *Pkd1*-miR-17∼92KO mice is shown. (**d**) Kaplan-Meier survival curves of *Pkd1*-KO (red line) and *Pkd1*-miR-17∼92KO (green line) mice. The median survival of *Pkd1*-miR-17∼92KO (21-days) was improved by 50% compared with *Pkd1*-KO mice (14 days). (**e**) H&E staining, (**f**) serum creatinine levels and (**g**) quantification of proliferating cyst epithelial cells in kidneys of 21-day-old *Pkd2*-KO and *Pkd2*-miR-17∼92KO mice is shown. (**h**) Kaplan-Meier survival curves of *Pkd2*-KO (orange line) and *Pkd2*-miR-17∼92KO (blue line) mice. The median survival of *Pkd2*-miR-17∼92KO (127 days) was doubled compared with *Pkd2*-KO mice (51 days). Error bars represent s.e.m. Student's unpaired *t*-test (**b**,**c**,**f**,**g**) and Log rank (Mantel-Cox) test (**d**,**h**). Scale bars, 1 mm.

The miR-17 miRNA family aggravates cyst growth in the *Kif3a*-KO ciliopathy model of PKD^12^. To examine whether miR-17 plays a similar pathogenic role in ADPKD, miR-17∼92 was genetically deleted in various orthologous ADPKD mouse models. First, we deleted miR-17∼92 in *Pkd1*-KO mice, which develop an early-onset and rapidly fatal form of PKD. miR-17∼92^F/F^ mice were bred with Ksp/Cre;*Pkd1*^F/+^ transgenic mice. The first and second generation progeny were intercrossed to generate Ksp/Cre;*Pkd1*^F/F^ (*Pkd1*-KO) and Ksp/Cre;*Pkd1*^F/F^; miR-17∼92^F/F^ (*Pkd1*-miR-17∼92KO) mice. Q-PCR analysis showed that compared with control kidneys, *Pkd1* expression was equally reduced in both *Pkd1*-KO and *Pkd1*-miR-17∼92KO kidneys indicating a similar level of Cre/*loxP* recombination (Supplementary Fig. 1A). In contrast, compared with control kidneys, miR-17 expression was increased by 50.3% in *Pkd1-*KO kidneys, whereas its expression was reduced by 51.9% in *Pkd1*-miR-17∼92KO kidneys (Supplementary Fig. 1B). We observed a 50% improvement in median survival, 22% reduction in serum creatinine levels, 26.8% reduction in kidney-weight-to-body-weight ratio and decreased expression of kidney injury markers *Kim1* (down by 38.7%) and *Ngal* (down by 43.9%) in *Pkd1*-miR-17∼92KO compared with *Pkd1*-KO mice (Fig. 2a,b,d and Supplementary Fig. 1D,E). Moreover, the number of cyst epithelial cells expressing phospho-histone H3, a marker of proliferating cells, was reduced by 78.7% (Fig. 2c).

Next, we studied the role of miR-17∼92 in Pkhd1/Cre;*Pkd2*^F/F^ (*Pkd2*-KO) mice, which with a median survival of ∼70-days exhibit a relatively less aggressive PKD. *Pkd2*-KO and Pkhd1/Cre;*Pkd2*^F/F^; miR-17∼92^F/F^ (*Pkd2*-miR-17∼92KO) mice were generated using the strategy described earlier. Q-PCR and western blot analysis showed that compared with control kidneys, *Pkd2* expression was equally reduced in both *Pkd2*-KO and *Pkd1*-miR-17∼92KO kidneys indicating a similar level of Cre/*loxP* recombination (Supplementary Fig. 2A,B). In contrast, compared with control kidneys, miR-17 expression was increased by 45.4% in *Pkd2-*KO kidneys, whereas its expression was reduced by 46.5% in *Pkd2*-miR-17∼92KO kidneys (Supplementary Fig. 2C). Compared with *Pkd2*-KO mice, we observed a 149% increase in median survival, 31.8% improvement in serum creatinine levels, 15.8% reduction in cyst index, reduced *Kim1* (down by 37.9%) and *Ngal* (down by 39.6%) expression, and a 58.9% decrease in the number of proliferating cyst epithelial cells in *Pkd2*-miR-17∼92KO mice (Fig. 2e–h and Supplementary Fig. 2). Thus, deletion of miR-17∼92 inhibited cyst proliferation and disease progression in both *Pkd1*-KO and *Pkd2*-KO models.

### miR-17∼92 promotes cyst growth in long-lived ADPKD models

**Figure 3:**
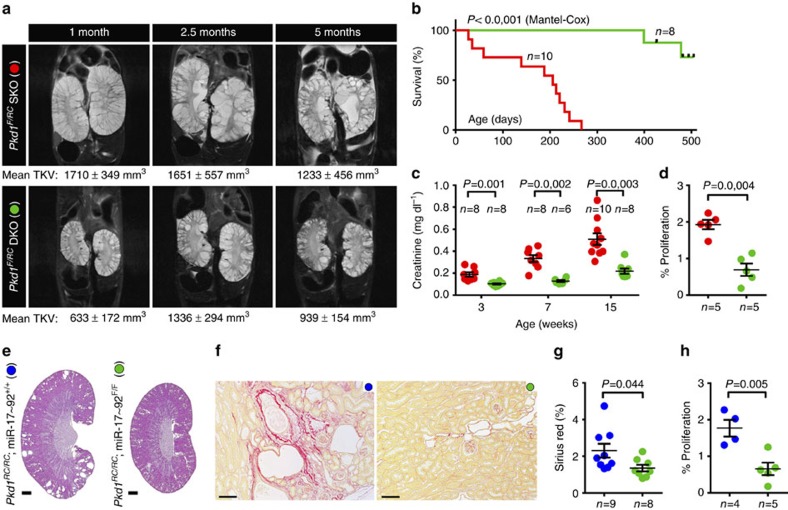
miR-17∼92 deletion attenuates disease progression in long-lived and slow cyst growth models of ADPKD. (**a**–**d**) The role of miR-17∼92 was studied in *Pkd1*^F/RC^SKO mice, a long-lived model of ADPKD. (**a**) Representative MRI images and mean MRI-estimated TKV of 1-,2.5-, and 5-month-old *Pkd1*^F/RC^SKO and *Pkd1*^F/RC^DKO mice are shown. TKV normalized to body weight was reduced in *Pkd1*^F/RC^DKO compared with *Pkd1*^F/RC^SKO mice at all time points (*n*=8–10 each group, *P*<0.01). (**b**) Kaplan-Meier survival curves of *Pkd1*^F/RC^SKO (red line) and *Pkd1*^F/RC^DKO mice (green line) are shown. The median survival (>14 months) of *Pkd1*^F/RC^DKO mice was more than doubled compared with *Pkd1*^F/RC^SKO mice (206 days). (**c**) Serum creatinine level was reduced in 3-,7-, and 15-week-old *Pkd1*^F/RC^DKO mice (green circles) compared with *Pkd1*^F/RC^SKO (red circles) mice. (**d**) Quantification revealed that cyst epithelial cell proliferation was reduced in 21-day-old *Pkd1*^F/RC^DKO mice compared with *Pkd1*^F/RC^SKO mice. (**e**–**h**) The role of miR-17∼92 was studied in *Pkd1*^RC/RC^ mice, a slow cyst growth model of ADPKD. (**e**) H&E and (**f**) Sirius red staining of kidney sections from 6-month-old *Pkd1*^RC/RC^ (blue circles) and *Pkd1*^RC/RC^;Ksp/cre; miR-17∼92^F/F^ (green circles) mice. Quantification of the Sirius red staining (**g**) and the number of proliferating cells (**h**) revealed that both interstitial fibrosis and tubular proliferation were reduced after miR-17∼92 deletion in *Pkd1*^RC/RC^ mice. Error bars represent s.e.m. Log rank (Mantel-Cox) test (**b**) Student's unpaired *t*-test (**a**,**c**,**d**,**g**,**h**). Scale bars, 1 mm (**e**) and 100 μm (**f**).

The majority of ADPKD patients do not experience a significant decline in renal function until the fourth or fifth decade of their life. However, *Pkd1*-KO mice develop a rapidly fatal form of PKD; thus, this model does not fully recapitulate the dynamics of human ADPKD progression. Therefore, we evaluated whether miR-17∼92 also influences disease progression in long-lived and slow cyst growth models of ADPKD. We generated *Pkd1*^F/RC^ mice that harbour a hypomorphic mutation^14^ (mouse equivalent of the human *PKD1*-R3277C (RC) mutation) on one allele and *loxP* sites flanking *Pkd1*-exons two and four on the other allele^15^. We used Ksp/Cre-mediated recombination to delete the floxed *Pkd1*-exons and thereby produced a compound *Pkd1*-mutant mouse with a germline hypomorphic mutation and a renal tubule-specific, somatic null mutation. To test the role of miR-17∼92 in this mouse model, we generated and characterized Ksp/Cre;*Pkd1*^F/RC^ (*Pkd1*^F/RC^SKO) and Ksp/Cre;*Pkd1*^F/RC^;miR-17∼92^F/F^ (*Pkd1*^F/RC^DKO) mice. *Pkd1* expression was unchanged whereas miR-17∼92 expression was reduced by 85.4% in *Pkd1*^F/RC^DKO compared with *Pkd1*^F/RC^SKO kidneys (Supplementary Fig. 3A,B). We prospectively monitored these mice for 5 months by performing kidney MRI and measuring serum creatinine levels at periodic intervals. MRI-assessed total kidney volume (TKV) was reduced, and the renal function was markedly improved in *Pkd1*^F/RC^DKO compared with *Pkd1*^F/RC^SKO mice throughout the observation period, indicating that deletion of miR-17∼92 provided a sustained clinical benefit (Fig. 3a,c). All *Pkd1*^F/RC^DKO mice lived well past 1 year, whereas the median survival of *Pkd1*^F/RC^SKO mice was only 206 days (Fig. 3b). We euthanize a separate group of 3-week-old and 5-month-old mice for molecular and histological analysis. Kidney-weights, cyst size, *Kim1* and *Ngal* expression, and the number of proliferating cyst epithelial cells were markedly reduced in *Pkd1*^F/RC^DKO compared with *Pkd1*^F/RC^SKO mice (Supplementary Fig. 3C–H).

Finally, we examined the role of miR-17∼92 in a slow cyst growth model of ADPKD (*Pkd1*^RC/RC^) that harbours homozygous germline *Pkd1* RC mutations. Unlike the preceding three ADPKD models, Cre/*loxP* recombination is not required to induce *Pkd1* mutation, and these mice develop significant renal fibrosis. We generated and characterized 6-month-old *Pkd1*^RC/RC^; Ksp/Cre and *Pkd1*^RC/RC^; Ksp/Cre;miR-17∼92^F/F^ mice. We observed attenuated cyst growth, lower blood urea nitrogen (BUN) levels, downregulation of *Kim1* and *Ngal* expression, reduced renal fibrosis and lower cyst epithelial proliferation in *Pkd1*^RC/RC^; Ksp/Cre;miR-17∼92^F/F^ compared with *Pkd1*^RC/RC^; Ksp/Cre mice (Fig. 3e–h and Supplementary Fig. 4).

### Anti-miR-17 attenuates cyst growth in two PKD models

Next, we studied whether miRNAs are viable drug targets for ADPKD. Within the miR-17∼92 cluster, we decided to target the miR-17 family based on our observation that multiple members of this family were upregulated in ADPKD models. We have recently developed a chemically modified anti-miR oligonucleotide (anti-miR-17) that sterically inhibits the activity of all miR-17 family members in cultured cells via complementary base pairing^16^. We used the miRNA polysome shift assay (miPSA) to assess whether this compound inhibits endogenous miR-17 in kidneys following systemic administration. miPSA relies on the principle that an active miRNA binds to its mRNA targets in translationally active high molecular weight polysomes. Compared with vehicle, a single subcutaneous injection of anti-miR-17 displaced miR-17 from high molecular weight polysomes in the kidney in a dose-dependent manner. Similar miR-17 displacement was not observed in mice treated with a control oligonucleotide (Supplementary Fig. 5B and Supplementary Table 3). Moreover, a 300 mg kg^−1^ dose of anti-miR-17 did not produce acute liver or kidney toxicity in mice (Supplementary Fig. 5C).

**Figure 4:**
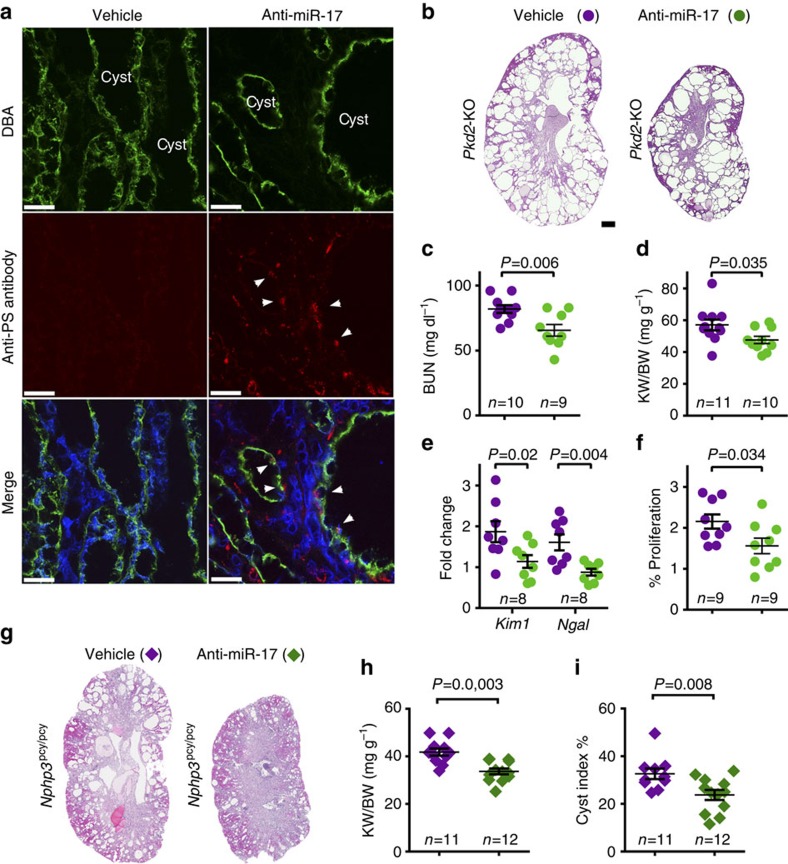
Anti-miR-17 demonstrates therapeutic efficacy in short-term and long-term PKD mouse models. (**a**) *Pkd2*-KO mice were injected with 20 mg kg^−1^ of anti-miR-17 compound or PBS on postnatal day (P) 21, 22 and 23, and kidneys were harvested on P26. Kidney sections were co-stained with DBA (green, a marker of collecting ducts) and anti-PS antibody (red, antibody labels anti-miR-17 compound). Anti-PS staining was observed in collecting duct-derived cysts (arrowheads) of *Pkd2*-KO mice injected with anti-miR-17 indicating that the compound was delivered to collecting duct cysts. No anti-PS antibody staining was noted in *Pkd2*-KO mice injected with PBS. To assess the therapeutic efficacy of this compound, *Pkd2*-KO mice were injected with anti-miR-17 or PBS at P10, 11, 12 and 19, and kidneys were harvested on P28. (**b**) H&E staining of kidney sections from 28-day-old *Pkd2*-KO mice injected with anti-miR-17 or PBS. (**c**) Serum BUN levels, (**d**) kidney-weight-to-body-weight ratio, (**e**) expression of *Kim1* and *Ngal* and (**f**) the number of proliferating cyst epithelial cells were reduced in *Pkd2*-KO mice that were injected with anti-miR-17 compared with PBS. (**g**–**i**) To assess the therapeutic efficacy of anti-miR-17 in a long-term PKD model, 1-month-old *Nphp3*^pcy/pcy^ mice were injected with 50 mg kg^−1^ of anti-miR-17 or PBS once a week for 26 weeks. These mice were euthanized at 30 weeks of age. (**g**) Representative images of H&E-stained kidney sections from 30-week-old *Nphp3*^pcy/pcy^ mice injected with PBS or anti-miR-17 are shown. (**h**) kidney-weight-to-body-weight ratios and (**i**) cyst index were reduced in *Nphp3*^pcy/pcy^ mice that were injected with anti-miR-17 compared with PBS. Error bars represent s.e.m. Student's unpaired *t*-test (**c**–**f**,**h**,**i**). Scale bars, 25 μm (**a**) and 1 mm (**b**).

To evaluate the delivery and therapeutic efficacy of anti-miR-17, we conducted randomized, blinded and statistically powered pre-clinical studies in *Pkd2-*KO mice (Supplementary Fig. 5D,E). We developed an antibody (anti-PS) that specifically binds to the chemically modified phosphate backbone of anti-miR-17 to determine its cellular distribution. Staining with the anti-PS antibody revealed that anti-miR-17 was delivered to collecting duct cysts even when administered after numerous cysts had already formed (Fig. 4a). No anti-PS signal was noted in kidneys of *Pkd2*-KO mice treated with PBS. To assess therapeutic efficacy, we injected *Pkd2*-KO mice with 20 mg kg^−1^ of anti-miR-17 or vehicle at postnatal days (P) 10, 11, 12 and 19 and killed them at P28. Kidney-weight-to-body-weight ratios, BUN levels and *Kim1* and *Ngal* expression were reduced in anti-miR-17-treated compared with vehicle-treated *Pkd2*-KO mice (Fig. 4b–e). Moreover, similar to genetic deletion, treatment with anti-miR-17 also slowed the proliferation of cyst epithelial cells (Fig. 4f). Next, we evaluated whether anti-miR-17 treatment demonstrates therapeutic efficacy in a second, more long-term model of cystic kidney disease. One-month-old *Nphp3*^pcy/pcy^ mice, a well characterized orthologous slow cyst growth model, were injected with 50 mg kg^−1^ of anti-miR-17 or vehicle once a week for 6 months (Supplementary Fig. 5F). We found that kidney-weight-to-body-weight ratio and cyst index were significantly reduced in anti-miR-17-treated compared with vehicle-treated *Nphp3*^*pcy/pcy*^ mice (Fig. 4g–i). Moreover, we did not observe any major adverse effects of long-term anti-miR-17 therapy such as weight loss, failure to thrive or death.

### Anti-miR-17 inhibits cysts in *in vitro* human models of PKD

**Figure 5:**
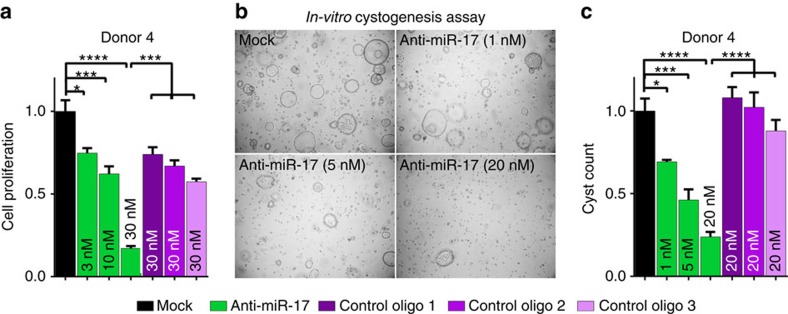
Anti-miR-17 treatment reduces proliferation and cyst growth in *in vitro* models of human ADPKD. Primary cyst epithelial cultures derived from kidneys of ADPKD patients were transfected with anti-miR-17 (dose: 3 nM, 10 nM or 30 nM) or three different control oligonucleotides (dose: 30 nM, control oligo1, 2 and 3). These cells were then cultured to measure proliferation or *in vitro* cyst formation (3-Dimension Matrigel). (**a**) Data for proliferation (*n*=5 for each treatment and dose) and (**b**) representative images and (**c**) quantification of cyst formation (*n*=3 for each treatment and dose) using primary cultures derived from ADPKD Donor 4 are shown. Anti-miR-17 reduced proliferation and cyst count in a dose-dependent manner. Data from the other ADPKD Donors can be found in the Supplementary Fig. 6. Error bars represent s.e.m. *indicates *P*<0.05, **indicates *P*<0.01, ***indicates *P*<0.005 and ****indicates *P*<0.001. One-way ANOVA, Tukey's multiple comparisons test.

To assess the translational potential of our findings, we studied the effects of miR-17 family inhibition in primary cell cultures derived from human ADPKD cysts. These cells were cultured to measure proliferation or *in vitro* cyst formation in 3D Matrigel. Treatment with anti-miR-17 produced a dose-dependent reduction in the proliferation of cyst epithelia from five human donors (Fig. 5a and Supplementary Fig. 6A). In contrast, mock transfection or transfection with three different control oligonucleotides had no effect. Similarly, compared with mock or control oligonucleotide transfection, anti-miR-17 inhibited *in vitro* cyst growth of primary ADPKD cells in a dose-dependent manner (Fig. 5b,c and Supplementary Fig. 6B).

### c-Myc promotes miR-17∼92 expression in PKD

**Figure 6:**
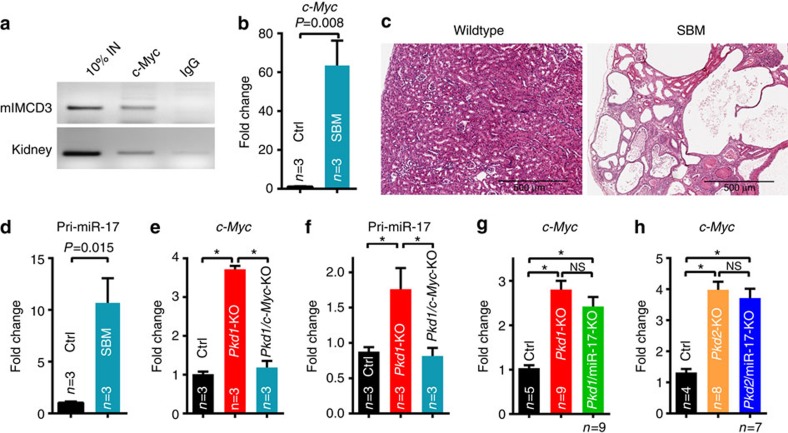
c-Myc promotes miR-17∼92 expression in cystic kidneys. (**a**) Chromatin immunoprecipitation was performed using an antibody against c-Myc and control IgG. Semi-qPCR analysis of the immunoprecipitated DNA revealed that c-Myc specifically binds to the miR-17∼92 promoter in mIMCD3 cells and mouse kidney tissue. No PCR product was observed in IgG control. To assess *in vivo* functional interaction, c-Myc transgenic (SBM) mice were analysed. (**b**) Q-PCR analysis showed *c-Myc* was upregulated and (**c**) H&E staining showed numerous kidney cysts in 6-month-old SBM compared with control mice. (**d**) Q-PCR analysis revealed that pri-miR-17 was increased by 12-fold in SBM compared with control kidneys (*n*=3), suggesting that c-Myc promotes miR-17 transcription in the context of PKD. To study whether c-Myc regulates miR-17∼92 expression in ADPKD models, Ksp/Cre; *Pkd1*^F/F^; *c-Myc*^F/F^ (*Pkd1*/*c-Myc*-KO) mice were generated. (**e**,**f**) Q-PCR analysis showing c-Myc and pri-miR-17 expression in 10-day-old control (Ctrl), *Pkd1*-KO and *Pkd1*/*c-Myc*-KO kidneys (*n*=3, each genotype). c-Myc deletion reduced pri-miR-17 expression in *Pkd1*-KO kidneys. (**g**,**h**) In contrast, Q-PCR analysis revealed that c-Myc expression was not affected by deletion of miR-17∼92 in *Pkd1* or *Pkd2*-KO mice. Thus, c-Myc functions upstream of miR-17∼92 in ADPKD models. Error bars represent s.e.m. *Indicates *P*<0.05. Student's unpaired *t*-test (**b**,**d**) One-way ANOVA, Tukey's multiple comparisons test (**e**–**h**). Scale bar 500 μm (**c**).

To gain insights into the signalling events linked to dysregulated miR-17∼92 expression, we began by identifying PKD-relevant upstream regulators of miR-17∼92. We focused on c-Myc because of its connection to both PKD pathogenesis and miR-17∼92 expression^17^,^18^,^19^. Chromatin immunoprecipitation analysis showed that c-Myc binds to the miR-17∼92 promoter in cultured renal epithelial cells and mouse kidneys (Fig. 6a). To study whether this interaction is functional in the context of PKD, we first analysed mice with a gain-of-function allele of c-Myc (SBM mice). Consistent with the previous report^19^, c-Myc was upregulated, and SBM mice exhibited numerous kidney cysts (Fig. 6b,c). Q-PCR analysis revealed that primary miR-17 transcript (pri-miR-17) was upregulated by 9.3-fold in SBM kidneys compared with control kidneys, suggesting that c-Myc drives miR-17∼92 transcription (Fig. 6d). To study whether this regulation is also observed in ADPKD models, we generated *Pkd1*-KO mice in which *c-Myc* was deleted (Fig. 6e). Deletion of *c-Myc* resulted in a 53.7% downregulation of pri-miR-17 expression, suggesting that c-Myc also promotes miR-17∼92 transcription in the context of ADPKD (Fig. 6f). In contrast, deletion of miR-17∼92 did not affect *c-Myc* expression in *Pkd1*-KO or *Pkd2*-KO kidneys, indicating that c-Myc functions upstream of miR-17∼92 in ADPKD (Fig. 6g,h).

### miR-17 modulates mitochondrial function by inhibiting Ppara

**Figure 7:**
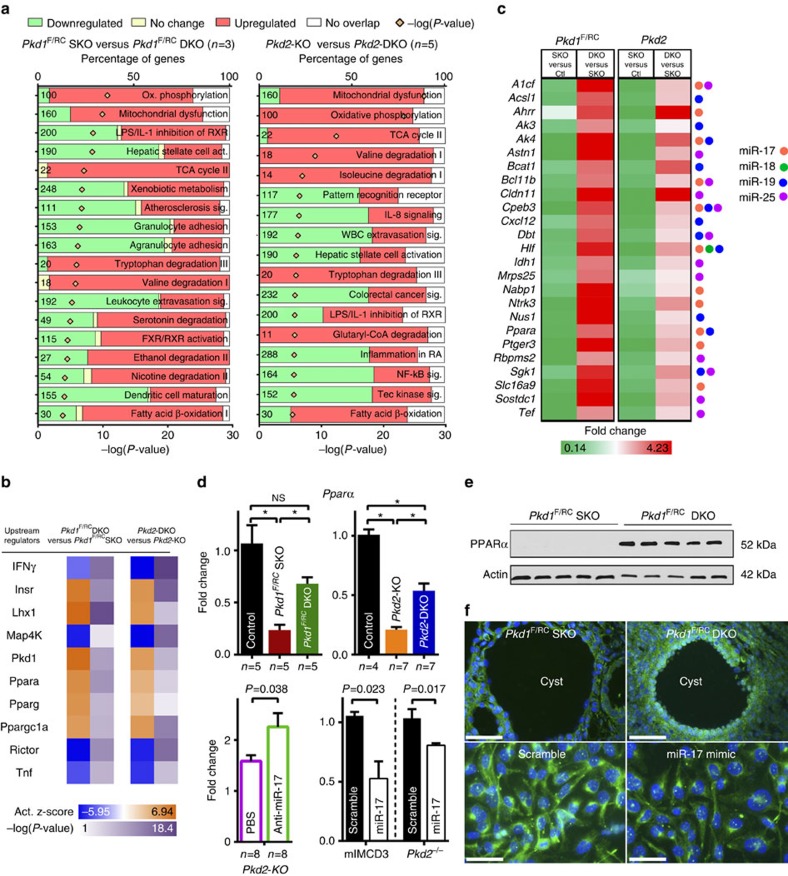
miR-17∼92 deletion results in improved expression of mitochondrial and metabolism-related gene networks. RNA-Seq analysis was performed to compare mRNA expression profiles. (**a**) Top differentially regulated pathways in 21-day-old *Pkd1*^F/RC^DKO compared with *Pkd1*^F/RC^SKO kidneys, and 21-day-old *Pkd2*-miR-17∼92KO compared with *Pkd2*-KO kidneys are shown. (**b**) Ingenuity pathway analysis software was used to identify the upstream regulators (URs) that may be responsible for the gene expression changes observed after miR-17∼92 deletion in ADPKD models. Top 10 (based on *z* scores) differentially expressed gene networks and their associated URs in *Pkd1*^F/RC^DKO compared with *Pkd1*^F/RC^SKO kidneys are shown. These networks were also differentially regulated in *Pkd2*-miR-17∼92KO compared with *Pkd2*-KO kidneys. Positive *z* scores (shades of orange) indicate activation whereas negative *z* scores (shades of blue) indicate inhibition of the gene networks. The *P* values (shades of purple) represent the level of statistical confidence for the prediction that the differentially expressed gene network is indeed regulated by the indicated UR. Large interconnected gene networks controlled by URs PPARα, PPARg and PPARGC1a were predicted to be activated after miR-17∼92 deletion in both ADPKD models. (**c**) RNA-Seq data were intersected with high-probability miR-17∼92 targets predicted by TargetScan. This analysis identified *Pparα* and 24 other common putative miR-17∼92 targets in the context of ADPKD. Expression of these genes was decreased (shades of green) in single knockout (SKO) compared with their respective control (Ctl) kidneys. In contrast, the expression of these genes was increased (shades of red) in double knockout (DKO) compared with their respective SKO kidneys. SKO indicates either *Pkd1*^F/RC^SKO or *Pkd2*-KO, whereas DKO indicates either *Pkd1*^F/RC^DKO or *Pkd2*-miR-17∼92KO kidneys. The circles indicate predicted binding sites for the various miRNA families derived from the miR-17∼92 cluster. (**d**) Q-PCR analysis showing *Pparα* expression in the indicated mouse models or cell lines. (**e**) Western blot showing increased PPARα expression in *Pkd1*^F/RC^DKO compared with *Pkd1*^F/RC^SKO kidneys. (**f**) PPARα antibody staining revealed that PPARα expression was increased in cyst epithelia of *Pkd1*^F/RC^DKO mice compared with *Pkd1*^F/RC^SKO mice. PPARα expression was decreased in mIMCD3 cells treated with miR-17 mimic compared with scramble mimic. Error bars indicate s.e.m. *indicates *P*<0.05, ns indicates *P*>0.05. One-way ANOVA, Tukey's multiple comparisons test, Student's unpaired *t*-test. Scale bars, 50 μm (**f**, top panel) and 20 μm (**f**, bottom panel).

To explore the downstream mechanisms, we performed RNA sequencing (RNA-Seq) analysis to compare mRNA expression profiles between kidneys of 21-day-old *Pkd1*^F/RC^SKO and *Pkd1*^F/RC^DKO mice (*n*=3 biological samples), and 21-day-old *Pkd2*-KO and *Pkd2*-miR-17∼92KO mice (*n*=5 biological samples). Pathway analysis suggested that the primary cellular consequence of miR-17∼92 deletion in both ADPKD models was improved mitochondrial metabolism (Fig. 7a and Supplementary Figs 7 and 8). Furthermore, upstream regulatory analysis revealed that gene networks controlled by key metabolism-related transcription factors *Pparα*, *Pparg* and *Ppargc1a* were activated upon miR-17∼92 deletion (Fig. 7b). Next, we intersected the RNA-Seq data from both ADPKD models with a list of high-probability direct mRNA targets of miR-17∼92. Intriguingly, this analysis identified *Pparα* as one of 25 common direct targets of miR-17∼92 in the context of ADPKD (Fig. 7c and Supplementary Tables 4,5).

Reprogrammed metabolism is thought to fuel cyst proliferation in ADPKD^20^,^21^. Based on the unbiased analysis of our RNA-Seq data, we reasoned that miR-17∼92-mediated direct repression of *Pparα* might provide one potential explanation for this phenomenon. First, we validated that *Pparα* is inhibited by miR-17∼92 in ADPKD models. Q-PCR analysis revealed that compared with control kidneys, *Pparα* expression was downregulated by ∼80% in *Pkd1*^F/RC^KO and *Pkd2*-KO kidneys, whereas its expression was upregulated in *Pkd1*^F/RC^DKO and *Pkd2*-miR-17∼92KO kidneys (Fig. 7d). *Pparα* expression was also increased in anti-miR-17-treated compared with vehicle-treated *Pkd2*-KO kidneys. Western blot analysis and immunostaining revealed that PPARα expression was markedly increased in cysts of *Pkd1*^F/RC^DKO kidneys compared with *Pkd1*^F/RC^SKO kidneys (Fig. 7e,f). Conversely, *Pparα* mRNA and protein expression was decreased in miR-17 mimic-treated compared with scramble mimic-treated mIMCD3 as well as *Pkd2*^−/−^ kidney epithelial cells (Fig. 7d,f).

**Figure 8:**
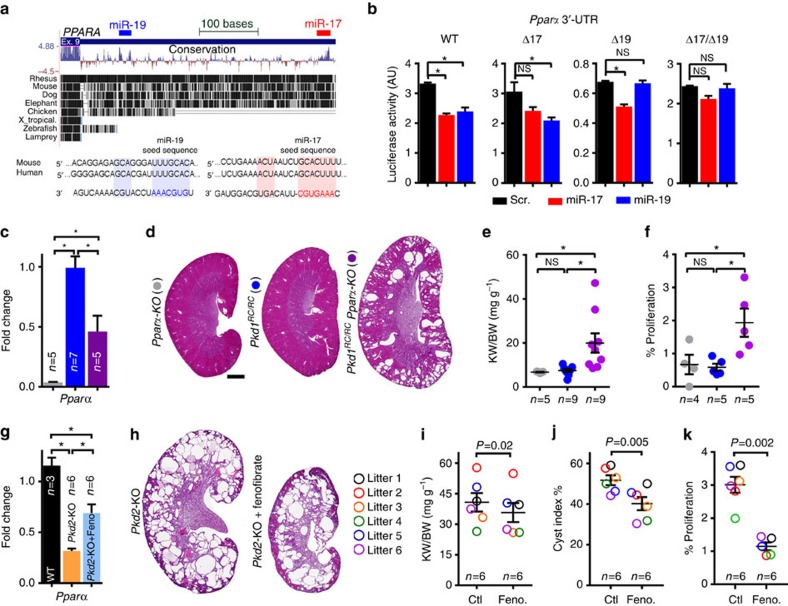
miR-17 aggravates cyst growth through direct repression of *Pparα*. (**a**) Human PPARA 3'-UTR *PPARΑ* 3′-UTR harbours evolutionarily conserved miR-17 (red box) and miR-19 (blue box) binding sites. Watson-Crick base-pairing between miR-17/*PPARΑ* 3′-UTR and miR-19/*PPARΑ* 3′-UTR is shown. (**b**) To test whether these binding sites are functional, mouse *Pparα* 3′-UTR was cloned into a luciferase reporter plasmid. mIMCD3 cells were co-transfected with this plasmid and scramble (scr, black), miR-17 mimic (red) or miR-19 mimic (blue) (*n*=3). Luciferase reporter assays revealed that compared with scramble, both miR-17 and miR-19 mimics suppressed wild-type *Pparα* 3′-UTR. Deleting the miR-17 binding site prevented miR-17-mediated, but not miR-19-mediated, repression. Similarly, deleting the miR-19 binding site abolished miR-19-mediated, but not miR-17-mediated, repression. Combined deletion of both binding sites abrogated repression by both miRNAs. (**c**–**f**) To test whether reduced *Pparα* gene dosage is sufficient to enhance proliferation and promote cyst formation, 6-week-old *Pparα*^−/−^ (*n*=5), *Pkd1*^RC/RC^ (*n*=9) and *Pkd1*^RC/RC^; *Pparα*-KO (*n*=9) mice were generated and characterized. (**c**) Q-PCR analysis showing *Pparα* expression in the indicated mouse models. (**d**) H&E staining, (**e**) kidney-weight-to-body-weight ratios and (**f**) quantification of proliferating epithelial cells in kidneys of *Pparα*^−/−^, *Pkd1*^RC/RC^ and *Pkd1*^RC/RC^; *Pparα*-KO mice is shown. (**g**–**k**) Conversely, to test whether increasing PPARα activity attenuates proliferation and cyst growth, 18-day-old *Pkd2*-KO littermates were fed a standard moist chow (Ctl) or a standard moist chow containing fenofibrate (Feno), a Pparα agonist, for 10 days. (**g**) Q-PCR analysis showed that *Pparα* expression was increased in *Pkd2*-KO mice fed Feno compared with Ctl. (**h**) H&E staining, (**i**) kidney-weight-to-body-weight ratios, (**j**) cyst index, and (**k**) quantification of proliferating cyst epithelial cells in *Pkd2*-KO mice fed Feno or Ctl is shown. Error bars indicate s.e.m. *indicates *P*<0.05, **indicates *P*<0.01, ***indicates *P*<0.001, and ns indicates *P*>0.05. One-way ANOVA, Tukey's multiple comparisons test (**b**–**g**), Student's unpaired *t*-test (**i**–**k**). Scale bars, 1 mm.

*Pparα* 3′-UTR harbours an evolutionarily conserved binding site for miR-17 and miR-19 families (Fig. 8a). To test whether the binding sites are functional, we co-transfected mIMCD3 cells with a luciferase reporter plasmid containing *Pparα* 3′-UTR and miR-17, miR-19, or scramble mimics (Fig. 8b). Both miR-17 and miR-19 repressed *Pparα* 3′-UTR. Deleting the miR-17 binding site prevented miR-17-mediated, but not miR-19-mediated, repression. Similarly, deleting the miR-19 binding site abolished miR-19-mediated, but not miR-17-mediated, repression. Combined deletion of both binding sites abrogated repression by both miRNAs.

Our results have shown that miR-17 and miR-19 directly inhibit *Pparα* expression in cystic kidneys, but whether reducing *Pparα* gene dosage is sufficient to promote cyst growth is not known. To address this question, *Pparα* was genetically deleted in the slow cyst growth *Pkd1*^RC/RC^ model. We characterized 6-week-old *Pparα*^−/−^ (*n*=5), *Pkd1*^RC/RC^ (*n*=9) and *Pkd1*^RC/RC^; *Pparα*^mut^ mice (*n*=6 *Pkd1*^RC/RC^; *Pparα*^+/−^ and *n*=3 *Pkd1*^RC/RC^; *Pparα*^−/−^). *Pparα*^−/−^ mice^22^ displayed normal kidney histology and *Pkd1*^RC/RC^ mice exhibited few (*n*=4 of 9) or no kidney cysts (*n*=5 of 9). In contrast, all *Pkd1*^RC/RC^; *Pparα*^mut^ mice displayed numerous cysts (*n*=9 of 9). Accordingly, kidney-weight-to-body-weight ratio, the expression of *Kim1* and *Ngal*, and proliferation were increased in *Pkd1*^RC/RC^; *Pparα*^mut^ mice compared with *Pparα*^−/−^ and *Pkd1*^RC/RC^ mice (Fig. 8c–e and Supplementary Fig. 11). Conversely, we tested whether increasing *Pparα* expression attenuates proliferation and cyst growth. First, using the *in vitro* MTT assay, we found that treatment with miR-17 mimic increased proliferation of cultured mIMCD3 and *Pkd2*^−/−^ cells, whereas genetic (*Pparα* plasmid) or pharmaceutical (WY14643) activation of PPARα abrogated miR-17-stimulated proliferation of mIMCD3 and *Pkd2*^−/−^ cells (Supplementary Fig. 12H). Next, to test the role of PPARα *in vivo*, *Pkd2*-KO mice were treated with fenofibrate, a PPARα agonist. Six littermate pairs of 18-day-old *Pkd2*-KO mice were placed on a diet of standard chow or standard chow supplemented with fenofibrate for 10 days. Fenofibrate treatment increased *Pparα* expression in *Pkd2*-KO kidneys (Fig. 8g), whereas it reduced kidney-weight-to-body-weight ratio, cyst index, and proliferation suggesting that increasing *Pparα* expression attenuates cyst growth (Fig. 8h–k).

**Figure 9:**
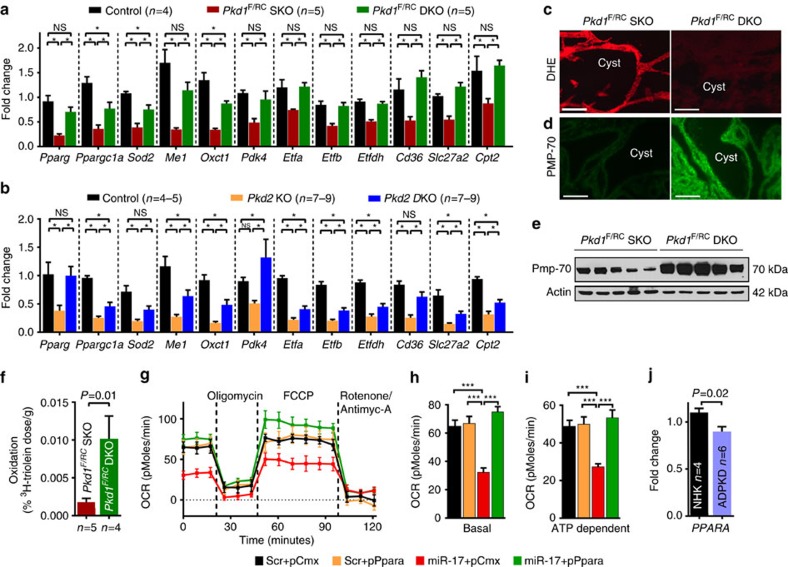
miR-17 modulates metabolic functions of PPARα. PPARα regulates a large gene network that modulates several aspects of metabolism including OXPHOS, FAO, and peroxisome function. We tested whether miR-17 affected these functions of PPARα. (**a**,**b**) Q-PCR analysis of metabolism-related PPARα target genes in the indicated mouse models. Expression of PPARα targets was reduced in ADPKD models, whereas their expression was increased after miR-17∼92 deletion. (**c**) To assess mitochondrial function *in vivo*, kidney sections were stained with dihydroethidium (DHE) to assess reactive oxygen species production. Reactive oxygen species level was markedly decreased in kidneys of 21-day-old *Pkd1*^F/RC^DKO mice compared with *Pkd1*^F/RC^SKO mice. (**d**) Kidney sections were stained using an antibody against PMP-70, an abundant and integral membrane protein of peroxisomes. PMP-70 expression was increased in kidney cysts of 21-day-old *Pkd1*^F/RC^DKO mice compared with *Pkd1*^F/RC^SKO mice. (**e**) Western blot analysis showing increased PMP-70 expression in kidneys of 21-day-old *Pkd1*^F/RC^DKO mice compared with *Pkd1*^F/RC^SKO mice. (**f**) To test whether miR-17∼92 deletion affected FAO, 21-day-old *Pkd1*^F/RC^SKO and *Pkd1*^F/RC^DKO mice were injected with a ^3^H-triolein tracer and kidney FAO was measured. Mirroring PPARα expression, FAO was increased in *Pkd1*^F/RC^DKO kidneys compared with *Pkd1*^F/RC^SKO kidneys. (**g**–**i**) To determine whether miR-17 affects OXPHOS, *Seahorse* XF 24 Analyzer was used to measure real-time mitochondrial OCR. (**g**) Real-time OCR tracings, (**h**,**i**) Basal and ATP-dependent OCR of *Pkd2*^−/−^ cells is shown (*n*=4). (**j**) *PPARα* expression was analysed in NHKs and human ADPKD cells. Q-PCR analysis showed that the expression of *PPARα* was decreased in cells derived from human ADPKD cysts compared with NHK. Error bars indicate s.e.m. *indicates *P*<0.05, ***indicates *P*<0.001, and ns indicates *P*>0.05. One-way ANOVA, Tukey's multiple comparison test (**a**,**b**,**h**,**i**), Student's unpaired *t*-test (**f**,**j**).

PPARα regulates several aspects of metabolism including oxidative phosphorylation (OXPHOS), fatty acid oxidation (FAO)^23^ and peroxisome function^10^. Therefore, we tested whether miR-17 affected these functions of PPARα. RNA-Seq (Supplementary Fig. 9,10) and subsequent Q-PCR analyses (Fig. 9a,b) showed that a large network of OXPHOS/FAO-related PPAR*α* target genes were upregulated after miR-17∼92 deletion in both ADPKD models. To quantitatively measure OXPHOS, we determined the real-time oxygen consumption rate (OCR) of mIMCD3 and *Pkd2*^−/−^ cells. Treatment with miR-17 mimics reduced basal and ATP-dependent OCR in mIMCD3 and *Pkd2*^−/−^ cells. Moreover, re-expression of *Pparα* normalized ATP-dependent OCR of miR-17 mimic-treated mIMCD3 and *Pkd2*^−/−^ cells (Fig. 9g–i and Supplementary Fig. 12). To assess mitochondrial metabolism *in vivo*, we injected *Pkd1*^F/RC^SKO and *Pkd1*^F/RC^-DKO mice with a ^3^H-triolein tracer and measured FAO^24^. Mirroring PPARα expression, FAO was increased in *Pkd1*^F/RC^DKO kidneys compared with *Pkd1*^F/RC^SKO kidneys (Fig. 9f). Reactive oxidative species level determined by dihydroethidium staining was reduced, whereas the number of peroxisomes assessed by the expression of PMP-70, an abundant and integral membrane protein of peroxisomes, was increased in *Pkd1*^F/RC^DKO compared with *Pkd1*^F/RC^SKO kidneys (Fig. 9c–e). Finally, since miR-17 is upregulated, we determined whether *PPARα* is downregulated in human ADPKD cysts. Q-PCR analysis revealed that *PPARα* expression was decreased in primary cells obtained from human ADPKD cysts compared with NHK (Fig. 9j). Consistent with these results, analysis of publicly available microarray data^25^ also showed that *PPARα* and its target genes are downregulated in human ADPKD cysts compared with normal renal cortical tissue (Supplementary Fig. 13). Collectively, these observations suggest that miR-17 promotes proliferation in cystic kidneys, at least in part, by reprogramming metabolism through direct repression of *Pparα*.

## Discussion

The pre-clinical studies presented here indicate that miR-17∼92 is a novel drug target for ADPKD. In support of this conclusion, we show that genetic deletion of miR-17∼92 attenuates disease progression in ADPKD mouse models irrespective of the mutated gene (*Pkd1* or *Pkd2*), the type of mutation (null or hypomorphic) or the dynamics of cyst growth (rapidly fatal, aggressive but long-lived or slowly progressing). In a complementary pharmaceutical approach, we demonstrate that anti-miR-17 also slowed cyst growth in two orthologous mouse models, including a long-lived, slow cyst growth model. Importantly, we show that anti-miRs can be delivered to collecting ducts following systemic administration, perhaps owing to the altered vasculature of cystic kidneys. This suggests that anti-miRs are a viable new drug class for ADPKD. These findings are likely to be relevant to human ADPKD pathogenesis because inhibiting miR-17 also attenuated proliferation and cyst growth of primary human ADPKD cultures. Since miR-17 inhibition slows cyst growth in ciliopathy models (*Kif3a*-KO and *Nphp3*^pcy/pcy^), the beneficial effects of anti-miR-17 treatment may also be observed in other forms of cystic kidney disease besides ADPKD. While our data demonstrated that administration of anti-miRs up to 6 months is feasible, additional pre-clinical studies are needed to fully address the long-term safety profile of anti-miR-17 therapy and to further explore miR-17 as a drug target for PKD.

**Figure 10:**
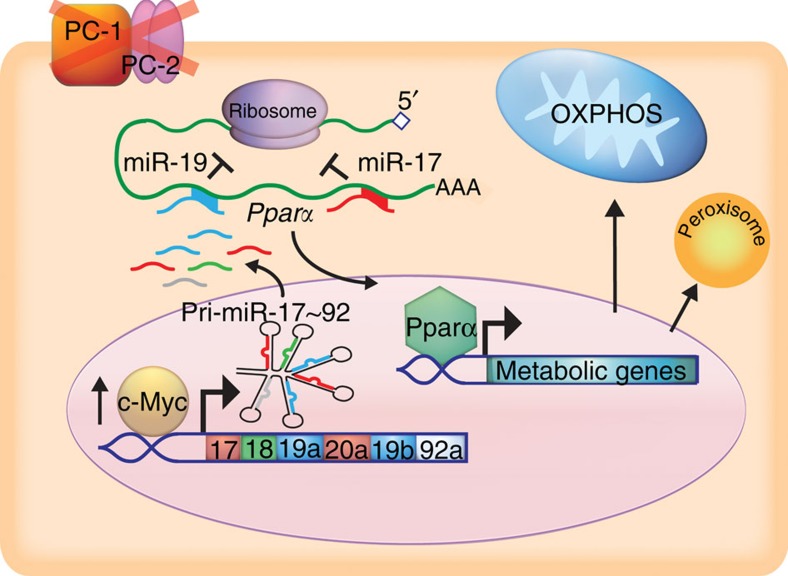
The proposed mechanism by which miR-17∼92 promotes ADPKD progression. Mutations of *Pkd1* or *Pkd2* is associated with increased expression of c-Myc. c-Myc binds to miR-17∼92 promoter and enhances its transcription in cystic kidneys. The miR-17∼92 primary transcript is processed to yield the individual mature miRNAs. In the cytoplasm, the mature miRNAs (miR-17 and miR-19) bind to *Pparα* 3′-UTR. PPARα is known to regulate the expression of key metabolic genes involved in mitochondrial OXPHOS pathway. miR-17 and miR-19 binding to *Pparα* 3′-UTR lead to reduced *Pparα* expression, which in turn affects mitochondrial metabolism in kidney epithelial cells.

Our studies point to pro-proliferative metabolic reprogramming induced by the c-Myc-miR-17-*Pparα* signalling axis as a potential new mechanism for PKD pathogenesis (Fig. 10). Quiescent kidney epithelial cells rely on FAO and the highly efficient OXPHOS pathway for ATP to maintain homoeostasis^23^. However, rather than ATP, a more critical limiting factor for proliferating cells is the amount of biosynthetic intermediates available for DNA replication and synthesis of new cell membranes and organelles^26^,^27^,^28^. Consistent with this notion and similar to cancer cells, cyst epithelia appear to depend on two alternative c-Myc-activated metabolic pathways of glycolysis and glutaminolysis to fuel their proliferation^20^,^21^,^29^. Our work suggests that an important additional component of this metabolic re-wiring is the inhibition of FAO and OXPHOS mediated, at least in part, by the miR-17-*Pparα* axis (Fig. 10). By reducing OXPHOS, carbon can be shuttled to alternate metabolic pathways to produce building blocks for the formation of new cells^30^. Indeed *Pkd1*-mutant renal epithelial cells exhibit reduced OXPHOS^31^. Moreover, *PPARΑ* and its target genes are among the top downregulated networks in murine and human ADPKD cysts^25^, further suggesting that reduced FAO and OXPHOS contributes to cyst pathogenesis. Importantly, mutations in FAO/OXPHOS and PPARA target genes cause clinical disorders that are often characterized by cystic kidneys^32^,^33^,^34^,^35^,^36^. For example, patients with mutations in *CPT2*, an enzyme that transports FA into mitochondria, or patients with Zellweger syndrome, a disorder caused due to defective peroxisomes, develop cystic kidneys. Glutaric acidemia type-II, caused by mutations of either *ETFA*, *ETFB* or *ETFDH* that collectively encode the OXPHOS enzyme electron transfer flavoprotein, is also characterized by PKD. Interestingly, *Etfa*, *Etfb*, *Etfdh*, *Cpt2* and the various peroxisome genes were all downregulated in both *Pkd1* and *Pkd2* mutant kidneys, whereas their expression was increased after miR-17∼92 deletion.

Another implication of our work is that agonists of the PPAR pathway may also have therapeutic value in PKD. Several previous studies have shown that treatment with PPARγ agonists (pioglitazone or rosiglitazone) reduces proliferation and retards cyst growth in rodent PKD models^37^,^38^,^39^,^40^. We observed a similar beneficial effect of PPARα agonist (fenofibrate) treatment in *Pkd2*-KO mice. However, a potential limitation of these widely used PPAR pathway drugs is that they cause kidney-related side effects. Pioglitazone can cause fluid retention and exacerbate cardiovascular symptoms. Accordingly, whether lower-dose pioglitazone treatment can be safely tolerated by ADPKD patients is currently being evaluated in a clinical trial. Fenofibrate use is associated with an elevation in serum creatinine and BUN levels^41^; therefore, whether this drug can be used over the long term in kidney disease patients is unclear.

In conclusion, miR-17∼92 promotes ADPKD progression through a new mechanism involving the inhibition of mitochondrial function. Importantly, miR-17 is a feasible and novel drug target for ADPKD. Our pre-clinical work provides a strong rationale for the development of a miRNA-based therapeutic approach for ADPKD.

## Methods

### Mice

Ksp/Cre^42^, Pkhd1/Cre^43^, *Pkd1*^F/F^^15^, *Pkd2*^F/F^^15^, miR-17∼92^F/F^ (ref. 44), miR-17 transgenic mice^45^, *Pkd1*^RC/RC^ (ref. 14) and c-Myc transgenic (SBM)^46^ and *Pparα*^−/−^22 mice were used in this study. The generation of the following mouse lines is discussed in the results section: *Pkd1*-miR-17∼92KO, *Pkd2*-miR-17∼92KO, Ksp/Cre;*Pkd1*^F/RC^ (*Pkd1*^F/RC^SKO), Ksp/Cre;*Pkd1*^F/RC^; miR-17∼92 ^F/F^ (*Pkd1*^F/RC^DKO), *Pkd1*^RC/RC^; Ksp/Cre, *Pkd1*^RC/RC^;Ksp/Cre;miR-17∼92^F/F^ and *Pkd1*/c-Myc-KO. For all studies, including the anti-miR studies, an equal number of male and female mice were used. The mice listed above were maintained in the B6 genetic background and their age(s) are indicated in the results section or the figure legends. We have previously used *Pkd2*-KO mice to analyse miR-17 expression by Q-PCR^12^. However, all analyses reported in the current paper was performed using new mice specifically generated for this project. All experiments involving animals were conducted under the approval of the UT Southwestern and Institut de Recherches Cliniques de Montréal Institutional Animal Care and Use Committee.

### Anti-miR-17 studies

Anti-miR-17 studies involving Pkhd1/Cre;*Pkd2*^F/F^ (*Pkd2*-KO) mice were utilized to determine the delivery and efficacy of the anti-miR-17 compound. Mice were randomly assigned to the treatment groups. All investigators were blinded to the treatment groups until predetermined analysis was complete. Based on our previous experience, power analysis (alpha <5% and power >80%) was performed *a priori* to determine the sample size (N of at least 8). *Nphp3*^pcy/pcy^ mice (CD-1 background) were obtained from PreClinOmics (Indianapolis, IN). Mutant mice were randomly assigned to the treatment groups. The investigators were not blinded to the treatment groups. Animal experiments were conducted in accordance with Institutional Animal Care and Use Committee guidelines via Explora BioLab services. The experimental approach for anti-miR-17 studies is shown in Supplementary Fig. 5.

### Fenofibrate studies

Littermate pairs of Pkhd1/Cre;*Pkd2*^F/F^ (*Pkd2*-KO) mice were administered either standard moist chow or standard moist chow supplemented with fenofibrate at a dose of 800 mg per day per kg body-weight for 10 days starting at postnatal day 18. The calculated dose is based on a food consumption rate of 160 mg diet per day per kg body-weight.

### Tissue harvesting and analysis

Mice were anesthetized under approved protocols, blood was obtained by cardiac puncture, and the right kidney was flash frozen for molecular analysis. The left kidney was perfused with cold PBS and 4% (wt/vol) paraformaldehyde and then collected. Kidneys were fixed with 4% paraformaldehyde for 2 h and then embedded in paraffin for sectioning. Sagittal sections of kidneys were stained with hematoxylin and eosin (H&E) or Pico Sirius red for additional analysis. Cyst index (cystic area/total kidney section surface area) and fibrosis area calculations (Pico Sirius red positive/total kidney section surface area) were performed using ImageJ analysis software.

### Renal and liver function tests

Serum creatinine was measured using capillary electrophoresis and BUN, aspartate aminotransferase, and alanine aminotransferase were measured using the Vitros 250 Analyzer.

### Human specimens

Frozen ADPKD and NHK specimens were provided by the PKD Research Biomaterials and Cellular Models Core at the Kansas University Medical Center (KUMC). ADPKD kidneys were obtained with the assistance of the KUMC Bio-Specimen Repository Core. Kidneys were immediately sealed in sterile bags, submerged in ice and delivered to the laboratory. Normal kidneys unsuitable for transplantation were obtained from the Midwest Transplant Network (Kansas City, KS). Informed consent was obtained. The protocol for the use of surgically discarded kidney tissues complied with federal regulations and was approved by the Institutional Review Board at the KUMC.

### MRI imaging

MRI scans were performed using a 7T small animal MRI scanner (Agilent (Varian), Inc, Palo Alto, CA) equipped with a 40 mm Millipede RF coil (ExtendMR LLC, Milpitas, CA). Under anaesthesia by inhalation of 1–3% isoflurane mixed in with medical-grade oxygen via nose-cone, the animals were placed supine with the respiratory sensor, head first with the kidneys centred with respect to the centre of a RF coil. MRI acquisitions were gated using the respiratory triggering. The bore temperature was kept at 28±1 °C. Two-dimensional (2D) scout images on three orthogonal planes (axial, coronal and sagittal) were acquired to ensure the positioning. For kidney volume measurements, the high-resolution T_2_-weighted fast spin-echo images were acquired on the coronal sections by applying a 6 ms sinc pre-saturation pulse to remove the fat signal. Some of the major imaging parameters were TR/TE=2,500/60 ms, FOV=32 × 32 mm, matrix size=256 × 256 (affording 125 μm in-plane resolution), slice thickness=1 mm, slice number=9–15 (dependent on the kidney size), no gap and number of average=6.

### RNA isolation and quantitative RT-PCR (Q-PCR)

Total RNA was isolated from cultured cells or mouse kidneys using miRNeasy Mini kits (Qiagen). First-strand cDNA was synthesized from mRNA using the iScript cDNA synthesis kit (Bio-Rad), and Q-PCR was performed using the iQ SYBR Green Supermix (Bio-Rad). The Universal cDNA Synthesis kit from Exiqon was used for first-strand synthesis from miRNA. Q-PCR was performed by using miRNA-specific forward and reverse LNA-enhanced PCR primers from Exiqon. To measure primary miRNA (pri-miRNA) expression, first-strand cDNA was synthesized by using the iScript cDNA synthesis kit (Bio-Rad). Q-PCR was performed by using the TaqMan Gene Expression Master Mix (Life Technologies) and pre-designed pri-miR-17∼92 primers from Life Technologies. The samples were loaded in triplicate on a CFX ConnectTM Real-time PCR detection system. 18S and 5S RNA were used to normalize expression of mRNA and miRNA, respectively. Data were analysed using the Bio-Rad CFX software. The sequences of the PCR primers are shown in Supplementary Table 6.

### miRNA microarray analysis

A nCounter Gene Expression CodeSet was purchased from Nanostring Technologies. nCounter miRNA expression assays were performed using total kidney RNA from *Pkd1*-KO and *Pkd2*-KO and their respective age-matched littermate controls (*n*=3 biological replicates for all groups) according to the manufacturer's instructions.

### RNA-Seq analysis

Strand-specific RNA-Seq libraries were prepared using the TruSeq Stranded Total RNA LT Sample Prep Kit from Illumina (*n*=3–5 biological replicates for all groups). After quality check and quantification, libraries were sequenced at the University of Texas Southwestern McDermott Center using a Hiseq2500 Sequencer to generate 51 bp single-end reads. Before mapping, reads were trimmed to remove low-quality regions in the ends. Trimmed reads were mapped to the mouse genome (mm10) using TopHat version 2.0.12 with the UCSC iGenomes GTF file from Illumina^47^. Alignments with mapping quality <10 were discarded. Expression abundance estimation and differential expression gene identification were done using edgeR^48^ or Cuffdiff version 2.2.1 (ref. 49). Genes with a *P* value <0.05 were deemed significantly differentially expressed between the two conditions. The identified genes were further subjected to ingenuity pathway analysis to identify cellular pathways and regulatory networks.

### *In situ* hybridization

10 μm thick frozen kidney sections were immersed overnight in 10% neutral buffered formalin. The next day, sections were washed with PBS three times and then, treated with proteinase K for 10 min at 37 °C. Subsequently, miRCURY LNA-ISH FFPE protocol was followed. The tissues were incubated with miR-17 (cat # 38461-01, Exiqon) or scramble (cat # 99004-01, Exiqon) probes. A hybridization temperature of 55 °C was used for both mouse tissue and human samples. Anti-Digoxigenin-AP Fab fragment antibody was used at 1:400 dilution (cat # 11093274910, Roche), and AP substrate was reapplied after 1 h. Slides were counterstained with nuclear fast red and then, mounted with Eukitt mounting medium. Samples were allowed to settle overnight and then, examined using light microscopy.

### Immunofluorescence staining

The following antibodies and dilutions were used on paraffin-embedded sections for immunofluorescence staining: anti-PS (Regulus Therapeutics Inc, 1:1,000), anti-Pparα (Abcam ab8934, 1:200), anti-Pmp-70 (EMD Millipore ABT12, 1:200), anti-phosphohistone H3 (1:400, Sigma-Aldrich H0412). Secondary antibodies were conjugated to Alexa Fluor 488 or Alexa Fluor 594 (Molecular Probes, 1:400). Lectins used were Dolichos biflorus agglutinin (DBA; Vector Laboratories, 1:400) and Lotus tetragonolobus agglutinin (LTA; Vector Laboratories, 1:400). Tissue sections were stained as described^43^. TUNEL assay was performed using the Promega Dead End Tunel Fluorometric System Kit per the manufacturer's directions with the following modification: the proteinase K treatment time was extended to 15 min. Quantification of apoptosis and proliferation was performed by randomly selecting 10 × 20 magnification fields per sample and subsequently determining the percentage of positively stained cyst epithelial cells. The person performing the quantification was blinded to the genotype of kidney sections.

### Western blot analysis

Total protein was extracted from kidneys or mIMCD3 cells. A total of 20 μg of protein was loaded on a 4–15% SDS-polyacrylamide gel and the proteins were transferred to a nitrocellulose membrane. The membrane was blocked with 5% bovine serum albumin and probed overnight at 4 °C with anti-Polycystin-2 (YCC2, gift from Stefan Somlo, 1:6,000), anti-PPARα (1:1,000) or anti-PMP-70 (1:1,000) antibodies. Goat-anti-rabbit HRP-conjugated IgG was used as a secondary antibody, and the blot was developed using the SuperSignal West Dura Extended Duration substrate from Pierce. The protein bands were quantified using Quantity One imaging software from Bio-Rad. Uncropped western blot images are shown in Supplementary Fig. 14.

### ChIP experiments

ChIP assays were performed using the ChIP-IT High Sensitivity Kit (catalogue # 53040, Active Motif) or the EZ ChIP Kit (catalogue # 17–371,EMD Millipore) according to the manufacturer's protocol. Briefly, mIMCD3 cells or mouse kidney tissue were crosslinked with 1% formaldehyde for 15 min at room temperature, homogenized into a single-cell suspension and chromatin samples were extracted from the nuclei and sonicated. Immunoprecipitation was performed with 5 μg of rabbit anti-Myc (sc-764; Santa Cruz Biotechnology) and rabbit IgG (sc-2027; Santa Cruz Biotechnology) antibody as a negative control. Genomic DNA was purified and amplified using the following primer set: forward 5′-AGGGCTCGTGGTTCTTAGGT-3′ and reverse 5′-GAAAAAGGGCAACCAGGACT-3′. This primer set amplifies a c-Myc binding site, which is ∼483 bp upstream of the transcription start site of the mouse miR-17∼92 cluster. Enrichment of c-Myc binding to the mir-17 site was compared with the Input DNA (10%). The PCR product (145 bp) was visualized on a 2% agarose gel.

### Reagents

Dual-Luciferase Reporter Assay System (catalogue#1960) and CellTiter 96 AQ_ueous_ Non-Radioactive Cell Proliferation Assay (catalogue#G5421) was purchased from Promega. DMEM low glucose medium (catalogue # 11885-084), DNase I (catalogue # 18068-015), and Lipofectamine 2000 (catalogue # 11668-019) were purchased from Life Technologies. miRNeasy Mini kits (catalogue # 217004) were purchased from Qiagen. QuikChange II Site-Directed Mutagenesis Kit (catalogue # 200523) was purchased from Agilent Technologies. The following miRNA mimics were purchased from Dharmacon, Inc (Thermo Fischer Scientific Inc) - miR-17 (catalogue # C-310561-07-0005), miR-19a (catalogue # C-310563-05-0005) and negative control or Scrambled (catalogue # CN-001000-01-05).

### Cell culture

Mouse inner medullary collecting duct (mIMCD3) cells were obtained from ATCC and grown in DMEM low glucose medium supplemented with 10% FBS and maintained in 5% CO_2_ atmosphere at 37 °C. *Pkd2*^−/−^ cells were obtained from Steve Somlo and grown in DMEM:F-12 medium (catalogue # 10565-018,Gibco) supplemented with 2% foetal bovine serum, insulin (8.3 × 10^−7^ M), prostaglandin E1 (7.1 × 10^−8^ M), selenium (6.8 × 10^−9^ M), transferrin (6.2 × 10^−8^ M), triiodothyronine (2 × 10^−9^ m), dexamethasone (5.09 × 10^−8^ m) and recombinant γ-interferon (10 units/ ml^−1^, Sigma) at 33 °C^50^ Human primary ADPKD cells were grown in DMEM:F-12 medium (catalogue # 10565-018,Gibco) supplemented with 5% FBS, 5 μg ml^−1^ insulin, 5 μg ml^−1^ transferrin and 5 ng ml^−1^ sodium selenite (ITS)(catalogue # 17-838Z, Lonza)^50^.

### Proliferation assays

The following experimental protocol was used for the proliferation assays that were performed in Supplementary Fig. 12G,H. For PPPARα expression plasmid studies, mIMCD3 cells were grown in six-well dishes (2 × 10^5^ cells per well), and transfected with 0.6 μg of a *Pparα* expression plasmid (pPparα) or Control (pCMX), along with 5 nM of miR-17 or scrambled mimic (Scr). Two days after transfection, the cells were transferred to a 96-well plate and incubated with 100 μl of the medium. For WY14643 studies, mIMCD3 or *Pkd2*^−/−^ cells were grown at 37 °C, plated in six-well dishes (2 × 10^5^ cells per well), and transfected with 5 nM of miR-17 or scrambled mimic. The medium was supplemented with 10 μM of WY14643 (dissolved in 70% DMSO/30% saline) or vehicle (DMSO). Two days after transfection, the cells were transferred to a 96-well plate and incubated with 100 μl of medium supplemented with 10 μM of WY14643 or vehicle. The MTT assay (Promega Corp) was performed after 24 h on mIMCD3 and *Pkd2*^−/−^cells, according to the manufacturer's directions. The quantity of formazan product was measured by the amount of absorbance at 490 nm, which is directly proportional to the number of living cells in culture.

The following protocol was used for the proliferation assays that were performed in Fig. 5a,c and Supplementary Fig. 6. At 80% confluency, human ADPKD cells were trypsinized using 1:10 dilution of Trypsin (catalogue # 25-053-CI, Corning) in Ca^+2^ and Mg^+2^ free PBS. These cells were transfected with anti-miR-17 and control oligos using RNAiMAX (catalogue # 13778-150, Life Technologies) following the manufacturer's protocol at 2,500 cells per well density in a 96-well plate. Cell viability was measured using MTT assay (catalogue# G4000, Promega) on day 3 following the manufacturers' protocol.

### Cyst formation assay

Human primary ADPKD cells were grown to 80% confluency and trypsinized as described earlier. At day 1, cells were transfected with the oligo of interest using RNAiMAX in a six-well plate format. Twenty-four hour after transfection, cells were trypsinized to single-cell suspension, counted and plated in a 96-well plate (cat # 353072, Corning) at 4,000 cells per well density in 130 μl of media plus Matrigel (cat # 354234, Corning) in a 4:5 ratio. Upon matrigel solidification, complete growth media was added to the well. Media was replenished every 72 h until 8 days post plating when the cyst size and number was measured. Each well was inspected for cyst proliferation using an Olympus D8 light microscope. Images were recorded with an Olympus DP26 camera (Olympus Corporation) from 28 focal planes, 150 μm apart, down into the well (on the *z* axis) as twenty-eight 24-bit colour TIFF images at 2,448-by-1,920 pixels at 72 dpi. Each focal plane's image from each well was processed using a custom R script (R Core Team 2015 R: A language and environment for statistical computing. R Foundation for Statistical Computing, Vienna, Austria. (URL https://www.R-project.org/) that used the EBImage Bioconductor package^51^. This script detected cysts in an automated and reproducible manner and was applied to all matrigel assays in all donors. Briefly, each image was masked for artifacts, filtered through a high-pass Laplacian filter and segmented with an adaptive threshold. Detected objects in the segmented images were further processed by pixel dilation, hole filling and by pixel erosion. Size, radius and eccentricity statistics on each segmented object were collected. Objects were filtered out if they had a mean radius less than or equal to 15 pixels, or were of a mean object radius greater than 200 pixels, or a coefficient of variation of the radius of greater than 0.2, or if the eccentricity of the detected object was greater than 0.75. Because cysts were often larger than the distance between focal planes, it was important to avoid counting such cysts more than once. If a cyst object in one image fell within the same *x* and *y* coordinates of a neighbouring image (for example, one focal plane above on the *z* axis), then this was counted as the same cyst. All images in each well were processed sequentially in this manner on the *z* axis. Finally, each cyst's volume was estimated by multiplying the mean radius of the largest object by 4/3πr3, assuming each cyst is a sphere.

### miRNA polysome shift assay (miPSA)

C57BL/6 mice (Jackson Laboratories) were injected with a single subcutaneous dose of 0.3, 3 or 30 mg kg^−1^ of anti-miR-17, a control oligonucleotide or PBS. The kidneys were collected after 7 days, and the tissues were processed^16^. The relative level of miR-17 in the polysome containing fractions was quantified using the miRNA TaqMan assays (Life Technologies). *Let-7d* was used as the reference gene. The relative displacement of miR-17 normalized to let-7d was calculated between the treated (anti-miR-17) and the control (PBS or control oligonucleotide) samples using double delta Ct (ΔΔCt) method. The displacement values are reported in log_2_ scale where the positive values reflect the loss of miR-17 from the high molecular weight polysome fractions.

### 3′-UTR plasmids

1456, bp of the mouse Pparα 3′-UTR that includes the miR-17 and miR-19a conserved sites was PCR amplified using forward and reverse primers that introduced NheI and XhoI restriction sites at the 5′- and 3′- ends, respectively. The sequence of the forward primer was 5′-TAGAATGCTAGCGCCACTGTTCAGGGACCTC-3′, whereas that of the reverse primer was 5′-TAGAATCTCGAGAGGCATCTACCACCATGTCC-3′, where the underlined sequences are the NheI and XhoI sites, respectively. The 1456, bp PCR product was ligated in the pLightSwitch Empty (pLS) 3′-UTR plasmid, that was previously digested with NheI and XhoI restriction enzymes to generate the WT Pparα 3′-UTR construct. The seed sequences for the miR-17 and the miR-19 binding sites were mutated in the WT-Pparα 3′-UTR construct to produce the Pparα 3′-UTR (Δ17) and Pparα 3′-UTR (Δ19) constructs. The sequences of the primers used in site-directed mutagenesis reactions are as follows-

Pparα 3′-UTR (Δ17)

Forward primer – 5′-CTCTTAAATCCCTGAAAACTAATCTTAGGCAGTTAACCTTTGAAAACCTACAAGTCAAGG -3′ Reverse primer- 5′-CCTTGACTTGTAGGTTTTCAAAGGTTAACTGCCTAAGATTAGTTTTCAGGGATTTAAGAG-3′

Pparα 3′-UTR (Δ19)

Forward primer- 5′-CCATACAGGAGAGCAGGGACAGTAGTCGAGGGCCTCCCTCCTACGC-3′

Reverse primer- 5′-GCGTAGGAGGGAGGCCCTCGACTACTGTCCCTGCTCTCCTGTATGG-3′

The underlined sequences denote the respective mutated miRNA binding sites. Site-directed mutagenesis was performed using the QuikChange II kit according to the manufacturer's instructions. The PCR protocol was as follows—1cycle at 95 °C for 1 min, followed by 18 cycles of 95 °C for 50 s, 60 °C for 50 s, 68 °C for 6 min and 1 cycle of 68 °C as a final extension cycle. The presence of the desired mutations was verified by DNA sequencing.

### Luciferase assays

mIMCD3 cells were plated in six-well dishes (2 × 10^5^ cells per well) and transfected with 0.4 μg of pLS-Renilla-3′-UTR plasmids, and 10 nM of miR-17 or miR-19a mimic. Cells were transfected with 0.04 μg of the pGL3-Control plasmid (Promega Corp) encoding Photinus luciferase to control for differences in transfection efficiency. Lipofectamine 2000 (Invitrogen) was used as a transfection reagent. Forty-eight hours later, the cells were lysed in 250 μl of passive lysis buffer (Promega Corp), and 40 μl of the cell lysate was added to 96-well plates. Photinus and Renilla luciferase activities were measured by using the Dual-Luciferase Reporter Assay System (Promega Corp) according to the manufacturer's directions.

### ^3^H-triolein uptake and β-oxidation

Endogenous triolein clearance rates and β-oxidation rates in kidneys were performed^24^. Briefly, 21-day-old *Pkd1*^F/RC^SKO and *Pkd1*^F/RC^DKO were injected with ^3^H-triolein (2 μCi per mouse in 100 μl of 5% intralipid) after an 8-h fast. Twenty minutes later, mice were killed, blood samples were taken and kidneys were collected, weighed and frozen at −80 °C until processing. Lipids were extracted, and radioactivity content was quantified.

### Mitochondrial OCR experiments

Mitochondrial OCRs were determined using an XF24 Extracellular Flux Analyzer (Seahorse Bioscience, MA) following the manufacturers' protocols. A basal-oligomycin-carbonyl cyanide 4-(trifluoromethoxy) phenylhydrazone (FCCP)-antimycin-A/rotenone ‘BOFA' experiment was utilized for the assessment of mitochondrial function and respiration rates in mIMCD3 cells. *Pkd2*^−/−^ or mIMCD3 cells were grown in six-well dishes (2 × 10^5^ cells per well). *Pkd2*^−/−^ cells were transfected with 0.4 μg of a PPARα expression plasmid (pPpara) or Control (pCMX), along with 1 nM of miR-17 or scrambled mimic (Scr). mIMCD3 cells were transfected with 0.6 μg of pPpara or pCMX, along with 5 nM of miR-17 or Scr mimic. Forty-eight hour post-transfection, *Pkd2*^−/−^ or mIMCD3 cells (60,000 per well) were seeded into an XF24 cell culture microplate (Seahorse Bioscience). Cells were then equilibrated for 1 h at 37 °C (non-CO_2_) in XF Assay Medium (Modified DMEM, 0 mM Glucose; Seahorse Bioscience) (pH 7.4), supplemented with 1 mM sodium pyruvate, 1 mM L-glutamine, and 7 mM glucose. The XF24 plate was then transferred to a temperature-controlled (37 °C) Seahorse analyzer and subjected to a 10-min equilibration period and three assay cycles to measure the basal rate, comprising a 3-min mix, a 2-min wait and a 3-min measure period each. Compounds were then added by automatic pneumatic injection followed by assay cycles after each, comprising of 3-min mix, 2-min wait and a 3-min measure period. OCR measurements were obtained following sequential additions of oligomycin (1 μM final concentration), FCCP (1 μM) and antimycin-A/rotenone (10 μM/100 nM). OCR measurements were recorded at set interval time points. The ATP-dependent OCR was calculated by subtracting the OCR reading recorded after oligomycin treatment from OCR reading recorded at basal levels ((ATP-linked OCR)=(basal OCR) - (post-Oligomycin OCR)). All compounds and materials above were obtained from Sigma-Aldrich.

### Statistical analysis

Data are shown as the mean±s.e.m. Statistical analysis was performed using Student's *t-*test for pairwise comparisons or Analysis of variance (ANOVA) followed by Tukey's post hoc test for multiple comparisons. Mantel-Cox test was used to analyse differences in mice survival. *P*<0.05 was considered significant. Primary outcomes (survival or renal function) in all mouse studies are statistically powered to achieve an alpha error <5% and power of >80%.

### Data availability

The RNA-Seq has been deposited in the NCBI Gene Expression Omnibus repository under accession number GSE89764. Other data are available from the corresponding author upon request.

## Additional information

**How to cite this article:** Hajarnis, S. *et al*. microRNA-17 family promotes polycystic kidney disease progression through modulation of mitochondrial metabolism. *Nat. Commun.* **8**, 14395 doi: 10.1038/ncomms14395 (2017).

**Publisher's note:** Springer Nature remains neutral with regard to jurisdictional claims in published maps and institutional affiliations.

## Supplementary Material

Supplementary Information Supplementary figures, supplementary tables and supplementary references.
