# Supplementary material for: microRNA-17 family promotes polycystic kidney disease progression through modulation of mitochondrial metabolism
Source: Nat Commun. 2017 Feb 16;8:14395. doi: 10.1038/ncomms14395 (PMC5316862; doi:10.1038/ncomms14395)
Supplement: Supplementary Information — Supplementary figures, supplementary tables and supplementary references. [file ncomms14395-s1.pdf]

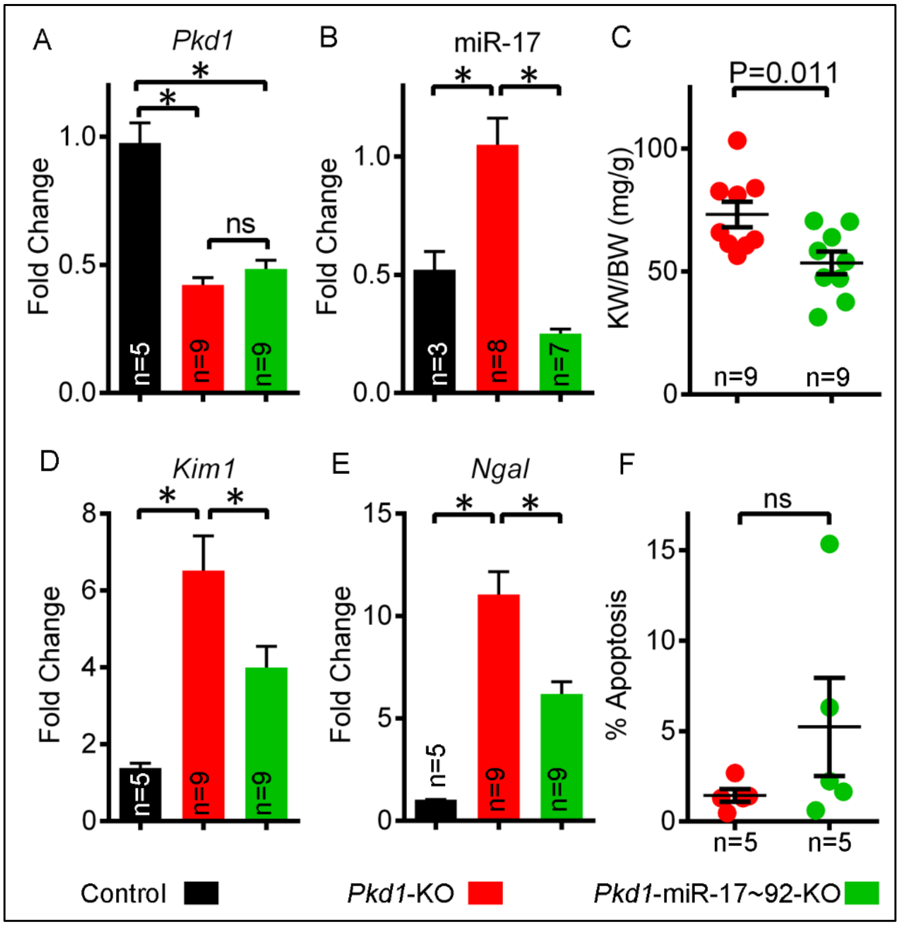

Supplementary Figure 1: To test the role of miR-17~92 in orthologous genetic model of ADPKD, we generated Ksp/Cre;*Pkd1*$^{F/F}$ (*Pkd1*-KO) and Ksp/Cre;*Pkd1*$^{F/F}$;miR-17~92$^{F/F}$ (*Pkd1*-miR-17~92KO) mice. (A) Q-PCR analysis revealed that compared to control kidneys (black), *Pkd1* expression was equally reduced in both *Pkd1*-KO (red) and *Pkd1*-miR-17~92KO (green) kidneys, indicating that a similar level of Cre/*loxP* recombination was observed. (B) Q-PCR analysis revealed that, compared to control kidneys, miR-17 expression was increased in *Pkd1*-KO kidneys, whereas its expression was reduced in *Pkd1*-miR-17~92KO mice. (C) Kidney-weight-to-body-weight (KW/BW) ratio and the expression of kidney injury markers (D) *Kim1* and (E) *Ngal* was reduced in *Pkd1*-miR-17~92KO compared to *Pkd1*-KO kidneys. (F) The number of cyst epithelia undergoing apoptosis did not change in *Pkd1*-miR-17~92KO compared to *Pkd1*-KO kidneys. Error bars indicate SEM. * indicates $P<0.05$, ns indicates $P>0.05$. One-way ANOVA, Tukey's multiple comparisons test (A, B, D, E), Student's unpaired *t*-test (C, F)

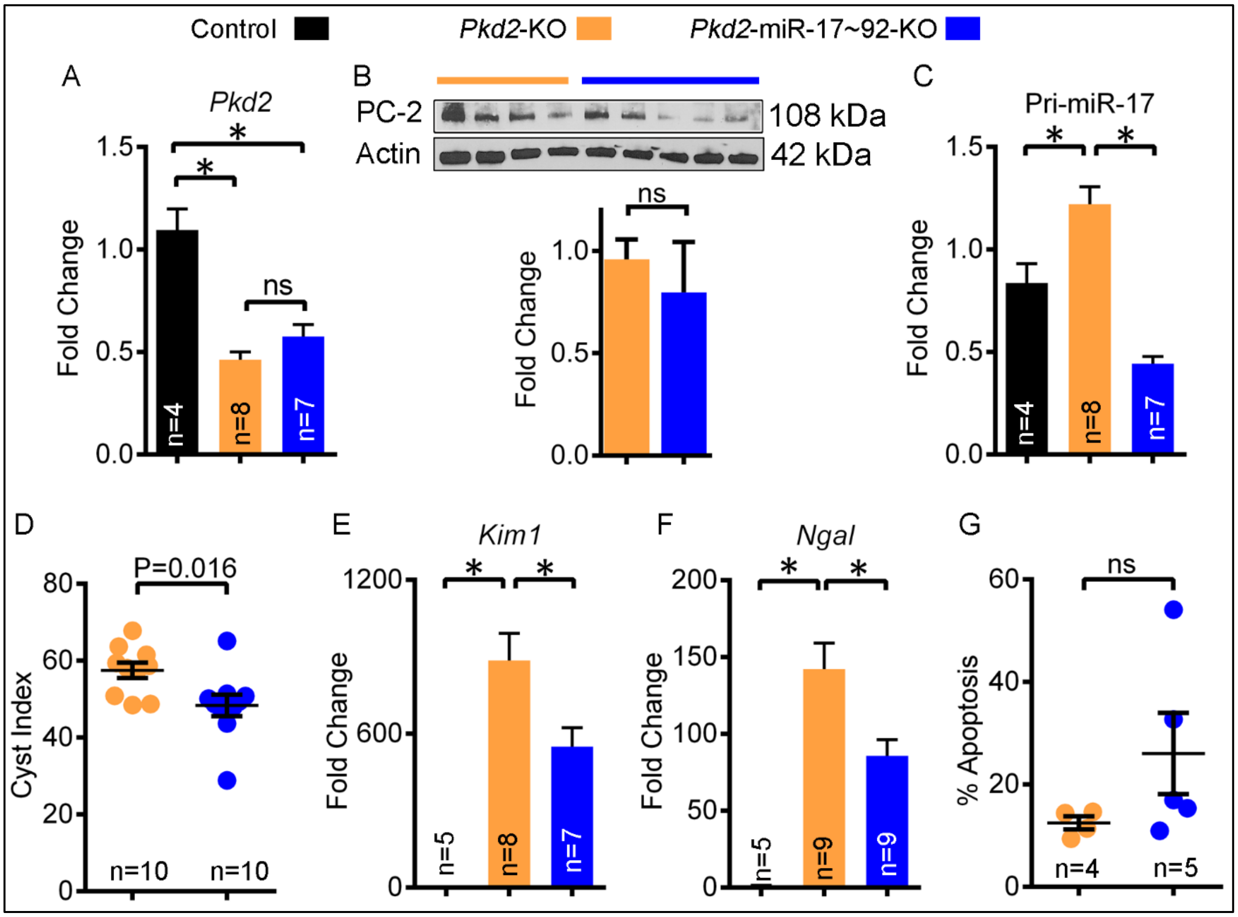

Supplementary Figure 2: To test the role of miR-17~92 in an orthologous genetic model of ADPKD bearing mutations in *Pkd2*, we generated Pkhd1/Cre;*Pkd2*$^{F/F}$ (*Pkd2*-KO) and Pkhd1/Cre;*Pkd2*;miR-17~92$^{F/F}$ (*Pkd2*-miR-17~92KO) mice. (A) Q-PCR analysis revealed that, compared to control kidneys (black), *Pkd2* expression was equally reduced in both *Pkd2*-KO (orange) and *Pkd2*-miR-17~92KO (blue) kidneys, indicating that a similar level of Cre/*loxP* recombination was observed. (B) Western Blot analysis and quantification of Polycystin-2 (PC-2) expression in *Pkd2*-KO and *Pkd2*-miR-17~92KO kidneys is shown. miR-17 deletion did not affect PC-2 expression in *Pkd2*-miR-17~92KO kidneys. (C) Q-PCR analysis revealed that, compared to control kidneys, miR-17 expression was increased in *Pkd2*-KO kidneys, whereas its expression was reduced by ~60% in *Pkd2*-miR-17~92KO mice. (D) Cyst index and the expression of kidney injury markers (E) *Kim1* and (F) *Ngal* was reduced in *Pkd2*-miR-17~92KO compared to *Pkd2*-KO kidneys. (G) The number of cyst epithelia undergoing apoptosis was higher in *Pkd2*-miR-17~92KO compared to *Pkd2*-KO kidneys. However, this difference was not statistically significant. Error bars indicate SEM. * indicates $P<0.05$, ns indicates $P>0.05$. . One-way ANOVA, Tukey's multiple comparisons test (A, C, E, F), Student's unpaired *t*-test (B, D, G)

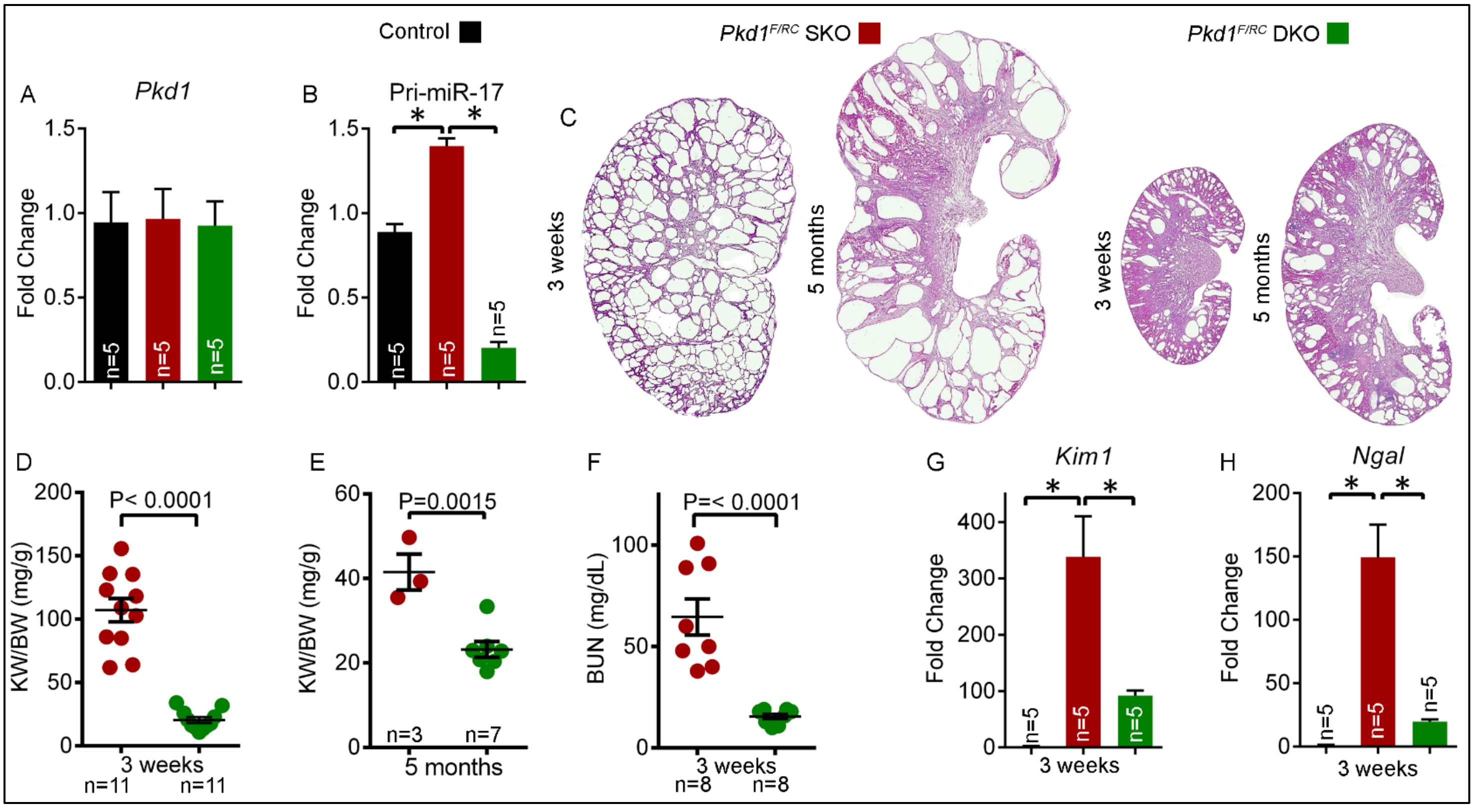

Supplementary Figure 3: To test the role of miR-17~92 in a long-lived ADPKD model, we generated Ksp/Cre;*Pkd1*$^{\mathrm{Flox/RC}}$ (*Pkd1*$^{\mathrm{F/RC}}$SKO) and Ksp/Cre;*Pkd1*$^{\mathrm{Flox/RC}}$;miR-17~92$^{\mathrm{F/F}}$ (*Pkd1*$^{\mathrm{F/RC}}$DKO) mice. This compound *Pkd1*-mutant mouse harbors a germline hypomorphic mutation on one allele and a Cre/*loxP* recombination-induced, renal tubule-specific, somatic null mutation on the other allele. (A) Q-PCR analysis revealed that *Pkd1* expression was not different between control (black), *Pkd1*$^{\mathrm{F/RC}}$SKO (red) and *Pkd1*$^{\mathrm{F/RC}}$DKO (green) kidneys, suggesting that deleting one floxed allele does not affect the total *Pkd1* mRNA expression. (B) In contrast, Q-PCR analysis revealed that, compared to control kidneys, miR-17 expression was increased in *Pkd1*$^{\mathrm{F/RC}}$SKO kidneys, whereas its expression was reduced by ~80% in *Pkd1*$^{\mathrm{F/RC}}$DKO kidneys. (C) H&E staining of kidney sections from three-week-old and five-month-old *Pkd1*$^{\mathrm{F/RC}}$SKO and *Pkd1*$^{\mathrm{F/RC}}$DKO mice is shown. (D&E) Kidney-weight-to-body-weight (KW/BW) ratio, (F) blood urea nitrogen (BUN) level and the expression of (G) *Kim1* and (H) *Ngal* was reduced in *Pkd1*$^{\mathrm{F/RC}}$DKO compared to *Pkd1*$^{\mathrm{F/RC}}$SKO mice. Thus, deletion of miR-17~92 markedly attenuates disease progression in Ksp/Cre;*Pkd1*$^{\mathrm{Flox/RC}}$ mice. Error bars indicate SEM. * indicates P<0.05, ns indicates P>0.05. One-way ANOVA, Tukey's multiple comparisons test (A, B, G, H), Student's unpaired *t*-test (D, E, F)

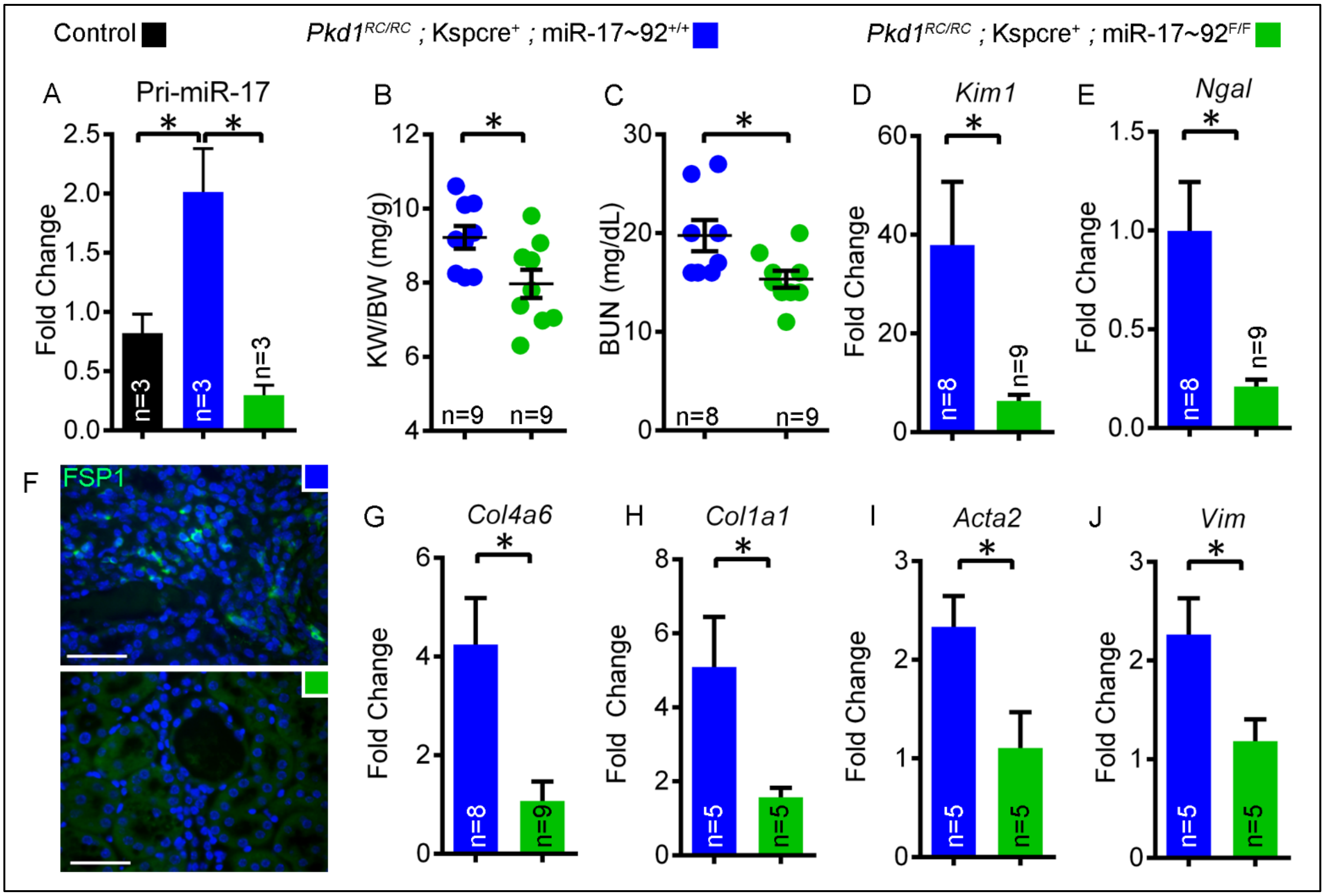

Supplementary Figure 4: To test the role of miR-17~92 in a slow cyst growth ADPKD model, we generated and characterized 6-month-old *Pkd1*$^{RC/RC}$; Ksp/Cre (*Pkd1*$^{RC/RC}$) and *Pkd1*$^{RC/RC}$; Ksp/Cre;miR-17~92$^{F/F}$ mice (*Pkd1*$^{RC/RC}$DKO). Unlike the preceding three ADPKD models, Cre/*loxP* recombination is not required to induce *Pkd1* mutation, and these mice develop significant renal fibrosis. Ksp/Cre was introduced into the cross to delete miR-17~92. (A) Q-PCR analysis revealed that, compared to control kidneys, miR-17 expression was increased in *Pkd1*$^{RC/RC}$ kidneys, whereas its expression was reduced in *Pkd1*$^{RC/RC}$DKO compared to *Pkd1*$^{RC/RC}$ mice. (B) Kidney-weight-to-body-weight ratio, (C) BUN and the expression of (D) *Kim1* and (E) *Ngal* was decreased in *Pkd1*$^{RC/RC}$DKO compared to *Pkd1*$^{RC/RC}$ mice. (F) Kidney sections were stained with an antibody against Fsp1 (fibroblast-specific protein 1). Fsp1 expression (green) was decreased in *Pkd1*$^{RC/RC}$DKO kidneys compared to *Pkd1*$^{RC/RC}$ kidneys. (G-J) Q-PCR analysis showed that the expression of other fibrosis markers (*Col4a6*, *Col1a1*, *Acta2,* and *Vimentin*) was also decreased in *Pkd1*$^{RC/RC}$DKO kidneys compared to *Pkd1*$^{RC/RC}$ kidneys. Collectively, these results indicate that miR-17~92 deletion attenuates disease progression in *Pkd1*$^{RC/RC}$ mice. Error bars indicate SEM. * indicates $P<0.05$, ns indicates $P>0.05$. One-way ANOVA, Tukey's multiple comparisons test (A) Student's unpaired *t*-test (B-J). Scale Bar: 50 µm.

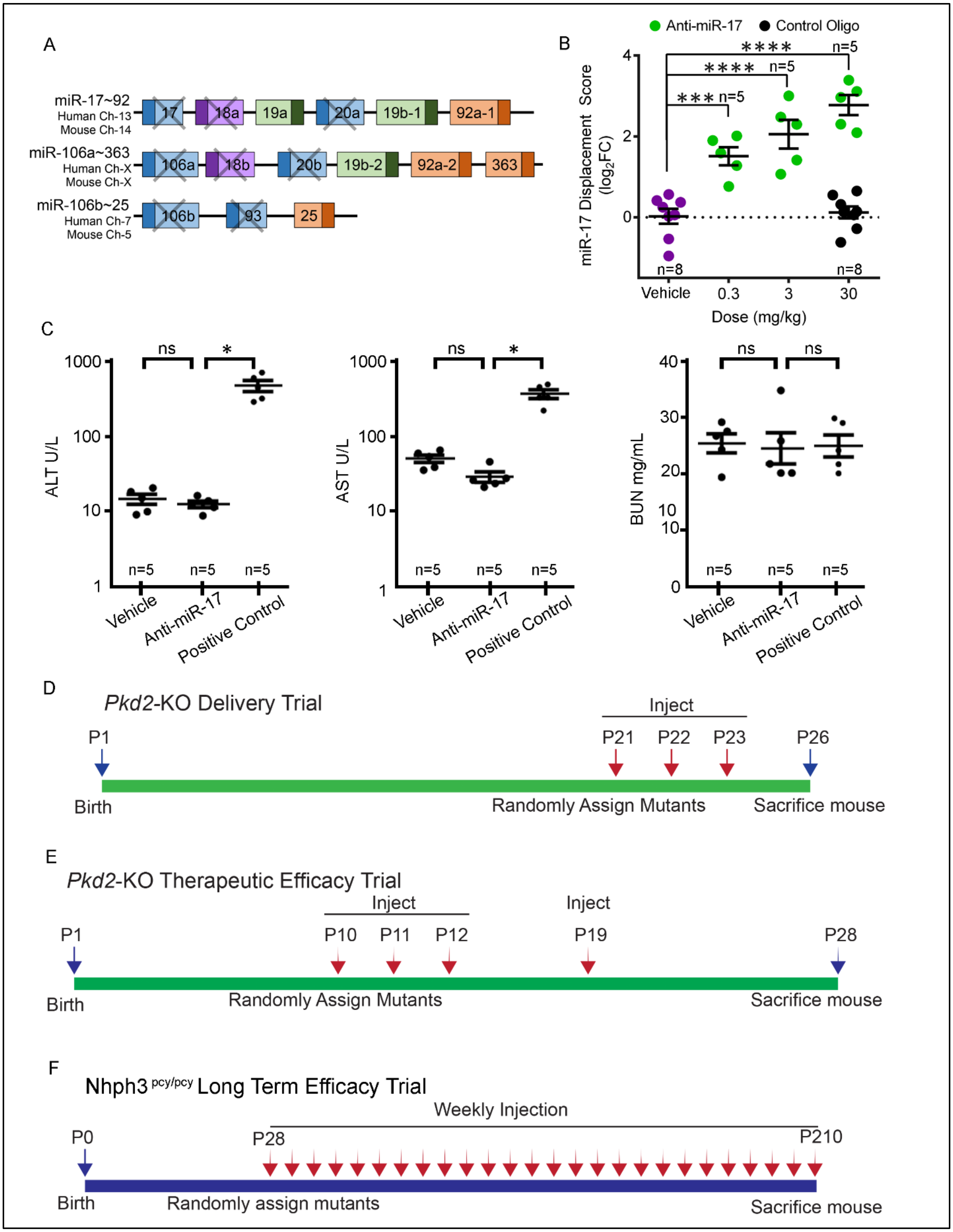
A
miR-17~92
Human Ch-13
Mouse Ch-14
17
18a
19a
20a
19b-1
92a-1
miR-106a~363
Human Ch-X
Mouse Ch-X
106a
18b
20b
19b-2
92a-2
363
miR-106b~25
Human Ch-7
Mouse Ch-5
106b
93
25
B
Anti-miR-17
Control Oligo
miR-17 Displacement Score (log2FC)
Dose (mg/kg)
Vehicle
0.3
3
30
n=8
n=5
C
ALT U/L
AST U/L
BUN mg/mL
ns
*
Vehicle
Anti-miR-17
Positive Control
D
Pkd2-KO Delivery Trial
Inject
P1
P21
P22
P23
P26
Birth
Randomly Assign Mutants
Sacrifice mouse
E
Pkd2-KO Therapeutic Efficacy Trial
Inject
P1
P10
P11
P12
P19
P28
Birth
Randomly Assign Mutants
Sacrifice mouse
F
Nhph3 pcy/pcy Long Term Efficacy Trial
Weekly Injection
P0
P28
P210
Birth
Randomly assign mutants
Sacrifice mouse

Supplementary Figure 5: (A) Genomic organization of the miR-17~92 and its paralogous clusters miR-106a~363 and miR-106b~25 is shown. Collectively, these three miRNA clusters encode miRNAs that belong to either the miR-17 (blue), miR-19 (green), miR-18 (purple) or miR-25 (orange) miRNA families. We have previously shown that our anti-miR-17 compound inhibits miRNAs that are marked with an 'X' in cultured cells. (B) The ability of this compound to inhibit miR-17 *in vivo* was measured using the miRNA polysome shift assay (miPSA)[1]. Normal mice were treated with a single subcutaneous dose of vehicle (PBS), anti-miR-17 (0.3, 3 or 30 mg per kg dose) or control oligonucleotide (30 mg per kg dose), and kidneys were harvested 7-days later (n=5-7 each group and dose). miR-17 fraction (normalized to let-7d) in high molecular weight (HMW) was measured by Q-PCR. miR-17 was displaced from HMW polysomes in kidneys of mice injected with anti-miR-17 in a dose-dependent manner. Similar miR-17 displacement was not observed in mice treated with vehicle or a control oligonucleotide. (C) Normal adult mice were injected with 300 mg per kg dose of anti-miR-17, vehicle or a control oligonucleotide known to produce liver toxicity (positive control). Measurement of the liver (ALT and AST) and kidney function (BUN) showed that anti-miR-17 does not produce acute liver or kidney toxicity. (D-F) The design of the delivery and therapeutic efficacy trials are shown. Error bars indicate SEM. * indicates $P<0.05$, ns indicates $P>0.05$. One-way ANOVA, Tukey's multiple comparisons test (B) Student's unpaired *t*-test (C)

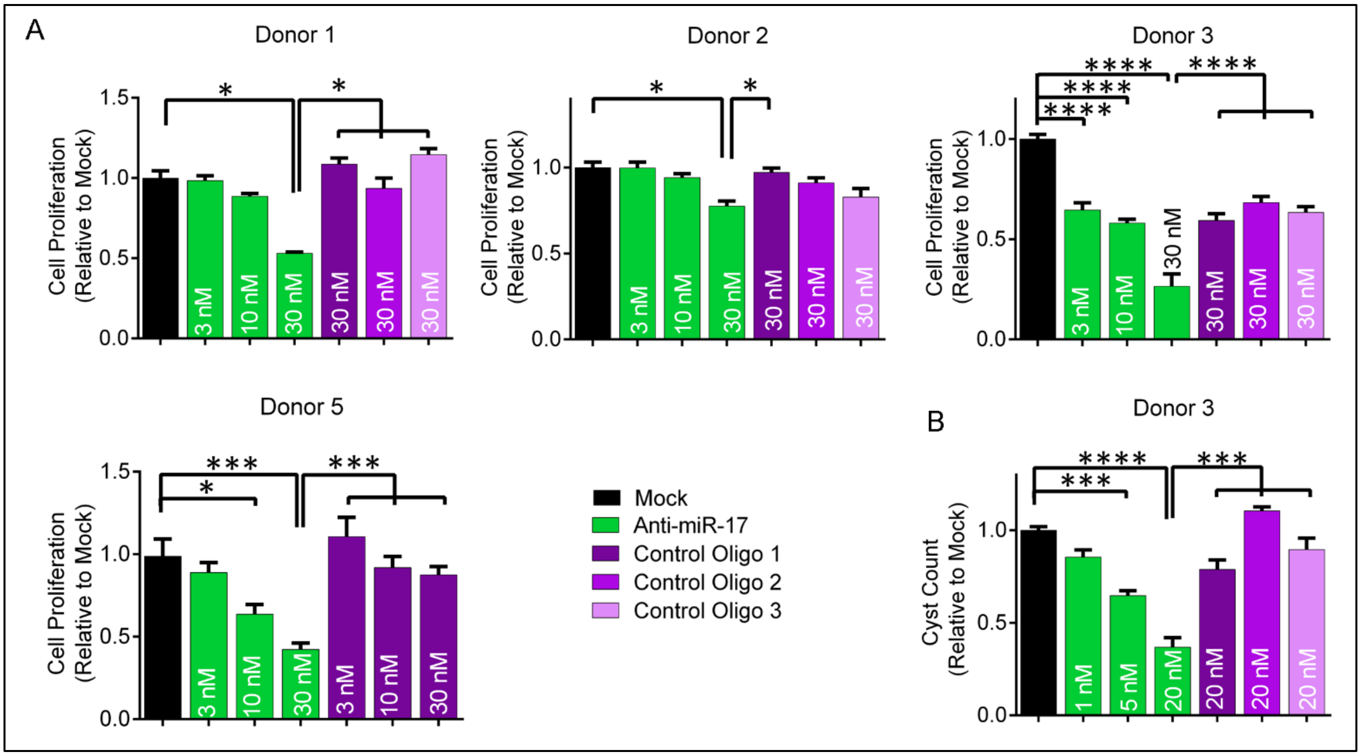

Supplementary Figure 6: Primary cyst epithelial cultures derived from kidneys of ADPKD patients were transfected with anti-miR-17 (dose: 3 nM, 10 nM or 30 nM) or three different control oligonucleotides (dose: 30 nM, control oligo1, 2 and 3) or mock-transfected. These cells were then cultured to measure proliferation and *in-vitro* cyst formation. (A) Data for proliferation (n=5 for each treatment and dose) using primary cultures derived from ADPKD Donor1, 2, 3 and 5. (B) *In-vitro* cyst formation (n=3 for each treatment and dose) using primary cultures derived from ADPKD Donor 3. Anti-miR-17 reduced cell viability and cyst count in a dose-dependent manner. Error bars indicate SEM. *indicates P<0.05, **indicates ns indicates P<0.01, ***indicates ns indicates P<0.005, and ****indicates P<0.001. One-way ANOVA, Tukey's multiple comparisons test

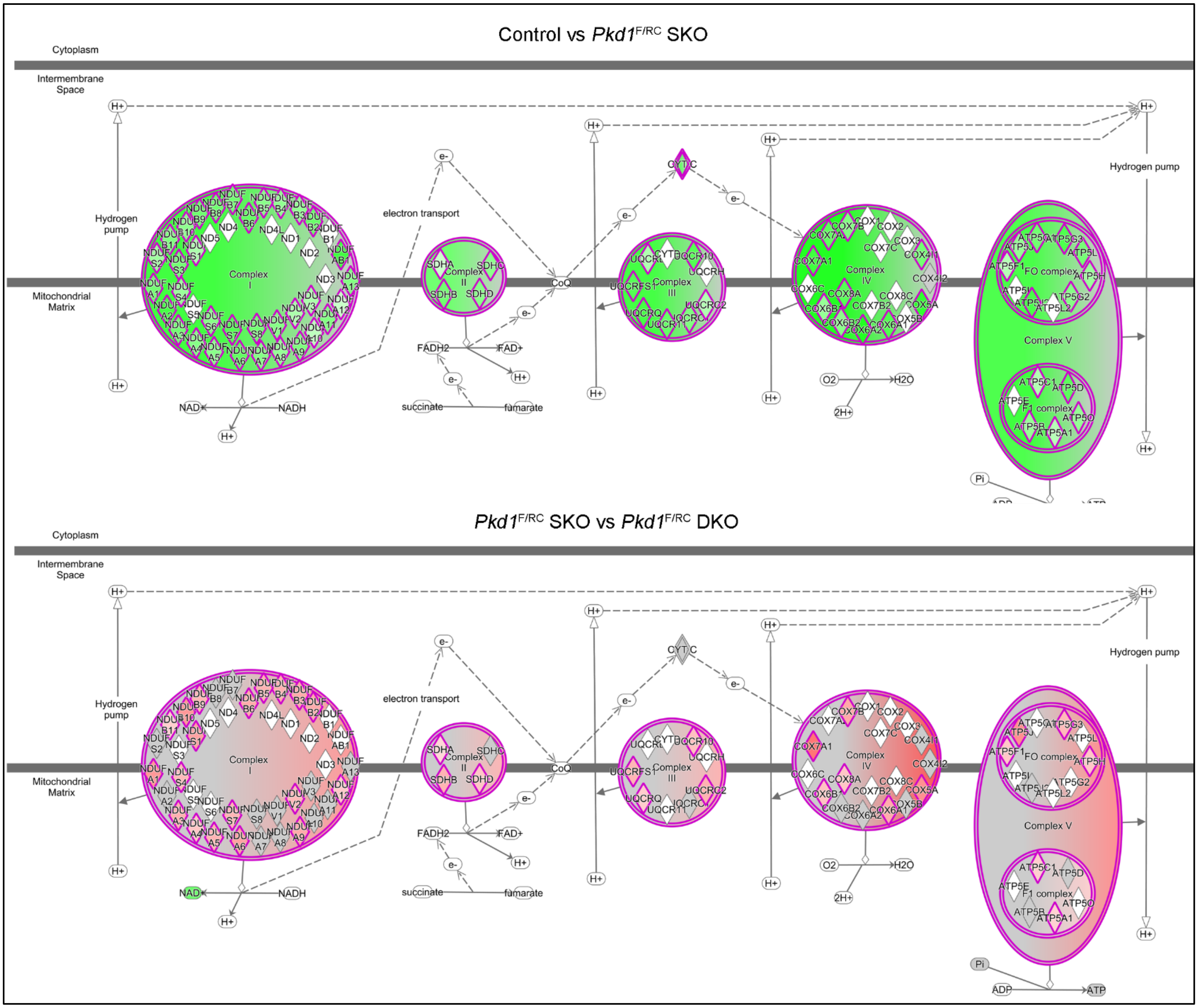

Supplementary Figure 7: RNA-Seq analysis was performed to compare mRNA expression profiles between kidneys of 21-day-old control, *Pkd1*$^{F/RC}$SKO and *Pkd1*$^{F/RC}$DKO mice (n=3). The differentially expressed genes (P<0.05, FDR<0.1) were further analyzed using the Ingenuity Pathway Analysis (IPA) software. Several mitochondrial pathways were differentially expressed. In particular, the oxidative phosphorylation pathway (shown above) was downregulated in *Pkd1*$^{F/RC}$SKO compared to control kidneys. In contrast, this pathway was upregulated in *Pkd1*$^{F/RC}$DKO compared to *Pkd1*$^{F/RC}$SKO kidneys. Green indicates downregulated genes, whereas red indicates upregulated genes.

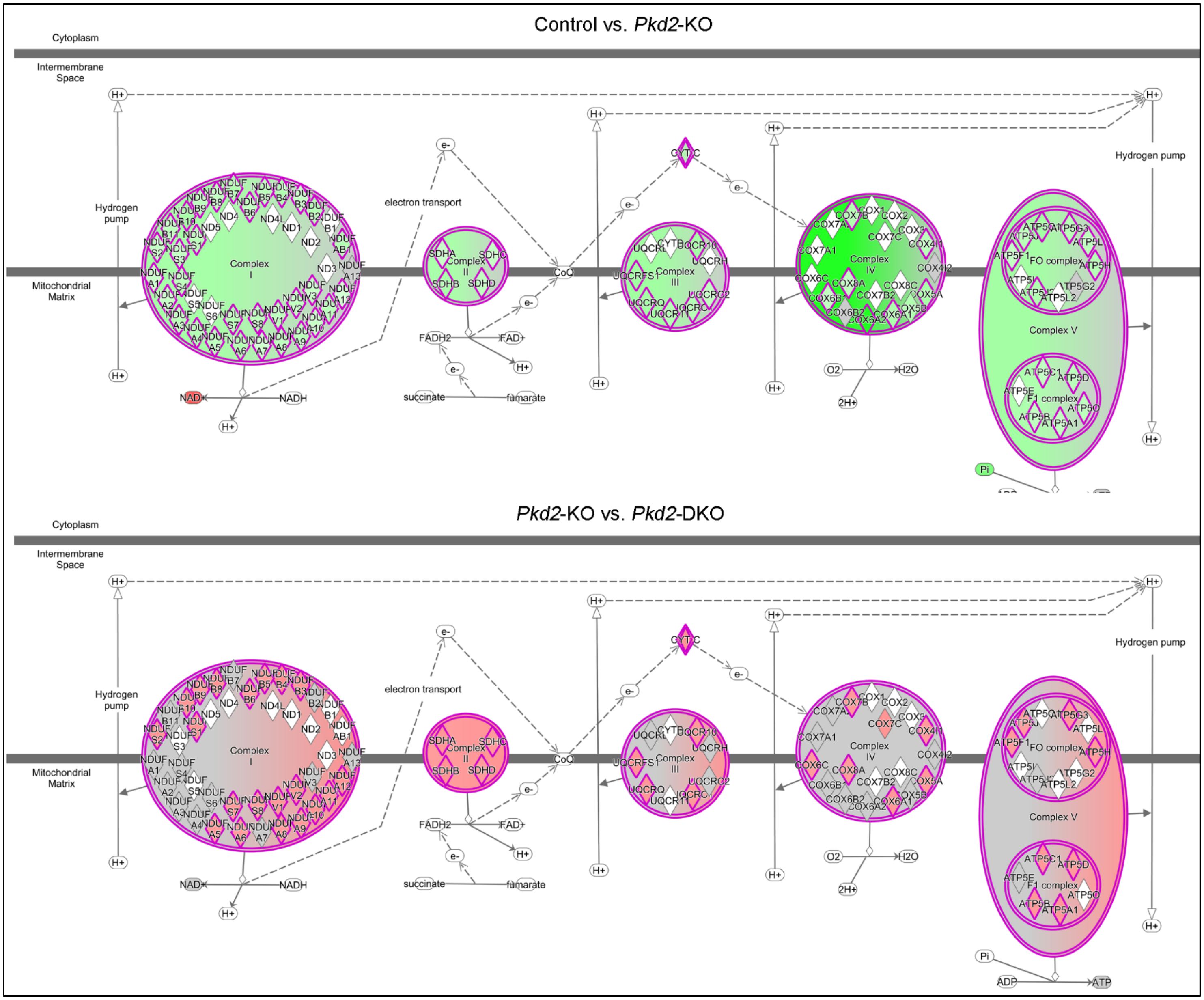

Supplementary Figure 8: RNA-Seq analysis was performed to compare mRNA expression profiles between kidneys of 21-day-old control, *Pkd2*-KO and *Pkd2*-miR-17~92KO mice (n=5). The differentially expressed genes (P<0.05, FDR<0.1) were further analyzed using the Ingenuity Pathway Analysis (IPA) software. Similar to the *Pkd1*-mutant mice, several mitochondrial pathways were differentially expressed. The oxidative phosphorylation pathway (shown above) was downregulated in *Pkd2*-KO compared to control kidneys. In contrast, this pathway was upregulated in *Pkd2*-miR-17~92KO compared to *Pkd2*-KO kidneys. Green indicates downregulated genes, whereas red indicates upregulated genes.

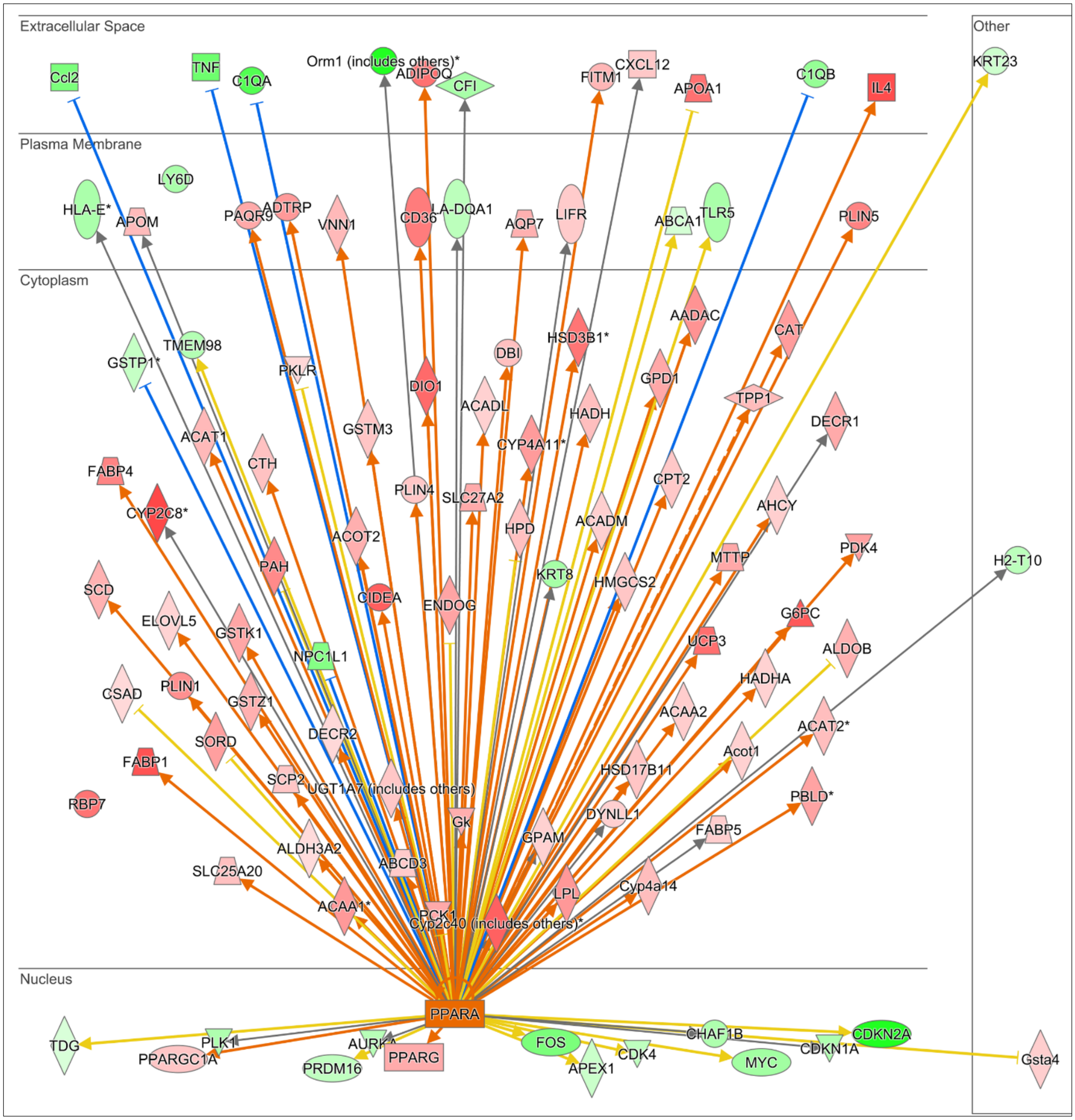

Supplementary Figure 9: RNA-Seq analysis was performed to compare mRNA expression profiles between kidneys of 21-day-old control, *Pkd1*$^{F/RC}$SKO and *Pkd1*$^{F/RC}$DKO mice (n=3). The differentially expressed genes (P<0.05, FDR<0.1) were further analyzed using the Ingenuity Pathway Analysis (IPA) software to identify the upstream regulators that may be responsible for the gene expression changes observed after miR-17~92 deletion. PPARA-regulated gene network was predicted to be robustly activated after miR-17~92 deletion. Accordingly, a large number of direct PPARA targets (shown above) were upregulated in *Pkd1*$^{F/RC}$DKO compared to *Pkd1*$^{F/RC}$SKO kidneys. Green indicates downregulated genes, whereas red indicates upregulated genes.

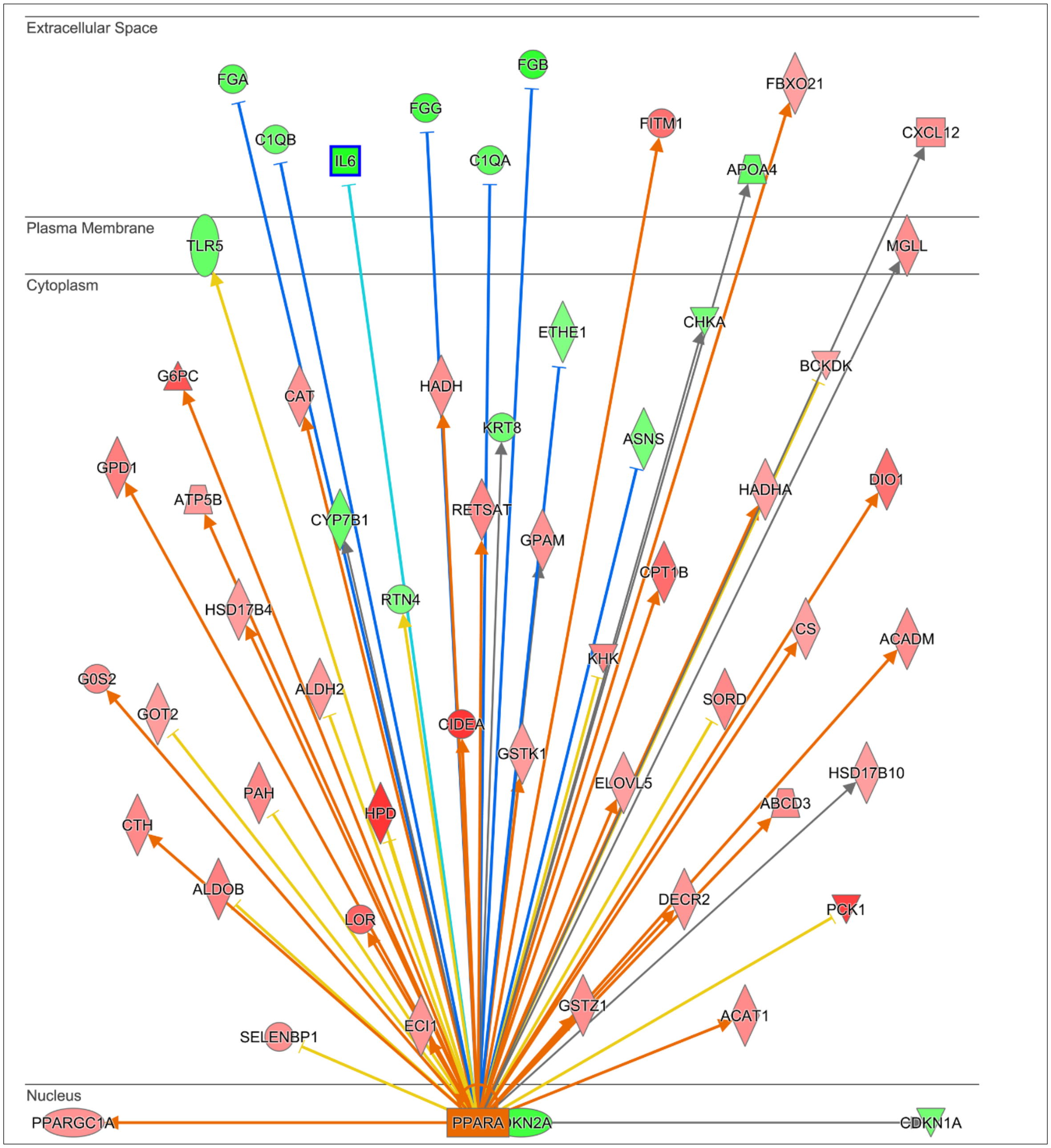

Supplementary Figure 10: RNA-Seq analysis was performed to compare mRNA expression profiles between kidneys of 21-day-old control, *Pkd2*-KO and *Pkd2*-miR-17~92KO mice (n=5). The differentially expressed genes (P<0.05, FDR<0.1) were further analyzed using the Ingenuity Pathway Analysis (IPA) software to identify the upstream regulators that may be responsible for the gene expression changes observed after miR-17~92 deletion in *Pkd2*-KO mice. Similar to *Pkd1*-mutant kidneys, PPARA-regulated gene network was predicted to be robustly activated after miR-17~92 deletion in *Pkd2*-KO kidneys. A number of direct PPARA targets (shown above) were upregulated in *Pkd2*-miR-17~92KO compared to *Pkd2*-KO kidneys. Green indicates downregulated genes, whereas red indicates upregulated genes.

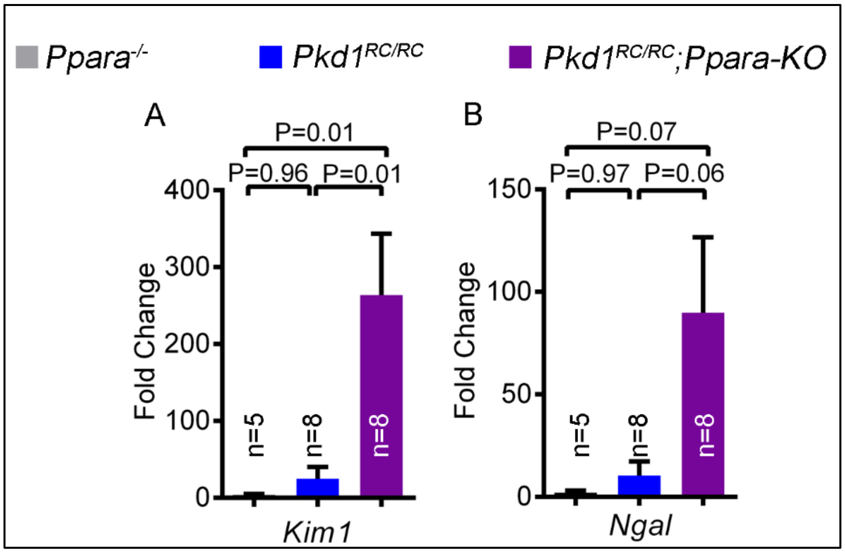

Supplementary Figure 11: To test whether reduced *Ppara* gene dosage is sufficient to enhance proliferation and promote cyst formation, 6-week old *Ppara*$^{-/-}$, *Pkd1*$^{RC/RC}$, and *Pkd1*$^{RC/RC}$; *Ppara*-KO mice were characterized. Q-PCR analysis (A) *Kim1* and (B) *Ngal* in the indicated mouse models is shown. Error bars indicate SEM. Multiplicity adjusted P values using Tukey's multiple comparison test are shown.

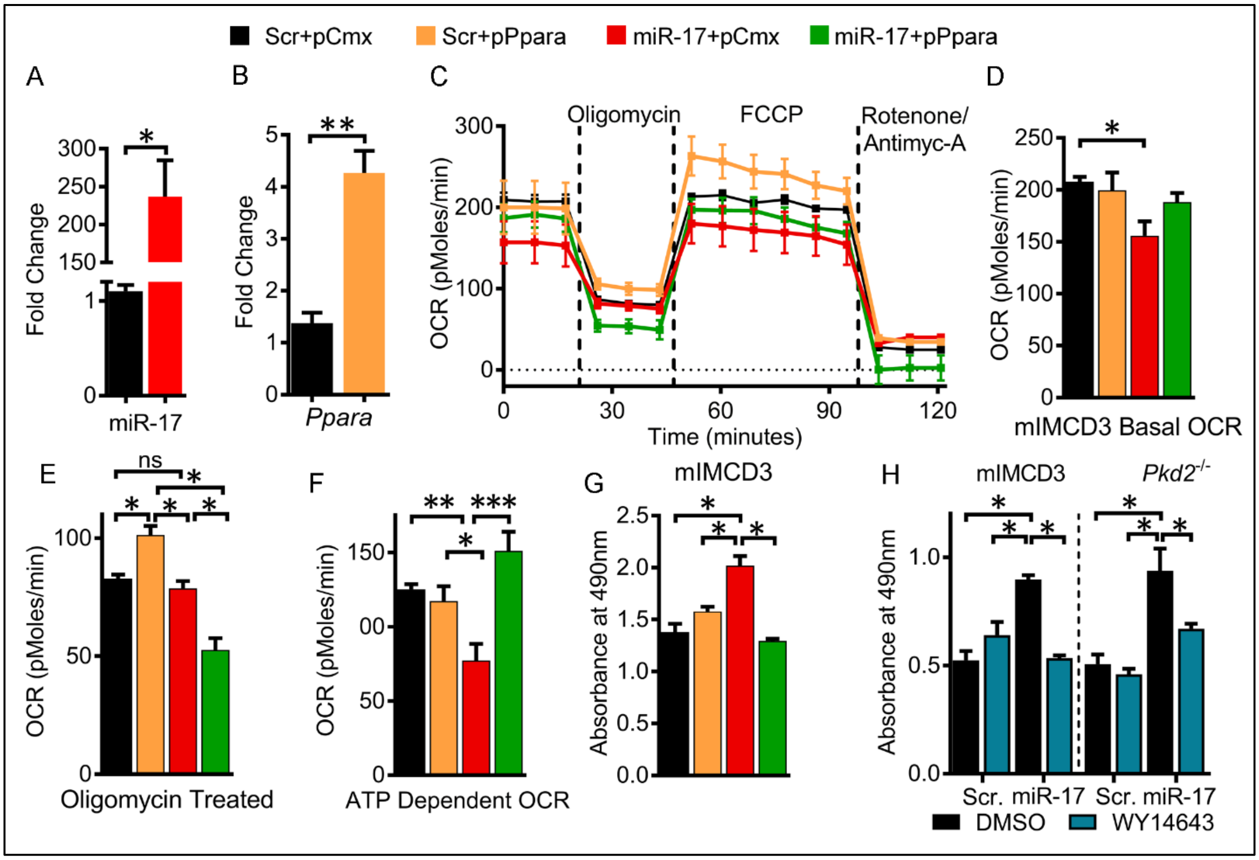

Supplementary Figure 12: Characterization of cultured cells transfected with (A) miR-17 mimics and (B) PPARA-expressing plasmid (pPpara) is shown. To determine whether miR-17 affects OXPHOS, *Seahorse* XF 24 Analyzer was used to measure real-time mitochondrial oxygen consumption rate (OCR) of renal epithelial cells. (C) Real-time OCR tracings, (D) Basal OCR, (E) Oligomycin-treated OCR, and (F) ATP-dependent OCR of indicated cell lines is shown. ATP-dependent OCR was reduced in mIMCD3 cells treated with miR-17 mimics compared to scramble mimics. Reexpression of *Ppara* prevented miR-17-induced reductions in ATP-dependent OCR. (G&H) MTT assay was used to determine whether increased *Ppara* gene dosage inhibits miR-17-stimulated proliferation of renal epithelia. Treatment with miR-17 mimic increased proliferation of mIMCD3 cells. (G) Transfection with a *Ppara*-expressing plasmid (pPpara) reduced miR-17-stimulated proliferation whereas transfection with a control plasmid (pCMX) had no effect (n=3 biological replicates with three technical replicates). (H) Treatment with WY14643, a PPARA agonist, also reduced miR-17-stimulated proliferation whereas treatment with DMSO had no effect (n=3 biological replicates with three technical replicates). miR-17 mimic also increased proliferation of *Pkd2*$^{-/-}$ kidney epithelia. Similar to mIMCD3 cells, WY14643 reduced miR-17-stimulated proliferation of *Pkd2* $^{-/-}$ cells whereas treatment with DMSO had no effect (n=3 biological replicates with three technical replicates). One-way ANOVA, Tukey's multiple comparisons test (D-H) Student's unpaired *t*-test (A, B)

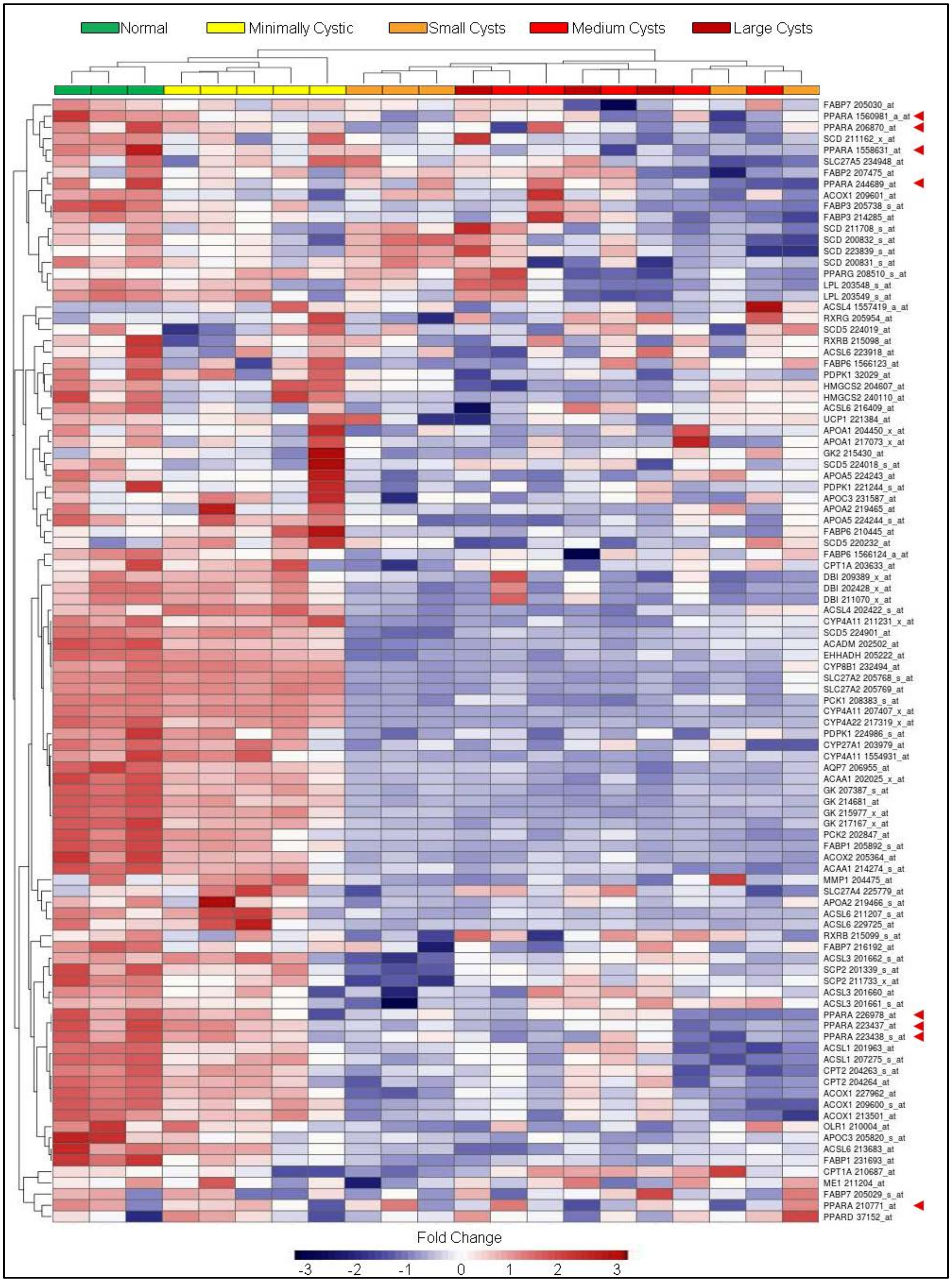

Supplementary Figure 13: Song *et al*[2], have previously performed cDNA microarrays (Human Genome U133 Plus 2.0 Array, Affymetrix) using RNA from three normal renal cortical samples, five minimally cystic ADPKD tissue samples and 13 ADPKD cysts of varying sizes (small, medium, and large). Analysis of this publically-available data (http://rged.wall-eva.net/) revealed that *PPARA* (red arrow heads) and its targets are downregulated in human ADPKD cysts.

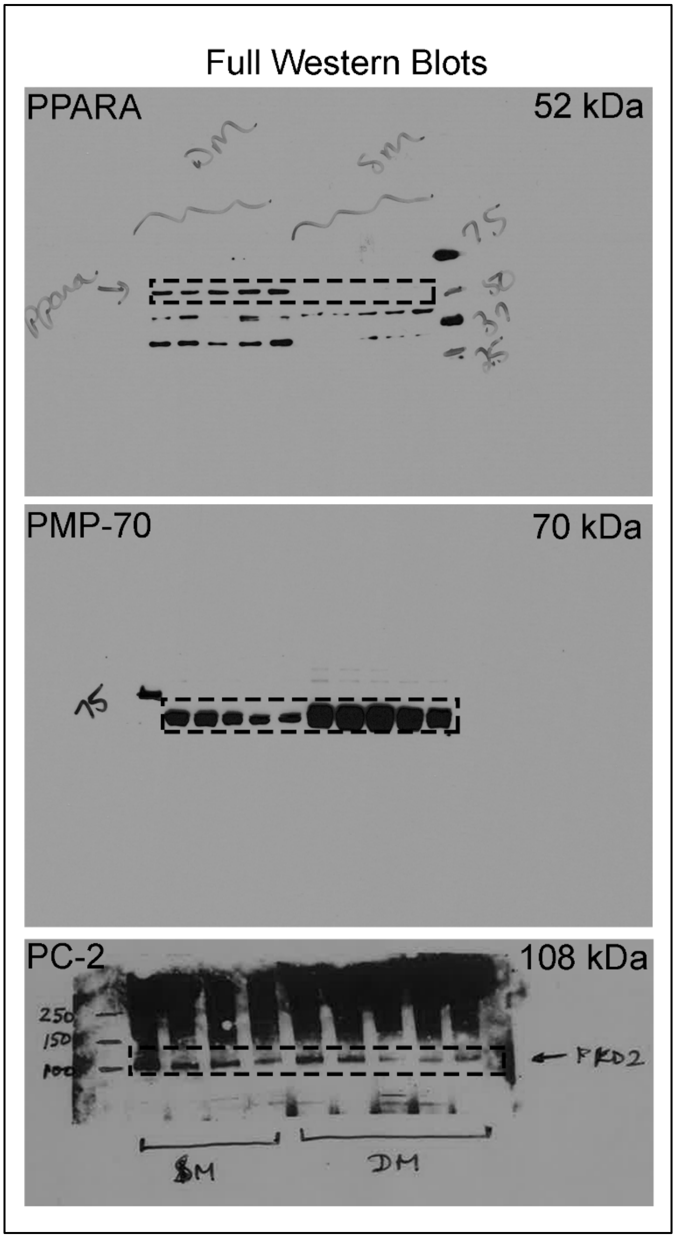

Supplementary Figure 14: The full, original Western blots are shown accompanied with indicated protein name (top left) and molecular weight (top right).

Supplementary Table 1:Differential expression of miRNAs in ADPKD models

miRNA	Pkd1-KO vs. Control				Pkd2-KO vs. Control			
	P10 AvgExp	Fold-change	P-value	FDR	P21 AvgExp	Fold-change	P-value	FDR
mmu-miR-29a	9.57	2.73	1.5E-05	0.00403	11.66	1.20	8.4E-02	0.37106
mmu-miR-126-3p	14.57	0.64	2.1E-05	0.00403	14.54	0.75	2.6E-04	0.00742
mmu-miR-200c	10.35	1.77	2.8E-05	0.00403	10.35	1.65	1.9E-03	0.02635
mmu-miR-96	9.83	3.91	3.0E-05	0.00403	9.44	5.54	2.4E-05	0.00182
mmu-miR-182	3.29	59.71	4.4E-05	0.00469	3.03	66.41	3.7E-06	0.00050
mmu-let-7g	13.35	0.81	6.0E-05	0.00524	13.46	0.82	5.5E-03	0.05630
mmu-miR-20a/b	12.20	1.23	6.8E-05	0.00524	11.43	1.51	8.2E-04	0.01637
mmu-miR-218	9.98	1.59	8.2E-05	0.00553	9.33	1.01	9.1E-01	1.00000
mmu-miR-183	8.28	4.80	9.7E-05	0.00578	7.81	3.88	1.4E-04	0.00454
mmu-miR-192	9.40	0.38	1.3E-04	0.00676	10.57	0.56	1.3E-03	0.01998
mmu-miR-204	9.96	0.44	1.8E-04	0.00856	10.24	0.66	2.0E-04	0.00608
mmu-miR-21	12.59	2.85	2.0E-04	0.00856	12.90	4.67	3.7E-06	0.00050
mmu-miR-196b	11.35	0.81	2.1E-04	0.00856	10.60	0.86	5.0E-02	0.26058
mmu-miR-194	9.86	0.40	2.7E-04	0.01041	10.74	0.64	2.2E-02	0.14702
mmu-let-7i	13.11	1.56	3.4E-04	0.01232	12.68	1.76	5.0E-05	0.00268
mmu-miR-199a-5p	12.49	1.35	3.7E-04	0.01257	10.87	2.54	9.1E-08	0.00005
mmu-miR-29b	7.27	2.83	4.1E-04	0.01289	9.66	1.37	2.0E-01	0.61661
mmu-miR-30e	10.21	0.64	4.6E-04	0.01375	10.68	0.61	2.7E-05	0.00182
mmu-miR-221	8.43	1.47	6.9E-04	0.01939	8.51	1.11	1.9E-01	0.59419
mmu-miR-100	9.53	1.35	7.6E-04	0.01943	8.89	1.43	2.3E-03	0.02940
mmu-miR-378	9.58	0.73	7.6E-04	0.01943	10.42	0.67	3.5E-03	0.03950
mmu-miR-223	7.48	2.54	8.0E-04	0.01964	7.99	4.16	7.1E-06	0.00076
mmu-miR-126-5p	10.56	0.70	9.0E-04	0.02103	10.59	0.78	1.2E-03	0.01951
mmu-miR-26b	10.10	0.71	1.4E-03	0.03028	10.35	0.76	8.1E-05	0.00335
mmu-miR-143	11.94	1.33	1.5E-03	0.03211	11.62	1.32	5.7E-02	0.28601
mmu-miR-199a-3p	12.68	1.28	1.6E-03	0.03384	11.23	2.84	1.4E-06	0.00038
mmu-miR-30c	12.78	0.67	1.7E-03	0.03425	13.00	0.64	2.6E-05	0.00182
mmu-miR-106a+mmu-miR-17	11.67	1.21	1.9E-03	0.03616	10.89	1.45	1.4E-04	0.00454
mmu-miR-351	6.50	2.22	2.5E-03	0.04499	3.60	0.43	1.9E-01	0.61071
mmu-miR-362-5p	4.37	2.11	2.5E-03	0.04499	3.92	1.83	2.3E-01	0.70273
mmu-miR-28	9.54	1.27	2.9E-03	0.04945	9.03	1.48	1.9E-03	0.02635
mmu-miR-340-5p	10.06	0.86	2.9E-03	0.04945	9.53	0.98	7.5E-01	1.00000
mmu-miR-151-5p	9.86	0.71	3.1E-03	0.04977	9.89	0.78	2.5E-02	0.15763
mmu-miR-375	1.50	7.96	3.4E-03	0.05350	5.26	2.50	4.7E-03	0.04931
mmu-miR-714	6.23	1.87	3.7E-03	0.05626	5.24	13.49	8.0E-02	0.36446
mmu-miR-200a	11.43	0.76	4.0E-03	0.05921	11.90	0.81	1.4E-02	0.10825
mmu-miR-148b	8.77	0.75	4.1E-03	0.05921	8.61	0.95	5.5E-01	1.00000
mmu-miR-185	6.02	0.67	4.4E-03	0.06098	6.51	0.70	9.5E-02	0.38502
mmu-miR-431	2.84	12.32	4.5E-03	0.06098	0.45	1.85	3.6E-01	0.73909
mmu-miR-345-5p	6.39	0.73	4.5E-03	0.06098	6.41	0.72	2.9E-02	0.17547
mmu-miR-1839-5p	7.39	0.72	5.6E-03	0.07323	7.17	0.63	1.1E-04	0.00427
mmu-miR-196a	10.09	0.82	5.8E-03	0.07365	9.66	0.79	2.5E-02	0.15930
mmu-miR-99a	10.82	0.63	5.9E-03	0.07365	10.86	0.74	2.9E-03	0.03403
mmu-miR-350	8.20	0.75	6.7E-03	0.08141	7.76	1.72	8.7E-04	0.01670
mmu-miR-532-5p	7.61	1.28	7.4E-03	0.08879	7.54	1.17	2.2E-02	0.14544
mmu-miR-132	7.51	1.69	8.6E-03	0.10007	7.70	2.24	3.9E-04	0.00990
mmu-miR-410	5.79	3.25	9.5E-03	0.10596	3.35	2.21	1.7E-01	0.55676
mmu-miR-200b	11.94	0.85	9.7E-03	0.10596	12.08	0.83	5.6E-03	0.05688

mmu-miR-1951	6.91	1.21	9.7E-03	0.10596	7.18	1.15	7.8E-02	0.35893
mmu-miR-188-5p	9.06	0.86	9.9E-03	0.10596	9.13	0.99	8.1E-01	1.00000
mmu-miR-135a	6.68	0.48	1.0E-02	0.10931	5.12	1.07	3.1E-01	0.73909
mmu-miR-150	8.04	0.53	1.1E-02	0.11236	8.93	1.04	8.6E-01	1.00000
mmu-miR-195	5.77	0.67	1.1E-02	0.11244	6.48	1.04	7.6E-01	1.00000
mmu-miR-301a	10.13	1.16	1.2E-02	0.11244	9.47	0.96	4.9E-02	0.25880
mmu-miR-10b	11.76	1.15	1.2E-02	0.11244	11.12	1.15	9.3E-02	0.38502
mmu-miR-434-3p	6.56	2.01	1.2E-02	0.11244	3.90	4.71	1.7E-01	0.55037
mmu-miR-222	6.24	1.39	1.2E-02	0.11554	6.70	1.07	6.7E-01	1.00000
mmu-miR-376b	5.61	2.31	1.2E-02	0.11554	3.07	4.45	1.5E-01	0.51487
mmu-miR-329	5.68	2.53	1.3E-02	0.11864	2.54	10.06	1.5E-02	0.11251
mmu-miR-140	8.88	0.76	1.4E-02	0.12447	9.11	0.84	2.1E-02	0.13969
mmu-miR-9	9.13	1.42	1.4E-02	0.12544	9.09	2.32	1.5E-03	0.02339
mmu-miR-26a	9.80	0.75	1.4E-02	0.12544	9.65	0.97	6.3E-01	1.00000
mmu-miR-411	5.00	2.91	1.6E-02	0.13319	2.01	4.69	8.8E-02	0.38299
mmu-miR-376c	6.02	3.10	1.6E-02	0.13446	4.01	2.19	9.2E-02	0.38502
mmu-miR-23a	11.15	1.20	1.8E-02	0.14539	11.41	1.04	3.2E-01	0.73909
mmu-miR-214	7.67	1.48	1.9E-02	0.15137	5.88	3.56	3.2E-03	0.03702
mmu-miR-335-5p	9.16	0.66	1.9E-02	0.15461	7.22	0.64	8.5E-03	0.07274
mmu-miR-181c	7.75	0.68	2.0E-02	0.15461	7.53	0.82	1.8E-02	0.12535
mmu-miR-379	1.53	8.32	2.1E-02	0.16152	0.00	1.00	1.0E+00	1.00000
mmu-miR-23b	11.21	0.81	2.1E-02	0.16152	11.55	0.78	6.8E-04	0.01468
mmu-let-7d	13.71	0.96	2.1E-02	0.16152	13.47	0.91	3.8E-02	0.21030
mmu-miR-127	6.63	2.69	2.3E-02	0.16809	4.74	2.60	8.2E-03	0.07178
mmu-miR-101a	8.31	0.73	2.4E-02	0.17543	8.72	0.74	1.9E-03	0.02635
mmu-miR-93	9.44	1.36	2.7E-02	0.19324	8.75	1.47	1.0E-03	0.01731
mmu-let-7a	14.18	0.88	2.8E-02	0.20385	14.09	0.88	1.8E-02	0.12535
mmu-miR-615-3p	4.55	0.46	3.1E-02	0.21785	3.42	1.27	5.6E-01	1.00000
mmu-miR-708	5.29	0.57	3.2E-02	0.22232	2.45	0.35	1.5E-01	0.50663
mmu-miR-1198	5.50	0.66	3.4E-02	0.23055	5.43	0.79	1.2E-01	0.44042
mmu-miR-690	2.07	0.22	3.4E-02	0.23055	2.12	1.03	9.7E-01	1.00000
mmu-miR-142-3p	8.87	1.68	3.6E-02	0.23959	9.67	4.69	6.0E-04	0.01412
mmu-miR-125a-3p	7.67	1.57	3.8E-02	0.24546	6.92	2.29	6.7E-02	0.32547
mmu-miR-148a	10.75	0.74	3.8E-02	0.24546	10.23	0.92	5.4E-01	1.00000
mmu-miR-551b	3.89	9.76	3.8E-02	0.24546	2.78	5.84	2.3E-02	0.14906
mmu-miR-425	7.03	0.79	3.8E-02	0.24546	7.34	0.82	3.1E-02	0.18013
mmu-miR-24	10.27	1.15	4.3E-02	0.26755	9.98	1.40	1.6E-03	0.02373
mmu-miR-15a	12.04	1.07	4.4E-02	0.26755	11.97	1.34	5.9E-03	0.05808
mmu-miR-187	5.12	0.36	4.4E-02	0.26755	5.50	0.39	3.1E-02	0.18125
mmu-miR-130b	8.53	0.75	4.4E-02	0.26755	6.81	1.07	6.8E-01	1.00000
mmu-miR-210	6.72	1.43	4.7E-02	0.28473	6.63	1.42	7.1E-02	0.33367
mmu-miR-328	6.62	0.69	4.8E-02	0.28473	6.94	0.62	2.8E-03	0.03399
mmu-miR-191	9.25	0.85	4.8E-02	0.28473	9.32	1.04	6.4E-01	1.00000
mmu-miR-338-3p	6.55	0.50	5.1E-02	0.29489	6.56	0.60	2.6E-02	0.15930
mmu-miR-141	9.06	1.60	5.1E-02	0.29714	9.49	1.42	1.1E-03	0.01810
mmu-let-7f	13.09	0.93	5.2E-02	0.29964	13.11	0.82	7.9E-03	0.07042
mmu-miR-335-3p	6.50	0.79	5.3E-02	0.30090	3.62	3.36	1.1E-01	0.40841
mmu-miR-22	12.63	1.14	5.4E-02	0.30457	13.41	0.99	7.1E-01	1.00000
mmu-miR-382	5.53	2.58	6.4E-02	0.35675	2.57	6.95	8.0E-02	0.36446
mmu-miR-139-5p	5.66	0.37	6.5E-02	0.35845	6.04	0.84	1.3E-01	0.46334
mmu-miR-324-5p	8.01	0.84	6.7E-02	0.36220	7.09	1.22	3.1E-02	0.18125
mmu-miR-484	8.32	0.91	6.8E-02	0.36220	7.94	1.31	2.7E-02	0.16268
mmu-miR-450a-5p	10.36	0.83	6.9E-02	0.36220	9.23	0.70	2.2E-03	0.02910
mmu-miR-331-3p	7.03	1.21	6.9E-02	0.36220	6.16	1.22	2.5E-01	0.73027
mmu-miR-202-5p	4.57	1.49	7.1E-02	0.36935	4.49	0.86	6.5E-01	1.00000

mmu-miR-30b	12.29	0.84	7.2E-02	0.36935	12.72	1.05	5.2E-01	1.00000
mmu-miR-433	4.09	7.96	7.3E-02	0.37111	0.98	2.66	2.1E-01	0.64896
mmu-miR-434-5p	4.81	1.87	7.8E-02	0.39354	2.03	16.56	3.1E-05	0.00182
mmu-miR-872	8.35	0.84	7.8E-02	0.39354	7.71	1.10	1.1E-01	0.40841
mmu-miR-762	7.59	2.02	8.0E-02	0.39851	7.45	5.34	2.4E-02	0.15570
mmu-miR-16	13.38	1.09	8.1E-02	0.40015	13.38	1.24	2.3E-03	0.02940
mmu-miR-495	5.45	1.77	8.8E-02	0.43133	3.84	2.65	1.1E-01	0.41370
mmu-miR-134	2.61	4.56	9.1E-02	0.44113	0.00	1.00	1.0E+00	1.00000
mmu-miR-107	9.30	0.44	9.2E-02	0.44183	9.33	0.95	6.4E-01	1.00000
mmu-miR-802	4.38	0.19	9.6E-02	0.44663	5.89	0.42	1.1E-02	0.08482
mmu-miR-377	7.35	1.46	9.7E-02	0.44663	6.42	1.37	5.3E-02	0.27256
mmu-miR-1941-3p	2.81	0.24	1.0E-01	0.44663	1.28	0.76	7.8E-01	1.00000
mmu-miR-717	2.01	4.21	1.0E-01	0.44663	2.12	0.46	2.8E-01	0.73909
mmu-miR-770-3p	1.20	0.19	1.0E-01	0.44663	0.00	1.00	1.0E+00	1.00000
mmu-miR-873	1.18	5.11	1.0E-01	0.44663	0.00	1.00	1.0E+00	1.00000
mmu-miR-1187	0.75	0.35	1.0E-01	0.44663	0.45	1.86	3.6E-01	0.73909
mmu-miR-322	11.00	1.23	1.0E-01	0.44663	9.70	1.03	7.9E-01	1.00000
mmu-miR-759	1.02	4.08	1.0E-01	0.44663	0.30	1.51	3.6E-01	0.73909
mmu-miR-669e	0.88	3.39	1.0E-01	0.44663	0.11	0.86	3.6E-01	0.73909
mmu-miR-18a	0.92	3.60	1.0E-01	0.44663	0.00	1.00	1.0E+00	1.00000
mmu-miR-493	1.00	3.98	1.1E-01	0.48339	0.00	1.00	1.0E+00	1.00000
mmu-miR-290-5p	0.95	3.71	1.2E-01	0.51831	0.00	1.00	1.0E+00	1.00000
mmu-miR-144	10.02	0.54	1.2E-01	0.52051	10.73	2.17	1.6E-01	0.53314
mmu-miR-412	0.21	1.34	1.2E-01	0.52051	0.00	1.00	1.0E+00	1.00000
mmu-let-7b	13.43	1.13	1.2E-01	0.52051	13.76	1.02	4.9E-01	0.99495
mmu-miR-30a	11.96	0.83	1.3E-01	0.52992	12.20	0.79	1.1E-02	0.08482
mmu-miR-146a	8.91	0.89	1.3E-01	0.54734	9.12	1.82	4.0E-03	0.04488
mmu-miR-676	6.21	1.27	1.5E-01	0.60942	5.81	1.02	9.3E-01	1.00000
mmu-miR-145	13.50	1.09	1.5E-01	0.61019	13.40	0.87	4.6E-02	0.24881
mmu-miR-138	6.36	0.92	1.6E-01	0.64734	5.71	1.25	1.0E-02	0.08172
mmu-miR-451	10.67	0.54	1.6E-01	0.64734	11.89	2.02	1.9E-01	0.59419
mmu-miR-3471	4.62	2.41	1.6E-01	0.64734	2.28	1.37	7.4E-01	1.00000
mmu-miR-31	5.79	1.60	1.6E-01	0.64932	5.70	3.16	3.1E-04	0.00832
mmu-miR-743a	0.96	0.39	1.8E-01	0.68475	0.00	1.00	1.0E+00	1.00000
mmu-miR-190b	7.82	1.09	1.8E-01	0.68475	8.02	1.21	4.5E-03	0.04893
mmu-miR-342-3p	8.64	0.91	1.8E-01	0.68475	8.33	1.06	5.3E-01	1.00000
mmu-miR-34a	6.57	1.36	1.8E-01	0.68475	6.82	1.25	7.0E-02	0.33367
mmu-miR-466c-5p	1.50	0.24	1.9E-01	0.72193	0.00	1.00	1.0E+00	1.00000
mmu-miR-574-3p	7.23	1.24	1.9E-01	0.72769	6.92	1.60	1.8E-02	0.12535
mmu-miR-409-5p	0.63	2.41	2.1E-01	0.77229	0.00	1.00	1.0E+00	1.00000
mmu-miR-695	1.14	2.76	2.1E-01	0.78229	0.00	1.00	1.0E+00	1.00000
mmu-miR-574-5p	1.33	3.59	2.1E-01	0.78949	0.64	2.43	1.0E-01	0.40699
mmu-miR-181a	12.51	0.95	2.2E-01	0.80350	11.93	0.84	5.3E-02	0.27256
mmu-miR-128	5.97	0.63	2.2E-01	0.81702	5.34	1.16	5.7E-01	1.00000
mmu-miR-346	3.52	0.28	2.3E-01	0.82975	0.03	1.04	3.6E-01	0.73909
mmu-miR-455	5.87	0.73	2.3E-01	0.82975	5.35	0.83	3.9E-01	0.80367
mmu-miR-501-5p	3.73	1.23	2.3E-01	0.83208	2.52	4.89	6.7E-02	0.32520
mmu-miR-154	8.85	0.92	2.4E-01	0.83208	8.76	1.06	1.7E-01	0.55869
mmu-miR-369-3p	6.35	1.48	2.4E-01	0.83208	4.23	2.77	9.0E-02	0.38355
mmu-miR-33	5.65	0.81	2.4E-01	0.84014	5.79	1.13	2.8E-01	0.73909
mmu-miR-25	11.37	1.07	2.4E-01	0.84014	10.61	1.32	8.3E-03	0.07195
mmu-miR-450b-3p	2.90	0.34	2.5E-01	0.85454	1.24	0.19	1.1E-01	0.40852
mmu-miR-193	5.46	1.32	2.5E-01	0.85454	6.41	0.93	6.7E-01	1.00000
mmu-miR-691	3.73	0.25	2.5E-01	0.85533	5.12	0.40	5.8E-02	0.28601
mmu-miR-296-5p	8.11	0.92	2.5E-01	0.86411	6.37	1.09	6.4E-01	1.00000

mmu-miR-1955	7.67	1.13	2.6E-01	0.86411	7.79	1.07	3.1E-01	0.73909
mmu-miR-133a	2.93	0.35	2.6E-01	0.86991	1.30	1.58	7.4E-01	1.00000
mmu-miR-103	10.18	0.52	2.7E-01	0.86991	10.23	1.22	4.7E-02	0.24941
mmu-miR-1188	1.57	0.30	2.7E-01	0.86991	0.55	2.14	3.6E-01	0.73909
mmu-miR-331-5p	3.88	0.29	2.8E-01	0.86991	3.59	0.74	9.4E-02	0.38502
mmu-miR-361	8.61	0.87	2.8E-01	0.86991	8.24	1.00	9.7E-01	1.00000
mmu-miR-486	5.07	0.57	2.9E-01	0.86991	6.85	2.06	2.6E-01	0.73909
mmu-miR-500	1.48	3.05	2.9E-01	0.86991	1.93	5.50	5.6E-02	0.28248
mmu-miR-19a	10.77	1.05	2.9E-01	0.86991	10.06	1.49	5.0E-04	0.01219
mmu-miR-30d	11.11	0.91	3.1E-01	0.86991	11.55	0.94	2.8E-01	0.73909
mmu-miR-380-3p	2.13	2.89	3.1E-01	0.86991	0.52	2.05	3.6E-01	0.73909
mmu-miR-205	8.81	1.26	3.2E-01	0.86991	8.84	1.87	7.1E-02	0.33367
mmu-miR-466d-3p	3.61	0.35	3.3E-01	0.86991	3.69	2.15	1.3E-01	0.46039
mmu-miR-208b	4.23	1.50	3.4E-01	0.86991	4.32	0.84	2.3E-01	0.71079
mmu-miR-129-5p	1.13	0.48	3.4E-01	0.86991	0.00	1.00	1.0E+00	1.00000
mmu-miR-678	4.80	0.52	3.4E-01	0.86991	3.88	1.20	3.5E-01	0.73909
mmu-miR-501-3p	0.63	2.41	3.5E-01	0.86991	1.18	1.67	5.4E-01	1.00000
mmu-miR-1895	0.63	2.41	3.5E-01	0.86991	0.00	1.00	1.0E+00	1.00000
mmu-miR-137	0.59	0.44	3.5E-01	0.86991	0.00	1.00	1.0E+00	1.00000
mmu-miR-544	0.58	2.24	3.5E-01	0.86991	0.71	1.66	4.0E-01	0.81823
mmu-miR-1904	0.58	2.24	3.5E-01	0.86991	0.00	1.00	1.0E+00	1.00000
mmu-miR-212	0.54	2.12	3.5E-01	0.86991	1.21	5.34	1.0E-01	0.40699
mmu-miR-466d-5p	0.49	1.97	3.5E-01	0.86991	0.00	1.00	1.0E+00	1.00000
mmu-miR-487b	0.47	1.91	3.5E-01	0.86991	0.00	1.00	1.0E+00	1.00000
mmu-miR-1961	0.46	0.53	3.5E-01	0.86991	0.00	1.00	1.0E+00	1.00000
mmu-miR-105	0.41	1.77	3.5E-01	0.86991	0.00	1.00	1.0E+00	1.00000
mmu-miR-216a	0.41	1.77	3.5E-01	0.86991	0.00	1.00	1.0E+00	1.00000
mmu-miR-1	0.40	1.73	3.5E-01	0.86991	0.88	3.38	2.4E-01	0.72182
mmu-miR-1193	0.40	1.73	3.5E-01	0.86991	0.00	1.00	1.0E+00	1.00000
mmu-miR-876-5p	0.38	0.59	3.5E-01	0.86991	2.83	0.47	4.9E-01	0.98007
mmu-miR-880	0.38	0.59	3.5E-01	0.86991	0.15	1.23	3.6E-01	0.73909
mmu-miR-767	0.37	0.60	3.5E-01	0.86991	0.00	1.00	1.0E+00	1.00000
mmu-miR-337-5p	0.35	1.63	3.5E-01	0.86991	0.00	1.00	1.0E+00	1.00000
mmu-miR-342-5p	0.35	1.63	3.5E-01	0.86991	0.00	1.00	1.0E+00	1.00000
mmu-miR-1945	0.31	0.65	3.5E-01	0.86991	0.00	1.00	1.0E+00	1.00000
mmu-miR-1949	0.30	1.52	3.5E-01	0.86991	0.70	2.63	3.3E-01	0.73909
mmu-miR-1199	0.30	1.52	3.5E-01	0.86991	0.00	1.00	1.0E+00	1.00000
mmu-miR-669h-5p	0.23	1.38	3.5E-01	0.86991	0.52	2.05	1.1E-01	0.40841
mmu-miR-1943	0.23	1.38	3.5E-01	0.86991	0.57	2.20	3.6E-01	0.73909
mmu-miR-344	0.23	1.38	3.5E-01	0.86991	0.35	1.63	3.6E-01	0.73909
mmu-miR-3474	0.23	1.38	3.5E-01	0.86991	0.00	1.00	1.0E+00	1.00000
mmu-miR-466l	0.23	1.38	3.5E-01	0.86991	0.00	1.00	1.0E+00	1.00000
mmu-miR-2137	0.21	1.34	3.5E-01	0.86991	0.00	1.00	1.0E+00	1.00000
mmu-miR-874	0.17	0.79	3.5E-01	0.86991	0.55	0.47	3.6E-01	0.73909
mmu-miR-488	0.17	0.79	3.5E-01	0.86991	0.00	1.00	1.0E+00	1.00000
mmu-miR-743b-3p	0.17	0.79	3.5E-01	0.86991	0.00	1.00	1.0E+00	1.00000
mmu-miR-667	0.14	1.21	3.5E-01	0.86991	0.40	0.57	3.6E-01	0.73909
mmu-miR-1963	0.14	0.83	3.5E-01	0.86991	0.03	1.04	3.6E-01	0.73909
mmu-miR-301b	0.13	1.20	3.5E-01	0.86991	0.00	1.00	1.0E+00	1.00000
mmu-miR-449a	0.13	1.20	3.5E-01	0.86991	0.00	1.00	1.0E+00	1.00000
mmu-miR-491	0.13	1.20	3.5E-01	0.86991	0.00	1.00	1.0E+00	1.00000
mmu-miR-496	0.13	1.20	3.5E-01	0.86991	0.00	1.00	1.0E+00	1.00000
mmu-miR-688	0.13	1.20	3.5E-01	0.86991	0.00	1.00	1.0E+00	1.00000
mmu-miR-666-3p	0.09	0.88	3.5E-01	0.86991	0.00	1.00	1.0E+00	1.00000

mmu-miR-761	0.09	0.88	3.5E-01	0.86991	0.00	1.00	1.0E+00	1.00000
mmu-miR-1947	0.08	1.12	3.5E-01	0.86991	0.94	0.86	8.8E-01	1.00000
mmu-miR-1893	0.08	1.12	3.5E-01	0.86991	0.00	1.00	1.0E+00	1.00000
mmu-miR-290-3p	0.08	1.12	3.5E-01	0.86991	0.00	1.00	1.0E+00	1.00000
mmu-miR-3475	0.08	1.12	3.5E-01	0.86991	0.00	1.00	1.0E+00	1.00000
mmu-miR-684	0.08	1.12	3.5E-01	0.86991	0.00	1.00	1.0E+00	1.00000
mmu-miR-201	3.78	0.59	3.7E-01	0.90691	2.41	2.70	2.7E-01	0.73909
mmu-miR-19b	10.61	0.93	3.7E-01	0.90691	9.88	1.51	6.9E-05	0.00316
mmu-miR-130a	13.06	0.98	3.7E-01	0.90691	12.45	0.98	8.0E-01	1.00000
mmu-miR-709	3.56	0.38	3.8E-01	0.92808	4.35	1.51	7.3E-03	0.06637
mmu-miR-466a/b-3p	5.77	0.86	3.9E-01	0.93880	5.05	1.17	3.2E-01	0.73909
mmu-miR-741	4.83	1.24	3.9E-01	0.93926	4.79	0.53	1.3E-01	0.45196
mmu-miR-323-5p	1.23	2.17	3.9E-01	0.93995	0.00	1.00	1.0E+00	1.00000
mmu-miR-206	1.97	0.29	4.0E-01	0.94227	1.34	6.44	1.0E-02	0.08172
mmu-miR-106b	10.04	0.96	4.0E-01	0.94831	9.41	1.46	6.5E-04	0.01461
mmu-miR-423-3p	7.61	0.85	4.1E-01	0.95126	7.17	0.84	1.4E-01	0.50168
mmu-miR-532-3p	6.26	0.78	4.1E-01	0.95126	5.25	2.73	6.5E-03	0.06117
mmu-miR-7b	2.56	0.46	4.1E-01	0.95924	2.20	0.90	9.0E-01	1.00000
mmu-miR-582-5p	1.88	0.35	4.1E-01	0.95924	4.00	0.28	9.4E-02	0.38502
mmu-miR-423-5p	7.99	1.08	4.1E-01	0.95924	7.85	0.99	8.5E-01	1.00000
mmu-miR-547	1.23	1.97	4.2E-01	0.96182	2.89	1.06	8.4E-01	1.00000
mmu-miR-133b	0.99	0.58	4.2E-01	0.97489	0.11	0.86	3.6E-01	0.73909
mmu-miR-664	4.54	1.34	4.3E-01	0.99199	4.73	1.04	9.1E-01	1.00000
mmu-miR-497	7.64	0.92	4.4E-01	0.99811	7.58	1.78	9.6E-04	0.01716
mmu-miR-1224	5.52	1.39	4.4E-01	1.00000	5.58	2.85	7.0E-03	0.06488
mmu-miR-1929	3.31	2.24	4.5E-01	1.00000	3.21	1.80	3.2E-01	0.73909
mmu-miR-1903	1.54	2.20	4.5E-01	1.00000	0.00	1.00	1.0E+00	1.00000
mmu-miR-7a	1.91	0.37	4.6E-01	1.00000	0.78	2.96	8.1E-02	0.36446
mmu-miR-202-3p	0.46	0.66	4.6E-01	1.00000	0.34	1.61	3.6E-01	0.73909
mmu-miR-297c	5.83	0.76	4.6E-01	1.00000	5.41	1.27	3.9E-01	0.79461
mmu-miR-701	3.69	0.44	4.7E-01	1.00000	0.73	2.75	1.5E-01	0.50663
mmu-miR-122	3.64	3.66	4.7E-01	1.00000	5.29	1.33	8.0E-01	1.00000
mmu-miR-181b/d	6.96	0.86	4.8E-01	1.00000	5.81	1.27	1.7E-02	0.12535
mmu-miR-542-5p	5.44	0.81	4.8E-01	1.00000	3.73	0.83	5.3E-01	1.00000
mmu-miR-875-5p	1.39	1.95	4.8E-01	1.00000	0.48	1.95	3.6E-01	0.73909
mmu-miR-672	3.94	1.65	4.9E-01	1.00000	1.52	8.19	9.4E-04	0.01716
mmu-miR-1942	1.99	0.38	4.9E-01	1.00000	1.56	2.20	3.5E-01	0.73909
mmu-miR-101b	8.39	0.92	4.9E-01	1.00000	8.16	0.92	1.5E-01	0.50663
mmu-miR-193b	7.06	0.92	5.0E-01	1.00000	7.26	0.90	1.5E-01	0.50663
mmu-miR-136	8.59	1.11	5.1E-01	1.00000	7.63	1.32	3.9E-02	0.21620
mmu-miR-876-3p	1.36	2.08	5.1E-01	1.00000	4.58	0.34	1.3E-04	0.00449
mmu-miR-489	5.07	1.17	5.2E-01	1.00000	4.80	1.16	4.8E-01	0.98007
mmu-miR-1194	1.96	0.42	5.2E-01	1.00000	0.48	1.95	3.6E-01	0.73909
mmu-miR-505	1.82	0.48	5.2E-01	1.00000	0.00	1.00	1.0E+00	1.00000
mmu-miR-1983	4.43	1.19	5.2E-01	1.00000	0.22	0.74	3.6E-01	0.73909
mmu-miR-326	6.01	0.96	5.2E-01	1.00000	6.48	0.86	3.2E-01	0.73909
mmu-miR-1933-5p	1.48	2.04	5.3E-01	1.00000	3.68	0.78	5.5E-01	1.00000
mmu-miR-700	1.57	1.96	5.4E-01	1.00000	0.30	1.51	3.6E-01	0.73909
mmu-miR-2183	7.67	0.85	5.4E-01	1.00000	7.47	1.04	7.0E-01	1.00000
mmu-miR-665	1.39	1.80	5.4E-01	1.00000	0.00	1.00	1.0E+00	1.00000
mmu-miR-190	4.98	0.75	5.4E-01	1.00000	6.54	0.69	6.5E-02	0.32186
mmu-miR-98	10.30	0.95	5.6E-01	1.00000	10.28	0.87	4.6E-03	0.04893
mmu-miR-125b-3p	5.59	0.92	5.6E-01	1.00000	5.50	0.96	8.6E-01	1.00000
mmu-miR-541	3.29	1.85	5.6E-01	1.00000	0.67	2.52	7.1E-02	0.33367
mmu-miR-29c	9.04	0.97	5.7E-01	1.00000	11.17	0.69	1.6E-02	0.11999

mmu-miR-3472	1.98	0.54	5.7E-01	1.00000	0.46	1.88	1.0E-01	0.40699
mmu-miR-224	2.29	1.98	5.7E-01	1.00000	1.39	6.84	7.3E-04	0.01506
mmu-miR-1306	2.42	0.54	5.7E-01	1.00000	1.46	4.12	1.8E-02	0.12700
mmu-miR-92b	5.72	1.20	5.7E-01	1.00000	4.57	1.06	6.5E-01	1.00000
mmu-miR-713	0.77	0.62	5.8E-01	1.00000	0.76	1.79	3.6E-01	0.73966
mmu-miR-669a	4.63	0.79	5.8E-01	1.00000	3.91	1.52	2.8E-01	0.73909
mmu-miR-153	2.06	0.55	5.8E-01	1.00000	0.29	1.50	3.6E-01	0.73909
mmu-miR-539	5.26	0.79	5.9E-01	1.00000	4.34	1.69	8.8E-02	0.38299
mmu-miR-1900	2.78	1.63	6.0E-01	1.00000	0.29	1.50	3.6E-01	0.73909
mmu-miR-467f	4.39	0.59	6.0E-01	1.00000	4.19	2.04	4.6E-02	0.24881
mmu-miR-1892	0.79	0.64	6.0E-01	1.00000	0.00	1.00	1.0E+00	1.00000
mmu-miR-669o	2.36	0.64	6.1E-01	1.00000	3.43	1.76	2.2E-01	0.69037
mmu-miR-871	1.98	0.60	6.2E-01	1.00000	0.89	3.43	1.1E-01	0.41741
mmu-miR-1964	1.31	1.47	6.2E-01	1.00000	0.17	0.79	3.6E-01	0.73909
mmu-miR-340-3p	5.45	0.92	6.3E-01	1.00000	5.26	0.90	5.7E-01	1.00000
mmu-miR-362-3p	5.05	0.90	6.3E-01	1.00000	5.51	2.18	1.0E-02	0.08271
mmu-miR-2139	4.52	0.71	6.3E-01	1.00000	3.55	1.47	2.5E-01	0.73909
mmu-miR-882	1.64	1.72	6.4E-01	1.00000	0.00	1.00	1.0E+00	1.00000
mmu-miR-1839-3p	5.36	0.84	6.5E-01	1.00000	4.91	1.14	5.5E-01	1.00000
mmu-miR-429	12.49	0.97	6.5E-01	1.00000	12.24	0.94	1.6E-01	0.53314
mmu-miR-27b	8.10	1.02	6.6E-01	1.00000	8.16	0.87	3.3E-01	0.73909
mmu-miR-367	4.10	0.76	6.6E-01	1.00000	2.35	6.74	3.2E-02	0.18367
mmu-miR-421	7.01	0.92	6.6E-01	1.00000	6.29	1.13	9.6E-02	0.38909
mmu-miR-3473	3.38	1.33	6.7E-01	1.00000	1.02	4.09	2.6E-02	0.15930
mmu-miR-34b-3p	0.69	0.73	6.7E-01	1.00000	0.03	1.04	3.6E-01	0.73909
mmu-miR-879	0.69	0.73	6.7E-01	1.00000	0.00	1.00	1.0E+00	1.00000
mmu-miR-466g	6.52	1.04	6.7E-01	1.00000	6.38	1.03	8.4E-01	1.00000
mmu-miR-374	8.69	0.97	6.8E-01	1.00000	8.29	1.10	1.5E-01	0.50663
mmu-miR-432	2.11	0.62	6.8E-01	1.00000	0.00	1.00	1.0E+00	1.00000
mmu-miR-1981	5.83	1.09	6.9E-01	1.00000	6.06	1.02	9.3E-01	1.00000
mmu-miR-320	1.68	1.42	6.9E-01	1.00000	2.12	0.49	5.0E-01	1.00000
mmu-miR-542-3p	1.64	1.58	7.0E-01	1.00000	0.35	1.63	3.6E-01	0.73909
mmu-miR-674	5.71	1.10	7.0E-01	1.00000	5.06	1.28	8.1E-02	0.36446
mmu-miR-467c	2.60	1.32	7.0E-01	1.00000	3.77	1.36	2.8E-01	0.73909
mmu-miR-503	5.04	1.04	7.0E-01	1.00000	0.92	0.63	3.3E-01	0.73909
mmu-miR-490	1.00	0.67	7.0E-01	1.00000	0.00	1.00	1.0E+00	1.00000
mmu-miR-466k	3.64	0.69	7.0E-01	1.00000	0.84	1.19	8.3E-01	1.00000
mmu-miR-1894-5p	0.93	0.74	7.1E-01	1.00000	0.00	1.00	1.0E+00	1.00000
mmu-miR-125a-5p	13.19	0.99	7.1E-01	1.00000	12.35	1.15	6.2E-03	0.05986
mmu-miR-467e	5.40	0.91	7.2E-01	1.00000	4.81	1.66	1.1E-01	0.40818
mmu-miR-292-5p	1.04	1.47	7.2E-01	1.00000	2.21	1.06	9.6E-01	1.00000
mmu-miR-669j	0.71	1.24	7.2E-01	1.00000	0.23	1.37	3.6E-01	0.73909
mmu-miR-760	1.15	0.74	7.3E-01	1.00000	0.15	1.23	3.6E-01	0.73909
mmu-miR-1960	2.00	0.69	7.3E-01	1.00000	0.00	1.00	1.0E+00	1.00000
mmu-miR-1197	0.90	1.37	7.3E-01	1.00000	0.12	1.18	3.6E-01	0.73909
mmu-miR-151-3p	6.05	1.06	7.5E-01	1.00000	6.22	1.02	6.7E-01	1.00000
mmu-miR-99b	11.12	0.98	7.5E-01	1.00000	10.30	1.10	9.1E-02	0.38502
mmu-miR-149	5.46	1.06	7.5E-01	1.00000	4.91	0.48	8.9E-02	0.38299
mmu-miR-467h+mmu-miR-669d/l	0.55	0.84	7.6E-01	1.00000	0.15	1.23	3.6E-01	0.73909
mmu-miR-300	4.60	1.25	7.7E-01	1.00000	1.17	2.35	2.9E-01	0.73909
mmu-miR-669f	8.15	0.99	7.7E-01	1.00000	7.51	1.01	9.5E-01	1.00000
mmu-miR-155	6.98	0.85	7.7E-01	1.00000	7.41	1.00	9.8E-01	1.00000
mmu-miR-470	0.43	1.13	7.8E-01	1.00000	0.76	0.35	1.0E-01	0.40699
mmu-miR-298	2.78	1.31	7.8E-01	1.00000	1.04	1.97	3.4E-01	0.73909
mmu-miR-671-3p	1.97	1.33	7.8E-01	1.00000	0.23	1.37	3.6E-01	0.73909

mmu-miR-804	3.98	1.23	7.8E-01	1.00000	4.36	7.09	1.0E-02	0.08172
mmu-miR-467g	2.64	0.69	7.8E-01	1.00000	0.00	1.00	1.0E+00	1.00000
mmu-miR-323-3p	1.66	1.39	7.9E-01	1.00000	0.75	1.31	7.3E-01	1.00000
mmu-miR-485	0.78	1.16	7.9E-01	1.00000	0.00	1.00	1.0E+00	1.00000
mmu-miR-124	2.17	1.36	7.9E-01	1.00000	0.34	1.61	3.6E-01	0.73909
mmu-miR-652	7.13	1.03	8.0E-01	1.00000	6.56	1.15	2.5E-01	0.73027
mmu-miR-509-5p	1.70	0.74	8.0E-01	1.00000	0.00	1.00	1.0E+00	1.00000
mmu-miR-467a	4.81	0.93	8.1E-01	1.00000	4.53	1.72	1.8E-01	0.58413
mmu-miR-1941-5p	2.50	0.73	8.1E-01	1.00000	0.36	1.64	1.4E-01	0.49465
mmu-miR-146b	6.21	0.97	8.1E-01	1.00000	6.12	1.22	3.4E-01	0.73909
mmu-miR-152	10.84	1.01	8.2E-01	1.00000	10.43	1.06	2.4E-01	0.72508
mmu-miR-376a	8.85	0.92	8.2E-01	1.00000	8.39	1.15	3.9E-01	0.79841
mmu-miR-463	1.42	0.84	8.3E-01	1.00000	0.23	1.37	3.6E-01	0.73909
mmu-miR-129-3p	1.03	0.80	8.3E-01	1.00000	0.43	1.81	3.1E-01	0.73909
mmu-miR-883b-3p	4.97	1.06	8.4E-01	1.00000	4.12	0.97	9.1E-01	1.00000
mmu-miR-338-5p	2.63	0.83	8.4E-01	1.00000	2.07	0.63	6.0E-01	1.00000
mmu-miR-770-5p	1.45	0.77	8.5E-01	1.00000	0.00	1.00	1.0E+00	1.00000
mmu-miR-1953	2.65	1.30	8.5E-01	1.00000	3.60	0.65	2.9E-01	0.73909
mmu-miR-1956	2.12	0.82	8.5E-01	1.00000	0.45	1.85	3.6E-01	0.73909
mmu-miR-1192	0.78	0.88	8.5E-01	1.00000	0.14	0.83	3.6E-01	0.73909
mmu-let-7c	14.41	1.00	8.6E-01	1.00000	14.77	1.04	3.5E-01	0.73909
mmu-miR-1957	2.36	0.80	8.6E-01	1.00000	0.00	1.00	1.0E+00	1.00000
mmu-miR-142-5p	5.94	1.03	8.6E-01	1.00000	6.66	0.91	6.1E-01	1.00000
mmu-miR-15b	11.80	1.02	8.6E-01	1.00000	11.10	1.71	2.5E-03	0.03063
mmu-miR-125b-5p	13.88	0.99	8.7E-01	1.00000	13.72	1.14	2.7E-02	0.16268
mmu-miR-710	2.09	0.84	8.7E-01	1.00000	0.23	1.37	3.6E-01	0.73909
mmu-miR-1928	2.74	0.80	8.7E-01	1.00000	0.94	2.00	2.3E-01	0.69608
mmu-miR-27a	12.17	1.01	8.8E-01	1.00000	12.05	1.27	9.5E-03	0.08002
mmu-miR-875-3p	1.77	1.15	8.8E-01	1.00000	0.14	0.83	3.6E-01	0.73909
mmu-miR-369-5p	2.88	0.85	8.8E-01	1.00000	0.23	1.38	3.6E-01	0.73909
mmu-miR-675-5p	2.66	0.86	8.9E-01	1.00000	0.45	1.85	3.6E-01	0.73909
mmu-miR-668	0.88	0.88	8.9E-01	1.00000	0.00	1.00	1.0E+00	1.00000
mmu-miR-483	1.78	1.18	8.9E-01	1.00000	0.00	1.00	1.0E+00	1.00000
mmu-miR-1946a	2.65	0.89	8.9E-01	1.00000	0.37	0.87	7.1E-01	1.00000
mmu-miR-32	8.90	1.01	8.9E-01	1.00000	9.07	0.87	8.4E-02	0.37106
mmu-miR-34b-5p	2.22	1.18	8.9E-01	1.00000	3.07	1.65	3.5E-02	0.19756
mmu-miR-381	3.63	1.13	9.0E-01	1.00000	0.24	1.39	3.6E-01	0.73909
mmu-miR-1958	1.89	1.14	9.0E-01	1.00000	0.00	1.00	1.0E+00	1.00000
mmu-miR-683	2.41	0.86	9.1E-01	1.00000	0.00	1.00	1.0E+00	1.00000
mmu-miR-883b-5p	1.47	1.09	9.1E-01	1.00000	0.00	1.00	1.0E+00	1.00000
mmu-miR-1195	2.32	1.14	9.1E-01	1.00000	0.30	1.51	3.6E-01	0.73909
mmu-miR-1931	0.64	1.07	9.2E-01	1.00000	0.00	1.00	1.0E+00	1.00000
mmu-miR-883a-3p	0.78	0.93	9.3E-01	1.00000	0.00	1.00	1.0E+00	1.00000
mmu-miR-1902	7.56	1.02	9.3E-01	1.00000	8.24	1.51	5.8E-03	0.05792
mmu-miR-764-3p	3.25	0.88	9.4E-01	1.00000	2.48	4.42	3.7E-02	0.20428
mmu-miR-764-5p	2.35	1.09	9.5E-01	1.00000	1.08	1.29	7.6E-01	1.00000
mmu-miR-669i	2.26	0.93	9.5E-01	1.00000	1.68	0.28	2.4E-01	0.72198
mmu-miR-203	8.53	1.00	9.6E-01	1.00000	8.59	0.86	2.5E-01	0.73909
mmu-miR-3470a/b	1.13	0.95	9.6E-01	1.00000	0.00	1.00	1.0E+00	1.00000
mmu-miR-330	2.06	1.05	9.6E-01	1.00000	0.00	1.00	1.0E+00	1.00000
mmu-miR-670	2.06	0.95	9.6E-01	1.00000	2.40	1.96	3.7E-01	0.76569
mmu-let-7e	12.18	1.00	9.7E-01	1.00000	11.64	1.10	1.3E-01	0.45196
mmu-miR-184	3.12	1.06	9.7E-01	1.00000	1.00	3.99	1.1E-01	0.41741
mmu-miR-877	2.86	0.97	9.8E-01	1.00000	1.13	4.76	1.0E-01	0.40699
mmu-miR-365	9.53	1.00	9.8E-01	1.00000	9.96	0.86	1.8E-01	0.57529

mmu-miR-654-3p	2.61	0.98	9.9E-01	1.00000	0.40	1.75	7.1E-05	0.00316
mmu-miR-363	0.00	1.00	1.0E+00	1.00000	0.91	0.29	1.1E-01	0.40841
mmu-miR-337-3p	0.00	1.00	1.0E+00	1.00000	0.33	1.57	3.0E-01	0.73909
mmu-miR-467b	0.00	1.00	1.0E+00	1.00000	0.60	0.43	3.6E-01	0.73909
mmu-miR-1191	0.00	1.00	1.0E+00	1.00000	0.40	0.57	3.6E-01	0.73909
mmu-miR-302d	0.00	1.00	1.0E+00	1.00000	0.40	0.57	3.6E-01	0.73909
mmu-miR-743b-5p	0.00	1.00	1.0E+00	1.00000	0.40	0.57	3.6E-01	0.73909
mmu-miR-219	0.00	1.00	1.0E+00	1.00000	0.23	1.38	3.6E-01	0.73909
mmu-miR-292-3p	0.00	1.00	1.0E+00	1.00000	0.15	1.23	3.6E-01	0.73909
mmu-miR-1982	0.00	1.00	1.0E+00	1.00000	0.14	0.83	3.6E-01	0.73909
mmu-miR-1901	0.00	1.00	1.0E+00	1.00000	0.12	1.18	3.6E-01	0.73909
mmu-miR-465b-5p	0.00	1.00	1.0E+00	1.00000	0.11	0.86	3.6E-01	0.73909
mmu-miR-742	0.00	1.00	1.0E+00	1.00000	0.11	0.86	3.6E-01	0.73909
mmu-miR-92a	0.00	1.00	1.0E+00	1.00000	0.03	1.04	3.6E-01	0.73909
mmu-miR-450b-5p	0.00	1.00	1.0E+00	1.00000	0.94	1.40	7.3E-01	1.00000
mmu-miR-546	0.00	1.00	1.0E+00	1.00000	0.23	1.02	9.3E-01	1.00000
mmu-miR-1186	0.00	1.00	1.0E+00	1.00000	0.00	1.00	1.0E+00	1.00000
mmu-miR-1190	0.00	1.00	1.0E+00	1.00000	0.00	1.00	1.0E+00	1.00000
mmu-miR-135b	0.00	1.00	1.0E+00	1.00000	0.00	1.00	1.0E+00	1.00000
mmu-miR-139-3p	0.00	1.00	1.0E+00	1.00000	0.00	1.00	1.0E+00	1.00000
mmu-miR-147	0.00	1.00	1.0E+00	1.00000	0.00	1.00	1.0E+00	1.00000
mmu-miR-186	0.00	1.00	1.0E+00	1.00000	0.00	1.00	1.0E+00	1.00000
mmu-miR-188-3p	0.00	1.00	1.0E+00	1.00000	0.00	1.00	1.0E+00	1.00000
mmu-miR-1894-3p	0.00	1.00	1.0E+00	1.00000	0.00	1.00	1.0E+00	1.00000
mmu-miR-1896	0.00	1.00	1.0E+00	1.00000	0.00	1.00	1.0E+00	1.00000
mmu-miR-1897-3p	0.00	1.00	1.0E+00	1.00000	0.00	1.00	1.0E+00	1.00000
mmu-miR-1898	0.00	1.00	1.0E+00	1.00000	0.00	1.00	1.0E+00	1.00000
mmu-miR-1899	0.00	1.00	1.0E+00	1.00000	0.00	1.00	1.0E+00	1.00000
mmu-miR-18b	0.00	1.00	1.0E+00	1.00000	0.00	1.00	1.0E+00	1.00000
mmu-miR-1905	0.00	1.00	1.0E+00	1.00000	0.00	1.00	1.0E+00	1.00000
mmu-miR-1906	0.00	1.00	1.0E+00	1.00000	0.00	1.00	1.0E+00	1.00000
mmu-miR-1907	0.00	1.00	1.0E+00	1.00000	0.00	1.00	1.0E+00	1.00000
mmu-miR-1927	0.00	1.00	1.0E+00	1.00000	0.00	1.00	1.0E+00	1.00000
mmu-miR-1930	0.00	1.00	1.0E+00	1.00000	0.00	1.00	1.0E+00	1.00000
mmu-miR-1932	0.00	1.00	1.0E+00	1.00000	0.00	1.00	1.0E+00	1.00000
mmu-miR-1933-3p	0.00	1.00	1.0E+00	1.00000	0.00	1.00	1.0E+00	1.00000
mmu-miR-1934	0.00	1.00	1.0E+00	1.00000	0.00	1.00	1.0E+00	1.00000
mmu-miR-1936	0.00	1.00	1.0E+00	1.00000	0.00	1.00	1.0E+00	1.00000
mmu-miR-1938	0.00	1.00	1.0E+00	1.00000	0.00	1.00	1.0E+00	1.00000
mmu-miR-1946b	0.00	1.00	1.0E+00	1.00000	0.00	1.00	1.0E+00	1.00000
mmu-miR-1948	0.00	1.00	1.0E+00	1.00000	0.00	1.00	1.0E+00	1.00000
mmu-miR-1950	0.00	1.00	1.0E+00	1.00000	0.00	1.00	1.0E+00	1.00000
mmu-miR-1952	0.00	1.00	1.0E+00	1.00000	0.00	1.00	1.0E+00	1.00000
mmu-miR-1954	0.00	1.00	1.0E+00	1.00000	0.00	1.00	1.0E+00	1.00000
mmu-miR-1962	0.00	1.00	1.0E+00	1.00000	0.00	1.00	1.0E+00	1.00000
mmu-miR-1966	0.00	1.00	1.0E+00	1.00000	0.00	1.00	1.0E+00	1.00000
mmu-miR-1967	0.00	1.00	1.0E+00	1.00000	0.00	1.00	1.0E+00	1.00000
mmu-miR-1968	0.00	1.00	1.0E+00	1.00000	0.00	1.00	1.0E+00	1.00000
mmu-miR-1969	0.00	1.00	1.0E+00	1.00000	0.00	1.00	1.0E+00	1.00000
mmu-miR-1970	0.00	1.00	1.0E+00	1.00000	0.00	1.00	1.0E+00	1.00000
mmu-miR-1971	0.00	1.00	1.0E+00	1.00000	0.00	1.00	1.0E+00	1.00000
mmu-miR-207	0.00	1.00	1.0E+00	1.00000	0.00	1.00	1.0E+00	1.00000
mmu-miR-208a	0.00	1.00	1.0E+00	1.00000	0.00	1.00	1.0E+00	1.00000
mmu-miR-211	0.00	1.00	1.0E+00	1.00000	0.00	1.00	1.0E+00	1.00000
mmu-miR-2136	0.00	1.00	1.0E+00	1.00000	0.00	1.00	1.0E+00	1.00000

mmu-miR-216b	0.00	1.00	1.0E+00	1.00000	0.00	1.00	1.0E+00	1.00000
mmu-miR-217	0.00	1.00	1.0E+00	1.00000	0.00	1.00	1.0E+00	1.00000
mmu-miR-2861	0.00	1.00	1.0E+00	1.00000	0.00	1.00	1.0E+00	1.00000
mmu-miR-291a-3p	0.00	1.00	1.0E+00	1.00000	0.00	1.00	1.0E+00	1.00000
mmu-miR-291a-5p	0.00	1.00	1.0E+00	1.00000	0.00	1.00	1.0E+00	1.00000
mmu-miR-291b-3p	0.00	1.00	1.0E+00	1.00000	0.00	1.00	1.0E+00	1.00000
mmu-miR-291b-5p	0.00	1.00	1.0E+00	1.00000	0.00	1.00	1.0E+00	1.00000
mmu-miR-293	0.00	1.00	1.0E+00	1.00000	0.00	1.00	1.0E+00	1.00000
mmu-miR-294	0.00	1.00	1.0E+00	1.00000	0.00	1.00	1.0E+00	1.00000
mmu-miR-295	0.00	1.00	1.0E+00	1.00000	0.00	1.00	1.0E+00	1.00000
mmu-miR-296-3p	0.00	1.00	1.0E+00	1.00000	0.00	1.00	1.0E+00	1.00000
mmu-miR-297b-3p	0.00	1.00	1.0E+00	1.00000	0.00	1.00	1.0E+00	1.00000
mmu-miR-297b-5p	0.00	1.00	1.0E+00	1.00000	0.00	1.00	1.0E+00	1.00000
mmu-miR-299	0.00	1.00	1.0E+00	1.00000	0.00	1.00	1.0E+00	1.00000
mmu-miR-302a	0.00	1.00	1.0E+00	1.00000	0.00	1.00	1.0E+00	1.00000
mmu-miR-302b	0.00	1.00	1.0E+00	1.00000	0.00	1.00	1.0E+00	1.00000
mmu-miR-302c	0.00	1.00	1.0E+00	1.00000	0.00	1.00	1.0E+00	1.00000
mmu-miR-3072	0.00	1.00	1.0E+00	1.00000	0.00	1.00	1.0E+00	1.00000
mmu-miR-3099	0.00	1.00	1.0E+00	1.00000	0.00	1.00	1.0E+00	1.00000
mmu-miR-324-3p	0.00	1.00	1.0E+00	1.00000	0.00	1.00	1.0E+00	1.00000
mmu-miR-325	0.00	1.00	1.0E+00	1.00000	0.00	1.00	1.0E+00	1.00000
mmu-miR-327	0.00	1.00	1.0E+00	1.00000	0.00	1.00	1.0E+00	1.00000
mmu-miR-339-3p	0.00	1.00	1.0E+00	1.00000	0.00	1.00	1.0E+00	1.00000
mmu-miR-339-5p	0.00	1.00	1.0E+00	1.00000	0.00	1.00	1.0E+00	1.00000
mmu-miR-341	0.00	1.00	1.0E+00	1.00000	0.00	1.00	1.0E+00	1.00000
mmu-miR-343	0.00	1.00	1.0E+00	1.00000	0.00	1.00	1.0E+00	1.00000
mmu-miR-345-3p	0.00	1.00	1.0E+00	1.00000	0.00	1.00	1.0E+00	1.00000
mmu-miR-34c	0.00	1.00	1.0E+00	1.00000	0.00	1.00	1.0E+00	1.00000
mmu-miR-370	0.00	1.00	1.0E+00	1.00000	0.00	1.00	1.0E+00	1.00000
mmu-miR-380-5p	0.00	1.00	1.0E+00	1.00000	0.00	1.00	1.0E+00	1.00000
mmu-miR-383	0.00	1.00	1.0E+00	1.00000	0.00	1.00	1.0E+00	1.00000
mmu-miR-384-3p	0.00	1.00	1.0E+00	1.00000	0.00	1.00	1.0E+00	1.00000
mmu-miR-384-5p	0.00	1.00	1.0E+00	1.00000	0.00	1.00	1.0E+00	1.00000
mmu-miR-409-3p	0.00	1.00	1.0E+00	1.00000	0.00	1.00	1.0E+00	1.00000
mmu-miR-448	0.00	1.00	1.0E+00	1.00000	0.00	1.00	1.0E+00	1.00000
mmu-miR-449b	0.00	1.00	1.0E+00	1.00000	0.00	1.00	1.0E+00	1.00000
mmu-miR-449c	0.00	1.00	1.0E+00	1.00000	0.00	1.00	1.0E+00	1.00000
mmu-miR-450a-3p	0.00	1.00	1.0E+00	1.00000	0.00	1.00	1.0E+00	1.00000
mmu-miR-452	0.00	1.00	1.0E+00	1.00000	0.00	1.00	1.0E+00	1.00000
mmu-miR-453	0.00	1.00	1.0E+00	1.00000	0.00	1.00	1.0E+00	1.00000
mmu-miR-465a-3p	0.00	1.00	1.0E+00	1.00000	0.00	1.00	1.0E+00	1.00000
mmu-miR-465a-5p	0.00	1.00	1.0E+00	1.00000	0.00	1.00	1.0E+00	1.00000
mmu-miR-465c-5p	0.00	1.00	1.0E+00	1.00000	0.00	1.00	1.0E+00	1.00000
mmu-miR-466a/e-5p	0.00	1.00	1.0E+00	1.00000	0.00	1.00	1.0E+00	1.00000
mmu-miR-466f-5p	0.00	1.00	1.0E+00	1.00000	0.00	1.00	1.0E+00	1.00000
mmu-miR-466h	0.00	1.00	1.0E+00	1.00000	0.00	1.00	1.0E+00	1.00000
mmu-miR-466i	0.00	1.00	1.0E+00	1.00000	0.00	1.00	1.0E+00	1.00000
mmu-miR-466j	0.00	1.00	1.0E+00	1.00000	0.00	1.00	1.0E+00	1.00000
mmu-miR-467d	0.00	1.00	1.0E+00	1.00000	0.00	1.00	1.0E+00	1.00000
mmu-miR-468	0.00	1.00	1.0E+00	1.00000	0.00	1.00	1.0E+00	1.00000
mmu-miR-471	0.00	1.00	1.0E+00	1.00000	0.00	1.00	1.0E+00	1.00000
mmu-miR-494	0.00	1.00	1.0E+00	1.00000	0.00	1.00	1.0E+00	1.00000
mmu-miR-499	0.00	1.00	1.0E+00	1.00000	0.00	1.00	1.0E+00	1.00000
mmu-miR-504	0.00	1.00	1.0E+00	1.00000	0.00	1.00	1.0E+00	1.00000
mmu-miR-509-3p	0.00	1.00	1.0E+00	1.00000	0.00	1.00	1.0E+00	1.00000

mmu-miR-511	0.00	1.00	1.0E+00	1.00000	0.00	1.00	1.0E+00	1.00000
mmu-miR-540-3p	0.00	1.00	1.0E+00	1.00000	0.00	1.00	1.0E+00	1.00000
mmu-miR-540-5p	0.00	1.00	1.0E+00	1.00000	0.00	1.00	1.0E+00	1.00000
mmu-miR-543	0.00	1.00	1.0E+00	1.00000	0.00	1.00	1.0E+00	1.00000
mmu-miR-568	0.00	1.00	1.0E+00	1.00000	0.00	1.00	1.0E+00	1.00000
mmu-miR-582-3p	0.00	1.00	1.0E+00	1.00000	0.00	1.00	1.0E+00	1.00000
mmu-miR-590-3p	0.00	1.00	1.0E+00	1.00000	0.00	1.00	1.0E+00	1.00000
mmu-miR-590-5p	0.00	1.00	1.0E+00	1.00000	0.00	1.00	1.0E+00	1.00000
mmu-miR-592	0.00	1.00	1.0E+00	1.00000	0.00	1.00	1.0E+00	1.00000
mmu-miR-599	0.00	1.00	1.0E+00	1.00000	0.00	1.00	1.0E+00	1.00000
mmu-miR-615-5p	0.00	1.00	1.0E+00	1.00000	0.00	1.00	1.0E+00	1.00000
mmu-miR-653	0.00	1.00	1.0E+00	1.00000	0.00	1.00	1.0E+00	1.00000
mmu-miR-654-5p	0.00	1.00	1.0E+00	1.00000	0.00	1.00	1.0E+00	1.00000
mmu-miR-666-5p	0.00	1.00	1.0E+00	1.00000	0.00	1.00	1.0E+00	1.00000
mmu-miR-669g	0.00	1.00	1.0E+00	1.00000	0.00	1.00	1.0E+00	1.00000
mmu-miR-669m	0.00	1.00	1.0E+00	1.00000	0.00	1.00	1.0E+00	1.00000
mmu-miR-671-5p	0.00	1.00	1.0E+00	1.00000	0.00	1.00	1.0E+00	1.00000
mmu-miR-673-3p	0.00	1.00	1.0E+00	1.00000	0.00	1.00	1.0E+00	1.00000
mmu-miR-673-5p	0.00	1.00	1.0E+00	1.00000	0.00	1.00	1.0E+00	1.00000
mmu-miR-675-3p	0.00	1.00	1.0E+00	1.00000	0.00	1.00	1.0E+00	1.00000
mmu-miR-677	0.00	1.00	1.0E+00	1.00000	0.00	1.00	1.0E+00	1.00000
mmu-miR-679	0.00	1.00	1.0E+00	1.00000	0.00	1.00	1.0E+00	1.00000
mmu-miR-680	0.00	1.00	1.0E+00	1.00000	0.00	1.00	1.0E+00	1.00000
mmu-miR-681	0.00	1.00	1.0E+00	1.00000	0.00	1.00	1.0E+00	1.00000
mmu-miR-682	0.00	1.00	1.0E+00	1.00000	0.00	1.00	1.0E+00	1.00000
mmu-miR-686	0.00	1.00	1.0E+00	1.00000	0.00	1.00	1.0E+00	1.00000
mmu-miR-687	0.00	1.00	1.0E+00	1.00000	0.00	1.00	1.0E+00	1.00000
mmu-miR-692	0.00	1.00	1.0E+00	1.00000	0.00	1.00	1.0E+00	1.00000
mmu-miR-693-3p	0.00	1.00	1.0E+00	1.00000	0.00	1.00	1.0E+00	1.00000
mmu-miR-693-5p	0.00	1.00	1.0E+00	1.00000	0.00	1.00	1.0E+00	1.00000
mmu-miR-694	0.00	1.00	1.0E+00	1.00000	0.00	1.00	1.0E+00	1.00000
mmu-miR-696	0.00	1.00	1.0E+00	1.00000	0.00	1.00	1.0E+00	1.00000
mmu-miR-697	0.00	1.00	1.0E+00	1.00000	0.00	1.00	1.0E+00	1.00000
mmu-miR-698	0.00	1.00	1.0E+00	1.00000	0.00	1.00	1.0E+00	1.00000
mmu-miR-702	0.00	1.00	1.0E+00	1.00000	0.00	1.00	1.0E+00	1.00000
mmu-miR-703	0.00	1.00	1.0E+00	1.00000	0.00	1.00	1.0E+00	1.00000
mmu-miR-704	0.00	1.00	1.0E+00	1.00000	0.00	1.00	1.0E+00	1.00000
mmu-miR-706	0.00	1.00	1.0E+00	1.00000	0.00	1.00	1.0E+00	1.00000
mmu-miR-707	0.00	1.00	1.0E+00	1.00000	0.00	1.00	1.0E+00	1.00000
mmu-miR-711	0.00	1.00	1.0E+00	1.00000	0.00	1.00	1.0E+00	1.00000
mmu-miR-712	0.00	1.00	1.0E+00	1.00000	0.00	1.00	1.0E+00	1.00000
mmu-miR-718	0.00	1.00	1.0E+00	1.00000	0.00	1.00	1.0E+00	1.00000
mmu-miR-719	0.00	1.00	1.0E+00	1.00000	0.00	1.00	1.0E+00	1.00000
mmu-miR-744	0.00	1.00	1.0E+00	1.00000	0.00	1.00	1.0E+00	1.00000
mmu-miR-758	0.00	1.00	1.0E+00	1.00000	0.00	1.00	1.0E+00	1.00000
mmu-miR-763	0.00	1.00	1.0E+00	1.00000	0.00	1.00	1.0E+00	1.00000
mmu-miR-878-3p	0.00	1.00	1.0E+00	1.00000	0.00	1.00	1.0E+00	1.00000
mmu-miR-878-5p	0.00	1.00	1.0E+00	1.00000	0.00	1.00	1.0E+00	1.00000
mmu-miR-881	0.00	1.00	1.0E+00	1.00000	0.00	1.00	1.0E+00	1.00000
mmu-miR-883a-5p	0.00	1.00	1.0E+00	1.00000	0.00	1.00	1.0E+00	1.00000

Supplementary Table 2:Commonly Dysregulated miRNAs in ADPKD models.

UP	DOWN
mmu-miR-182	mmu-let-7g
mmu-miR-183	mmu-miR-99a
mmu-miR-96	mmu-miR-126-3p
mmu-miR-21	mmu-miR-151-5p
mmu-miR-223	mmu-miR-196a
mmu-miR-199a-3p	mmu-miR-26b
mmu-miR-199a-5p	mmu-miR-126-5p
mmu-miR-200c	mmu-miR-30c
mmu-miR-100	mmu-miR-185
mmu-miR-350	mmu-miR-30e
mmu-let-7i	mmu-miR-194
mmu-miR-20a+mmu-miR-20b	mmu-miR-378
mmu-miR-28	mmu-miR-192
mmu-miR-106a+mmu-miR-17	
mmu-miR-532-5p	
mmu-miR-143	

Supplementary Table 3: CT values of polysome shift assay (PSA)

	High Molecular Weight Fraction 3				High Molecular Weight Fraction 4				Kidney PSA Score
	miR-17	Let-7d			miR-17	Let-7d			
Treatment	Ct Mean	Ct Mean	dCT	ddCT	Ct Mean	Ct Mean	dCT	ddCT	Average ddCT Fraction 3+4
PBS	24.613	22.832	1.781	0.640	25.404	24.220	1.184	-0.137	0.252
PBS	25.247	23.898	1.349	0.208	27.382	25.194	2.188	0.867	0.538
PBS	27.098	26.479	0.619	-0.522	25.233	23.393	1.840	0.519	-0.002
PBS	23.410	23.181	0.229	-0.912	25.116	24.855	0.260	-1.060	-0.986
PBS	25.474	24.601	0.873	-0.268	24.983	23.328	1.655	0.334	0.033
PBS	22.794	20.873	1.921	0.780	23.928	22.618	1.310	-0.011	0.385
PBS	23.936	23.103	0.833	-0.308	23.627	23.123	0.504	-0.816	-0.562
PBS	25.803	24.278	1.525	0.384	26.031	24.406	1.624	0.304	0.344
anti-miR-17 (0.3mg/kg)	25.169	23.098	2.071	0.930	26.489	24.627	1.862	0.541	0.736
anti-miR-17 (0.3mg/kg)	27.501	24.513	2.989	1.848	29.031	26.428	2.602	1.281	1.565
anti-miR-17 (0.3mg/kg)	26.641	23.342	3.299	2.158	28.544	25.404	3.140	1.819	1.988
anti-miR-17 (0.3mg/kg)	25.899	22.757	3.143	2.002	27.059	23.991	3.068	1.747	1.875
anti-miR-17 (0.3mg/kg)	26.012	23.630	2.383	1.242	26.383	23.793	2.590	1.270	1.256
anti-miR-17 (3mg/kg)	26.039	22.736	3.303	2.162	27.467	23.762	3.705	2.384	2.273
anti-miR-17 (3mg/kg)	27.601	23.845	3.756	2.615	28.783	25.172	3.611	2.290	2.452
anti-miR-17 (3mg/kg)	27.489	23.214	4.275	3.134	29.416	25.273	4.144	2.823	2.978
anti-miR-17 (3mg/kg)	26.003	23.578	2.426	1.285	26.320	24.203	2.118	0.797	1.041
anti-miR-17 (3mg/kg)	25.804	23.541	2.262	1.121	26.667	23.685	2.982	1.661	1.391
anti-miR-17 (30mg/kg)	27.129	22.542	4.587	3.446	28.124	24.369	3.755	2.435	2.940
anti-miR-17 (30mg/kg)	28.382	23.869	4.513	3.372	28.797	24.674	4.123	2.802	3.087
anti-miR-17 (30mg/kg)	28.627	24.316	4.310	3.169	30.112	25.228	4.884	3.563	3.366
anti-miR-17 (30mg/kg)	26.441	23.118	3.323	2.182	26.265	22.993	3.272	1.951	2.066
anti-miR-17 (30mg/kg)	26.224	23.451	2.773	1.632	26.517	22.278	4.239	2.919	2.275
Control (30mg/kg)	24.123	22.582	1.542	0.401	26.319	25.186	1.133	-0.188	0.106
Control (30mg/kg)	25.270	24.040	1.230	0.089	27.691	26.223	1.467	0.147	0.118
Control (30mg/kg)	26.845	27.007	-0.161	-1.302	25.646	23.635	2.011	0.690	-0.306
Control (30mg/kg)	23.612	23.821	-0.210	-1.351	26.293	24.911	1.382	0.062	-0.644
Control (30mg/kg)	25.547	23.762	1.785	0.644	25.120	23.398	1.723	0.402	0.523
Control (30mg/kg)	26.598	25.099	1.499	0.358	26.946	25.398	1.549	0.228	0.293
Control (30mg/kg)	23.957	21.751	2.205	1.064	25.466	23.964	1.502	0.181	0.623
Control (30mg/kg)	25.330	23.686	1.644	0.503	25.088	24.201	0.886	-0.434	0.035

Supplementary Table 4: Differential expression of miR-17~92 putative targets (TargetScan) in *PKD1* $^{F/RC}$ mutant kidneys (DKO vs SKO)

miR-17

Confidence	Gene	Fold Change
High (predicted)	A1cf	2.858
High (predicted)	Ahrr	2.241
High (predicted)	Ak4	4.305
High (predicted)	Ankrd29	1.860
High (predicted)	Arl4a	1.722
High (predicted)	Bcl11b	1.880
High (predicted)	Bnc2	1.773
High (predicted)	Cd274	1.939
High (predicted)	Cdc37l1	2.079
High (predicted)	Cers6	1.720
High (predicted)	Cpeb3	2.224
High (predicted)	Cript	1.887
High (predicted)	Csrnp3	2.493
High (predicted)	Dab2	1.632
High (predicted)	Dennd5b	1.729
High (predicted)	Dlgap1	2.753
High (predicted)	Dnajb6	1.803
High (predicted)	Dnajb9	1.772
High (predicted)	Ehd3	2.392
High (predicted)	Enpp5	1.722
High (predicted)	Epha5	15.074
High (predicted)	Epha7	1.813
High (predicted)	Fam210a	1.866
High (predicted)	Foxj3	1.753
High (predicted)	Fyco1	2.544
High (predicted)	Gpr137b	2.815
High (predicted)	Hlf	2.523
High (predicted)	Itfg1	2.010
High (predicted)	Itgb8	1.916
High (predicted)	Lace1	2.014
High (predicted)	Ldlrad3	1.639
High (predicted)	Map3k5	1.609
High (predicted)	Mfap3l	1.788
High (predicted)	Mier3	1.865
High (predicted)	Nabp1	2.648
High (predicted)	Napepld	2.144
High (predicted)	Nceh1	1.657
High (predicted)	Ndel1	1.556
High (predicted)	Neurod2	17.606
High (predicted)	Nhs	1.983
High (predicted)	Ntrk3	3.167
High (predicted)	Osr1	3.829
Experimentally Observed	Pak7	3.392
High (predicted)	Pex5l	2.158
High (predicted)	Plag1	2.157
High (predicted)	Pls1	1.671
High (predicted)	Plscr4	1.789
High (predicted)	Polr3g	1.744
High (predicted)	Ppara	1.982
Experimentally Observed	Pparg	2.836
High (predicted)	Ppp1r3b	2.807
High (predicted)	Ppp2ca	1.666
High (predicted)	Ppp6c	1.604
High (predicted)	Ptger3	4.234
High (predicted)	Ptpro	2.043
High (predicted)	Ranbp6	1.717
High (predicted)	Sar1b	1.811
Moderate (predicted)	Slc12a3	3.432
High (predicted)	Slc16a9	2.599
High (predicted)	Slc1a2	2.509
High (predicted)	Slc24a2	10.098
High (predicted)	Slc24a4	9.952
High (predicted)	Slc25a36	1.751
High (predicted)	Slc33a1	1.701
High (predicted)	Slc4a4	2.772
High (predicted)	Slc5a3	2.283
High (predicted)	St8sia3	11.680
High (predicted)	Thrb	1.924
High (predicted)	Timm17a	1.679
High (predicted)	Tmem100	3.623
High (predicted)	Tmem64	1.739
High (predicted)	Tnfrsf21	1.729
High (predicted)	Trabd2b	1.921
High (predicted)	Trps1	1.811
High (predicted)	Ttc39c	1.772
High (predicted)	Ube2ql1	2.553
High (predicted)	Ucp3	5.959
High (predicted)	Usp3	1.621
High (predicted)	Vldlr	1.852
High (predicted)	Wac	1.670
High (predicted)	Wnk1	2.338

miR-18a

Confidence	Gene	Fold Change
High (predicted)	Csrnp3	2.493
High (predicted)	Fam166b	2.349
High (predicted)	Fgf1	1.576
High (predicted)	Hlf	2.523
High (predicted)	Kcna1	4.451
High (predicted)	Rab9	1.880
High (predicted)	Robo2	2.641
High (predicted)	Slc12a6	1.838
High (predicted)	Slc1a2	2.509
High (predicted)	Trib2	1.587
High (predicted)	Uqcrb	4.830

miR-19b

Confidence	Gene	Fold Change
High (predicted)	1810011O10Rik	2.072
High (predicted)	4930402H24Rik	2.133
High (predicted)	Acsl1	2.076
High (predicted)	Acsl3	1.581
High (predicted)	Ak3	1.752
High (predicted)	Ak4	4.305
High (predicted)	Arhgef26	1.834
High (predicted)	Arpp19	1.623
High (predicted)	Atl2	1.636
High (predicted)	Atp6v1b2	1.730
High (predicted)	Bcat1	1.937
High (predicted)	Bnc2	1.773
High (predicted)	Coq10b	2.078
High (predicted)	Cpeb3	2.224
High (predicted)	Csrnp3	2.493
High (predicted)	Dbt	2.075
High (predicted)	Dgkg	2.075
High (predicted)	Dmxl2	1.625
High (predicted)	Dock5	1.652
High (predicted)	Eif4a2	2.102
High (predicted)	Enpp5	1.722
High (predicted)	Fam46a	1.617
High (predicted)	Fut9	2.323
High (predicted)	Gabarapl1	1.653
High (predicted)	Gpr137b	2.815
High (predicted)	Grsf1	1.664
High (predicted)	Hecw2	2.586
High (predicted)	Hlf	2.523
High (predicted)	Ifi44l	4.538
High (predicted)	Kcnc4	41.962
High (predicted)	Lrp2	3.489
High (predicted)	Lrrtm2	5.657
High (predicted)	Magi2	1.756
High (predicted)	Mapk14	1.525
High (predicted)	Mfap3l	1.788
High (predicted)	Mtus1	1.587
High (predicted)	Nanp	1.928
High (predicted)	Neurod1	10.098
High (predicted)	Nhs	1.983
High (predicted)	Papd4	1.674
High (predicted)	Pax6	10.098
High (predicted)	Pex5l	2.158
High (predicted)	Plag1	2.157
High (predicted)	Ppara	1.982
High (predicted)	Prkaa2	1.701
High (predicted)	Rhebl1	2.512
High (predicted)	Ric3	1.916
High (predicted)	Robo2	2.641
High (predicted)	Rtn1	2.160
High (predicted)	Sar1b	1.811
High (predicted)	Sec22a	2.199
High (predicted)	Sgk1	2.417
High (predicted)	Sgms2	1.666
High (predicted)	Slc26a4	2.474
High (predicted)	Slc26a7	2.367
High (predicted)	Slc8a1	2.450
High (predicted)	St8sia3	11.680
High (predicted)	Steap2	1.616
High (predicted)	Stxbp4	1.636
High (predicted)	Tmem64	1.739
High (predicted)	Tmem9b	1.661
High (predicted)	Trpc3	1.861
High (predicted)	Trps1	1.811
High (predicted)	Txlng	1.772
High (predicted)	Vstm2b	3.406
High (predicted)	Wipf3	1.948
High (predicted)	Wnk1	2.338
High (predicted)	Zhx3	1.634

miR-92a

Confidence	Gene	Fold Change
High (predicted)	4930402H24Rik	2.133
High (predicted)	Ahcyl1	1.621
High (predicted)	Aifm2	1.646
High (predicted)	Arpp19	1.623
High (predicted)	Astn1	5.544
High (predicted)	Atp6v1b2	1.730
High (predicted)	Bcl11b	1.880
High (predicted)	Chchd10	4.790
High (predicted)	Cldn11	5.191
High (predicted)	Cpeb3	2.224
High (predicted)	Csrnp3	2.493
High (predicted)	Dmxl1	1.659
High (predicted)	Dock5	1.652
High (predicted)	Dpyd	2.542
High (predicted)	Dynlt3	1.952
Experimentally Observed	Enpp6	3.840
High (predicted)	Eps8	1.848
High (predicted)	Gclm	1.722
High (predicted)	Gpr85	2.132
High (predicted)	Gria3	2.255
High (predicted)	Hand2	18.379
High (predicted)	Hoxc4	1.834
High (predicted)	Idh1	1.854
High (predicted)	Kcna1	4.451
High (predicted)	Kcnc4	41.962
High (predicted)	Kcnk3	4.448
High (predicted)	Klf2	1.897
High (predicted)	Lamp2	1.852
High (predicted)	Lurap1l	2.021
High (predicted)	Mcoln2	1.806
High (predicted)	Mier3	1.865
High (predicted)	Mpp1	2.099
High (predicted)	Mrps25	1.925
High (predicted)	Nox4	2.801
High (predicted)	Pax9	36.555
High (predicted)	Pex3	1.824
High (predicted)	Ppcs	2.170
High (predicted)	Prkar2b	5.035
High (predicted)	Ptprk	1.840
High (predicted)	Rab3b	1.852
High (predicted)	Rbpms2	1.703
High (predicted)	Robo2	2.641
High (predicted)	Sar1b	1.811
High (predicted)	Sgk1	2.417
High (predicted)	Slc24a4	9.952
High (predicted)	Slc25a36	1.751
High (predicted)	Sorcs3	2.934
High (predicted)	Sostdc1	2.657
High (predicted)	Syn2	1.844
High (predicted)	Taf15	1.858
High (predicted)	Tef	1.733
High (predicted)	Uqcrb	4.830

Supplementary Table 5: Differential Expression of miR-17~92 putative targets (TargetScan) in *Pkd2* mutant kidneys *(KO* vs. *DKO)*

miR-17

Confidence	Gene	Fold Change
High (predicted)	A1CF	1.397
High (predicted)	AHRR	2.664
High (predicted)	AK4	2.050
High (predicted)	ALDH1A3	2.354
Experimentally Observed	APP	1.081
High (predicted)	ATOH8	1.289
High (predicted)	ATP2B2	1.518
High (predicted)	BCL11B	1.355
High (predicted)	CAMTA2	1.124
High (predicted)	CDC37L1	1.121
High (predicted)	COL4A3	1.303
High (predicted)	CPEB3	1.433
Experimentally Observed	CRIM1	1.125
High (predicted)	CYP26B1	2.150
High (predicted)	FBXO21	1.147
High (predicted)	FGF12	1.414
High (predicted)	FOXQ1	1.473
High (predicted)	FZD7	1.184
High (predicted)	HLF	1.636
High (predicted)	ISM1	1.226
High (predicted)	LACE1	1.205
High (predicted)	LDLRAD3	1.221
High (predicted)	LRP4	1.164
High (predicted)	MFAP3L	1.156
High (predicted)	MGLL	1.340
High (predicted)	MINK1	1.107
High (predicted)	NABP1	1.208
High (predicted)	NAPEPLD	1.339
High (predicted)	NEURL1B	1.225
High (predicted)	NKIRAS1	1.197
High (predicted)	NRIP3	1.688
High (predicted)	NTRK3	1.705
High (predicted)	PFN2	1.170
High (predicted)	PPARA	1.372
High (predicted)	PRRG1	1.179
High (predicted)	PTGER3	1.514
High (predicted)	PTPRD	1.293
High (predicted)	RASL11B	1.223
High (predicted)	RBM24	2.021
High (predicted)	RORC	1.624
High (predicted)	SALL1	1.205
High (predicted)	SLC16A9	1.454
High (predicted)	SLC22A23	1.236
High (predicted)	SLC29A2	1.285
High (predicted)	SLC2A4	1.673
High (predicted)	SLC5A3	1.511
High (predicted)	SNX21	1.176
High (predicted)	STC1	1.426
High (predicted)	SYTL4	1.275
High (predicted)	TANC1	1.105
High (predicted)	TBX3	1.397
High (predicted)	THRB	1.338
High (predicted)	TMEM25	1.363
High (predicted)	TMTC1	1.311
High (predicted)	USP46	1.143
Experimentally Observed	VEGFA	1.278
High (predicted)	VLDLR	1.263
High (predicted)	WASF3	1.235
High (predicted)	WNK1	1.176
High (predicted)	ZFP3	1.155

miR-18a

Confidence	Gene	Fold Change
Moderate (predicted)	A1CF	1.397
Moderate (predicted)	ABCD3	1.363
Moderate (predicted)	ACAD11	1.260
Moderate (predicted)	ACO1	1.336
Moderate (predicted)	ADGRF1	1.836
Moderate (predicted)	ADRA1A	1.409
Moderate (predicted)	ALDH5A1	1.273
Moderate (predicted)	ANGPT2	1.427
Moderate (predicted)	AQP5	1.602
Moderate (predicted)	CNDP1	1.577
Moderate (predicted)	COL9A2	1.864
Moderate (predicted)	DLST	1.220
Moderate (predicted)	ECHS1	1.210
Moderate (predicted)	EDA	1.258
Moderate (predicted)	EYA4	1.726
Moderate (predicted)	FAM101A	1.188
Moderate (predicted)	FAM195A	1.443
Moderate (predicted)	FBXO21	1.147
Moderate (predicted)	FBXO9	1.132
Moderate (predicted)	FN3KRP	1.184
Moderate (predicted)	FXN	1.233
Moderate (predicted)	GLUL	1.310
Moderate (predicted)	GPAM	1.284
Moderate (predicted)	HADHA	1.143
Moderate (predicted)	HEXIM1	1.160
Moderate (predicted)	HNMT	1.226
Moderate (predicted)	HNMT	1.226
Moderate (predicted)	HSBP1L1	1.346
Moderate (predicted)	HYAL1	1.197
Moderate (predicted)	IDH3G	1.212
Moderate (predicted)	IL15	1.435
Moderate (predicted)	IL15	1.435
Moderate (predicted)	KL	1.446
Moderate (predicted)	ME1	1.240
Moderate (predicted)	MMAA	1.179
Moderate (predicted)	NCOA4	1.184
Moderate (predicted)	NDUFS1	1.253
Moderate (predicted)	NKIRAS1	1.197
Moderate (predicted)	PAPSS2	1.334
Moderate (predicted)	PAPSS2	1.334
Moderate (predicted)	PCGF2	1.125
Moderate (predicted)	PCSK6	2.042
Moderate (predicted)	PDE1A	1.170
Moderate (predicted)	PDE1A	1.170
Moderate (predicted)	PDE4DIP	1.135
Moderate (predicted)	PFKM	1.187
Moderate (predicted)	PRSS12	1.170
Moderate (predicted)	PRSS12	1.170
Moderate (predicted)	SCG3	3.627
Moderate (predicted)	SH3BGRL2	1.110
Moderate (predicted)	SLC16A10	1.281
Moderate (predicted)	SLC22A8	1.705
Moderate (predicted)	SLC34A1	1.608
Moderate (predicted)	SOWAHA	1.306
Moderate (predicted)	STARD8	1.240
Moderate (predicted)	STK32B	1.328
Moderate (predicted)	TCEA2	1.250
Moderate (predicted)	TM2D2	1.134
Moderate (predicted)	UQCRQ	1.219

miR-19b

Confidence	Gene	Fold Change
High (predicted)	ACADSB	1.208
High (predicted)	ACSL1	1.376
High (predicted)	AK3	1.124
High (predicted)	AK4	2.050
High (predicted)	AKAP1	1.137
High (predicted)	ATP2B2	1.518
High (predicted)	BCAT1	2.078
High (predicted)	CAB39L	1.182
High (predicted)	CADM4	1.213
High (predicted)	CISD3	1.257
High (predicted)	CPEB3	1.433
High (predicted)	CS	1.130
High (predicted)	CSPP1	1.120
High (predicted)	CXCL12	1.336
High (predicted)	DBT	1.248
High (predicted)	ELOVL5	1.147
High (predicted)	EMC7	1.106
High (predicted)	GPRC5B	1.238
High (predicted)	HLF	1.636
High (predicted)	HOXA9	1.268
High (predicted)	ICA1L	1.714
High (predicted)	MFAP3L	1.156
High (predicted)	MINK1	1.107
High (predicted)	MLEC	1.138
High (predicted)	MPRIP	1.091
High (predicted)	NEURL1B	1.225
High (predicted)	NUS1	1.242
High (predicted)	PCGF2	1.125
High (predicted)	PFN2	1.170
High (predicted)	PHACTR4	1.150
High (predicted)	PPARA	1.372
High (predicted)	PTPRD	1.293
High (predicted)	RNF167	1.214
High (predicted)	RTN4RL1	1.210
High (predicted)	SCN4B	1.551
High (predicted)	SGK1	1.702
High (predicted)	SLC26A7	1.454
High (predicted)	SLC35D1	1.233
High (predicted)	SV2A	1.557
High (predicted)	THRB	1.338
High (predicted)	TLN2	1.214
High (predicted)	TMEM25	1.363
High (predicted)	TOM1L2	1.138
High (predicted)	WIPF3	1.586
High (predicted)	WNK1	1.176
High (predicted)	WRNIP1	1.115

miR-92a

Confidence	Gene	Fold Change
High (predicted)	ASTN1	1.492
High (predicted)	ATP2A2	1.150
High (predicted)	BCAT2	1.271
High (predicted)	BCL11B	1.355
High (predicted)	CCNJL	1.166
High (predicted)	CEND1	1.791
High (predicted)	CLDN11	4.186
High (predicted)	CPEB3	1.433
High (predicted)	DBT	1.248
High (predicted)	DPYD	1.379
High (predicted)	EPS8	1.168
High (predicted)	FAM150B	1.802
High (predicted)	GPR85	1.266
High (predicted)	GRIP2	1.367
High (predicted)	HOXD10	1.202
High (predicted)	IDH1	1.291
High (predicted)	IQGAP2	1.182
High (predicted)	ITPR1	1.203
High (predicted)	KCNK3	2.426
High (predicted)	KLHL14	1.195
High (predicted)	MAP4K2	1.152
High (predicted)	MGLL	1.340
High (predicted)	MRPS25	1.169
High (predicted)	NEURL1B	1.225
High (predicted)	NFYC	1.236
High (predicted)	PDE4D	1.194
High (predicted)	PLEKHA1	1.120
High (predicted)	PTPRD	1.293
High (predicted)	RBM47	1.155
High (predicted)	RBPMS2	1.275
High (predicted)	SGK1	1.702
High (predicted)	SIM2	1.329
High (predicted)	SLC25A16	1.205
High (predicted)	SNN	1.103
High (predicted)	SOSTDC1	1.530
High (predicted)	TBX3	1.397
High (predicted)	TEF	1.282
High (predicted)	TMEM143	1.351
High (predicted)	WNT5A	1.514
High (predicted)	WRNIP1	1.115
High (predicted)	XPNPEP3	1.155
High (predicted)	ZBTB40	1.167
High (predicted)	ZFYVE21	1.289

Supplementary Table 6: Primers Used

Gene		Primer Sequence
Pkd1	Forward	5'- TCTCGGAGCAGAATCAATGCC-3'
	Reverse	5'-CAGAGTTGAGGGACAGTGAGAT-3'
Pkd2	Forward	5'-GCGTGGTACCCTCTTGGCAGTT-3'
	Reverse	5'-CACGACAATCACAACATCC-3'
Kim1	Forward	5'-AGCAGTCGGTACAACTTAAAGG-3'
	Reverse	5'-AGAGTTCTCTATCGTCAAGGACA-3'
Ngal	Forward	5'-GCAGGTGGTACGTTGTGGG-3'
	Reverse	5'-CTCTTGTAGCTCATAGATGGTGC-3'
Acta2	Forward	5'-GTCCCAGACATCAGGGAGTAA-3'
	Reverse	5'-TCGGATACTTCAGCGTCAGGA-3'
Vim	Forward	5'-CGTCCACACGCACCTACAG-3'
	Reverse	5'-GGGGGATGAGGAATAGAGGCT-3'
Col4a6	Forward	5'-GCACCCTCACAAGCCATT-3'
	Reverse	5'-CCAGGGGAGACTAGGGACTG-3'
Col1a1	Forward	5'-GCTCCTCTTAGGGGCCACT-3'
	Reverse	5'-CCACGTCTCACCATTGGGG-3'
Ppara	Forward	5'-CCTCAAAGTCTGAGCGGTCT-3'
	Reverse	5'-CTAACCTTGGGCCACACCT-3'
Ppargc1a	Forward	5'-GTGAACATTCAAAGCAGCAGAG-3'
	Reverse	5'-TTCTTCGTACAGCCATCAAAAA-3'
Pparg	Forward	5'-AGCTCCAAGAATACCAAAGTGC-3'
	Reverse	5'-GATGCTTTATCCCAGACTC-3'
Sod2	Forward	5'-CAGACCTGCCTTACGACTATGG-3'
	Reverse	5'-CTCGGTGGCGTTGAGATTGT-3'
Me1	Forward	5'-GTCGTGCATCTCTCACAGAAG-3'
	Reverse	5'-TGAGGGCAGTTGGTTTTATCTTT-3'
Oxct1	Forward	5'-CATAAGGGGTGTGTCTGCTACT-3'
	Reverse	5'-GCAAGGTTGCACCATTAGGAAT-3'
Pdk4	Forward	5'-AGGGAGGTCGAGCTGTTCTC-3'
	Reverse	5'-GGAGTGTTCACTAAGCGGTCA-3'
Cpt2	Forward	5'-CAGCACAGCATCGTACCCA-3'
	Reverse	5'-TCCCAATGCCGTTCTCAAAAT-3'
Slc27a2	Forward	5'-CGAGACGAGACGCTCACCTA-3'
	Reverse	5'-ACGAATGTTGTAGTTGAGGCAC-3'
Cd36	Forward	5'-ATGGGCTGTGATCGGAACTG-3'
	Reverse	5'-TTTGCCACGTCATCTGGGTTT-3'
Etfdh	Forward	5'-GTGCGACTAACCCTGTC-3'
	Reverse	5'-GGATGAACAGTGTAGTGAGTGG-3'
Etfa	Forward	5'-GCCTCATTGCTCCGTTTTCAG-3'
	Reverse	5'-GCTACTAAGCAGGACACTTCAC-3'
Etfb	Forward	5'-CTGTCAAGAGGTCATCGACT-3'
	Reverse	5'-CACAGAAGGGGTTCATGGAGT-3'
Cmyc	Forward	5'-CTGAGCCCCTAGTGCTGCATGA3'
	Reverse	5'-GGGGTTTGCCTCTTCTCCACAG-3'
hPPARA	Forward	5'-GGAGTTTATGAGGCCATATTCG-3'
	Reverse	5'-CAAAATCAAACTTGGGTTCCAT-3'

Supplementary References:

1. Androsavich JR, *et al.* Polysome shift assay for direct measurement of miRNA inhibition by anti-miRNA drugs. *Nucleic Acids Res* **44**, e13 (2016).

2. Song X, *et al.* Systems Biology of Autosomal Dominant Polycystic Kidney Disease (ADPKD): Computational Identification of Gene Expression Pathways and Integrated Regulatory Networks. *Hum Mol Genet*, (2009).
